# On the Use of Triarylsilanols as Catalysts for Direct Amidation of Carboxylic Acids

**DOI:** 10.1021/acs.joc.3c00585

**Published:** 2023-07-11

**Authors:** D. Christopher Braddock, Ben C. Rowley, Paul D. Lickiss, Steven J. Fussell, Rabia Qamar, David Pugh, Henry S. Rzepa, Andrew J. P. White

**Affiliations:** † Department of Chemistry, Imperial College London, Molecular Sciences Research Hub, White City Campus, 82 Wood Lane, London W12 0BZ, U.K.; ‡ Pfizer Ltd., Ramsgate Road, Sandwich, Kent CT13 9NJ, U.K.; § Department of Chemistry, King’s College London, Britannia House, 7 Trinity Street, London SE1 1DB, U.K.

## Abstract

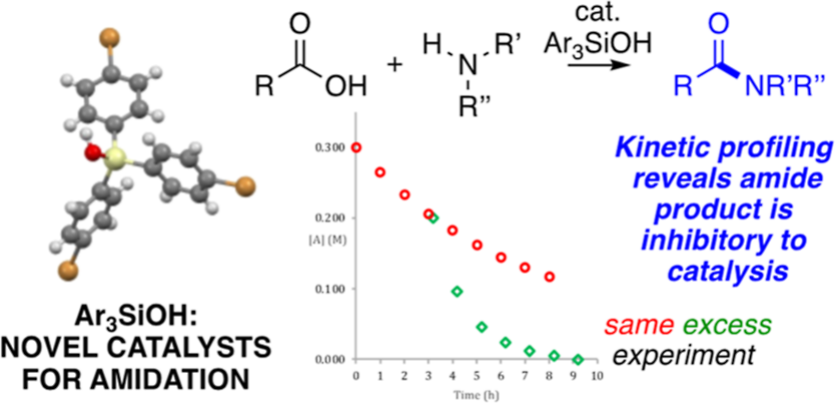

Triarylsilanols have been reported as the first silicon-centered molecular catalysts for direct amidation of carboxylic acids with amines as identified after a screen of silanols, silanediols, disiloxanediols, and incompletely condensed silsesquioxanes as potential homogeneous catalysts. Subsequent synthesis and testing of various electronically differentiated triarylsilanols have identified tris(*p*-haloaryl)silanols as more active than the parent triarylsilanol, where the bromide congener is found to be the most active. Catalyst decomposition can be observed by NMR methods, but RPKA methods reveal that product inhibition is operative, where tertiary amides are more inhibitory than secondary amides. Studies using an authentically synthesized triaryl silylester as a putative intermediate in the catalytic system enable a plausible mechanism to be proposed as supported by computationals.

## Introduction

The catalytic amidation of carboxylic acids^1^ thereby avoiding stoichiometric quantities of activating agent^2^ is the focus of much current research.^3^ Significant progress has been made with the development of boron-based catalysts,^4−7^ oxophilic metal catalysts,^8^ and other catalytic systems.^9^ However, while there are several reports of successful silicon-based reagents^10^ and silica gels^11^ for direct amidation, there are no examples of silicon-centered molecular catalysts for direct amidations.^12^ Furthermore, while various proposals for the mechanistic operation of catalytic amidations have been moot,^4−9^ only limited direct experimental evidence for presumed activated intermediates has been garnered.^13^ Herein, we report on the discovery, synthesis, and use of various electronically differentiated triarylsilanols as the first silicon-centered molecular catalysts for direct amidation. Kinetic profiling reveals that these reactions are subject to product inhibition and also sheds light on the reactivity differences between different types of acid–amine combinations. Moreover, we also report on the preparation and utilization of catalytically competent intermediates including an expected on-cycle triarylsilyl ester and an unexpected off-cycle silanaminium salt.

## Results and Discussion

**Figure 1:**
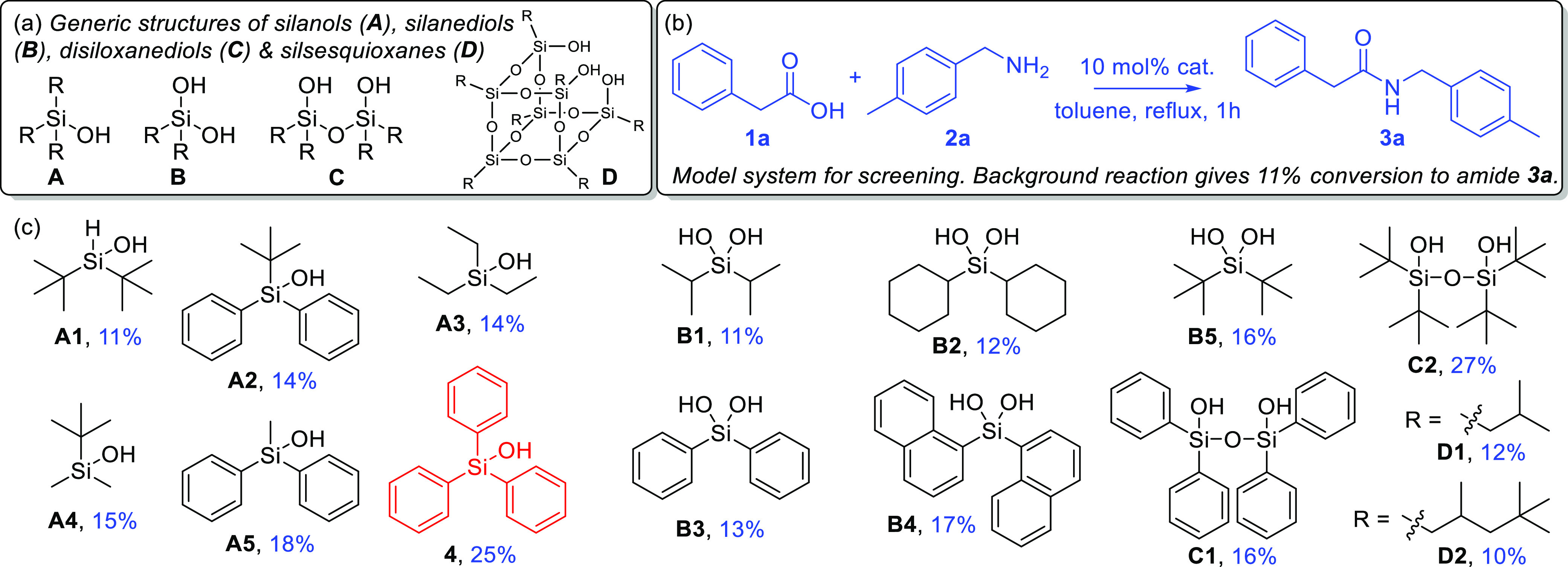
(a) Generic structures of silica molecular analogues **A–D**, (b) the model amidation reaction to give amide **3a**, and (c) a screen of potential catalysts with percentage conversions for the model amidation.

On the basis that silica gels have been successfully employed for direct amidation of carboxylic acids (vide supra), we considered that silanols **A**, silanediols **B**, disiloxanediols **C**, and incompletely condensed silsesquioxanes **D** as molecular analogues of silica gel may also be proficient as homogeneous catalysts (Figure 1a).^14^ Accordingly, an initial screen of such species at 10 mol % loading using a model amidation of aliphatic carboxylic acid **1a** with primary amine **2a** in the ideal 1:1 stoichiometry to give amide **3a** (Figure 1b) for 1 h in refluxing toluene was conducted (Figure 1c).^15^ In the event, once the background conversion (11%) with no added catalyst had been taken into account for this acid–amine combination, the 25% conversion obtained with triphenylsilanol **4** revealed it to be a lead catalyst for further study.^16^ Pleasingly, and in contrast to the other putative silanol, silanediol, and disiloxanediols catalysts that were screened, no irreversible condensation of triphenylsilanol **4** to catalytically unreactive hexaphenyldisiloxane [(Ph_3_Si)_2_O] was observed under these conditions.^17,18^

**Figure 2:**
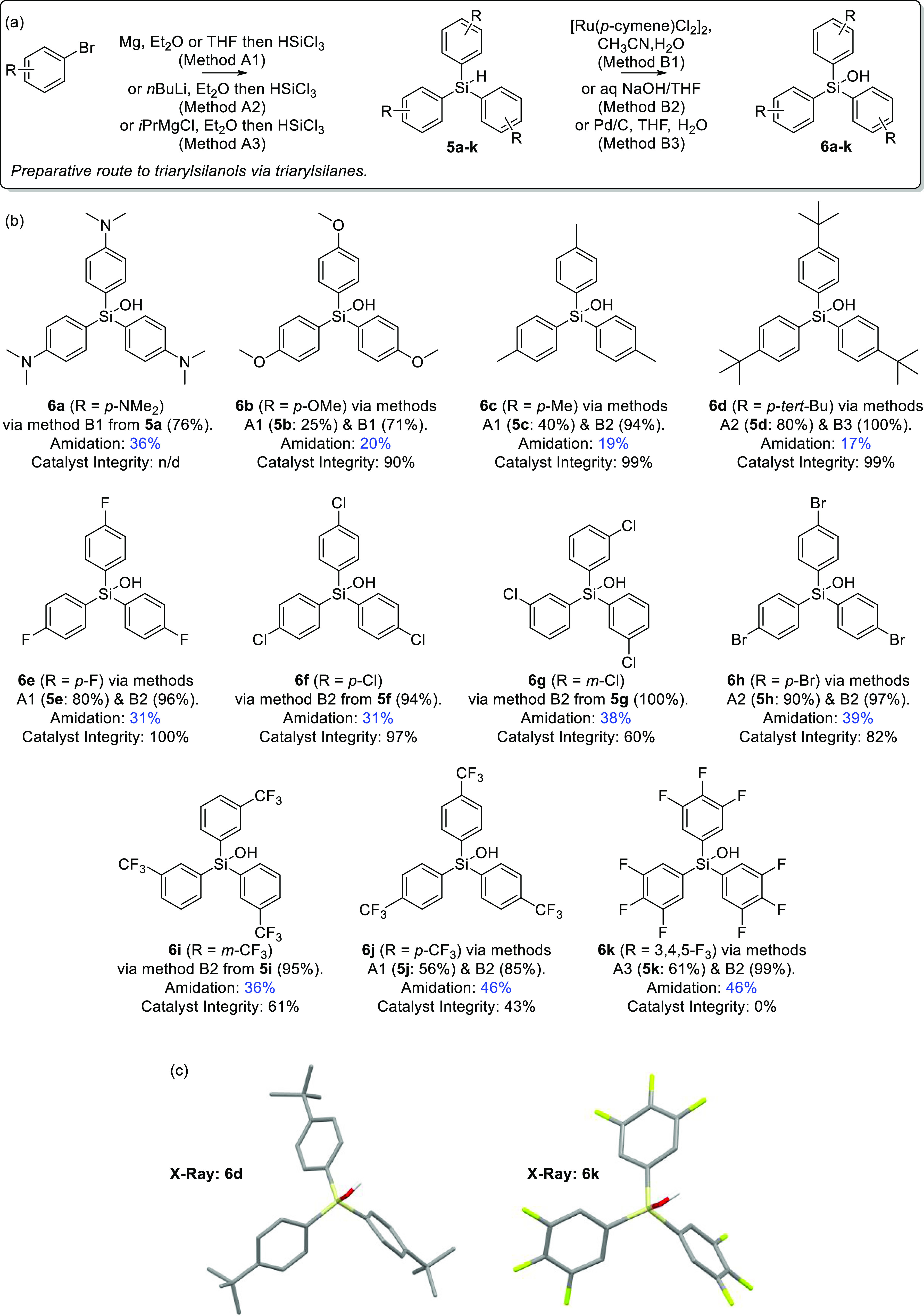
(a) Preparative route to silanes **5a–k** and silanols **6a–k**, (b) a screen of the catalytic activity of silanols **6a–k** in the model amidation reaction (cf., Figure 1b) including an assessment of catalyst integrity post-amidation, and (c) X-ray crystal structures of novel silanols **6d** and **6k**.

As the next stage of the investigation, a series of known (**5a–j**)^19,20^ and new (**5k**) electronically differentiated triaryl silanes were prepared via metalation of the appropriate aryl bromide followed by reaction with trichlorosilane (Figure 2a). Subsequent hydrolysis provided known (**6a**, **6c**, **6e**, **6f**, and **6h**)^21^ and new (**6b**, **6d**, **6g**, and **6i–k**) silanols as potential amidation catalysts.^22^ Each silanol was then screened against the model amidation reaction (cf., Figure 1b) for its catalytic activity (Figure 2b). In addition, an assessment of catalyst integrity post-amidation was performed by ^1^H NMR analysis compared to an internal standard. Here, the concern is that condensation to the corresponding (assumed) catalytically inactive disiloxane (R_3_SiOSiR_3_)^23^ and/or other processes that cleave the Si–aryl bond(s) may occur, although this analysis did not allow the distinction between these two different possibilities.

**Figure 3:**
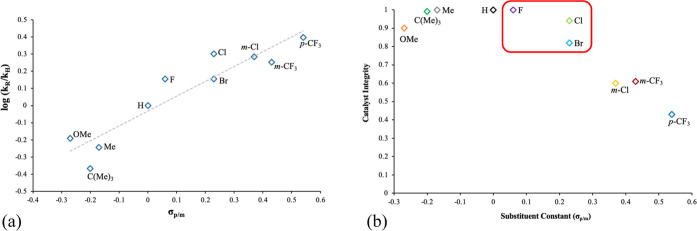
(a, left) Hammett plot for catalytic amidation using catalysts **6b–j** and **4**, and (b, right) plot of substituent constants (σ_p/m_) vs catalyst integrity post-amidation. The red box denotes the catalysts selected for further study.

Inspection of the results for silanol catalysts **6a–k** shows that under the specified conditions of the model reaction (10 mol % catalyst, 1h reaction time), increased conversions to amide **3a** (**6j**, **6k**: 46%; cf., **4**: 25%) can be obtained. Further inspection reveals a trend for increased activity for those catalysts bearing electron-withdrawing groups (and reduced activity for those with electron donating groups). Indeed, a Hammett plot of substituent constant^24^ vs log(*k*_R_/*k*_H_)^25^ for silanols **6b–j** (Figure 3a) yields a value of ρ = +0.82,^26^ implicating the buildup of negative charge in the rate determining step of this catalytic amidation but also signifying that the aryl groups are somewhat remote from the location of the charge. This implies that additional and/or more strongly electron-withdrawing groups should further improve the catalytic activity. Unfortunately, while triphenylsilanol **4** itself (σ_p_ = 0) and alkyl substituted silanols **6c–d** (σ_p_ ca. −0.2) did not display any detectable catalyst decomposition,^27^ inspection of the catalyst integrity data for electron-deficient silanols **6e–j** shows that the onset of catalyst decomposition under these conditions has already begun, ranging from slight-to-moderate (0.1 ≥ σ_p/m_ ≥ 0.3) to severe (0.4 ≥ σ_p/m_ ≥ 0.6) (Figure 3b). This trend is continued for strongly electron-deficient tris(trifluorophenyl)silanol **6k** (not shown in Figure 3b), which, while giving the maximum observed conversion in this catalyst screen, resulted in complete catalyst decomposition. This, therefore, effectively marks the limit of electron-withdrawing substituents that can be introduced into these silanols for use in such direct amidation reactions. Moreover, inspection of the results for strongly electron-rich substituents (σ_p_ < −0.2) show that non-negligible amounts of catalyst decomposition are observable here too, as exemplified by *p*-methoxy silanol **6b**. These results suggest that more than one pathway of catalyst deactivation is in operation. This is consistent with previous studies where it is known that electron donating groups enhance the aryl–silicon bond cleavage of triarylsilanes under acidic conditions, whereas strongly electron-deficient triarylsilanes rapidly decompose under the action of base.^28^

**Figure 4:**
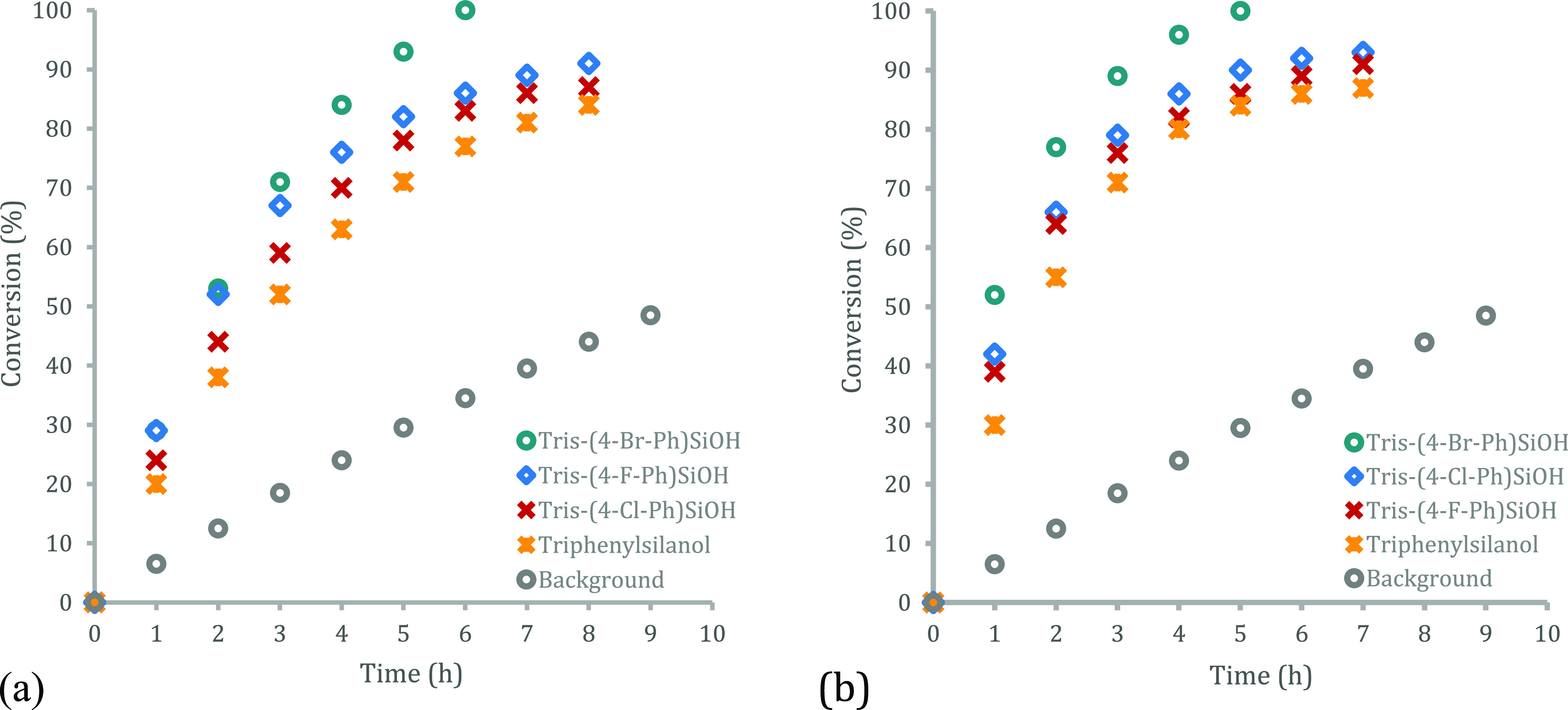
Conversion vs time plots for the model amidation reaction (cf., Figure 1b) with triarylsilanol catalysts **4**, **6e**, **6f**, and **6h** at (a, left) 10 mol %, (b, right) and 30 mol % catalyst loadings.

On the basis of their improved performance compared to parent silanol **4**, counterbalanced by only slight-to-moderate loss of catalyst integrity in the range (0.1 ≥ σ_p/m_ ≥ 0.3), the tris(*p*-haloaryl)silanols **6e** (*p*-F), **6f** (*p*-Cl) and **6h** (*p*-Br) were selected for further study, where—of those selected—silanol **6h** had been found to provide the highest conversion to amide **3a** after 1h reaction time (cf., Figure 2). A comparison of conversion versus time profiles at 10 mol % (Figure 4a) and 30 mol % (Figure 4b) loading for these different catalysts for the model amidation reveals two main features. First, bromide congener **6h** displays superior catalytic activity at both loadings and over the entire course of the reaction. Notably, at 30 mol % loading of *p*-bromo silanol **6h**, the initial rate is ca. 10 times faster than the background reaction, and quantitative conversion to amide **3a** was observed after 6h. Based on this finding, we selected 30 mol % catalyst loading as our standard loading for further studies (vide infra). Second, we note that the increased loadings give increased initial rates with all catalysts, although the responses are not linear and is indicative of complex kinetic behavior (vide infra).

We next elected to explore preparative amidation reactions using 30 mol % catalyst **6h**. Phenylacetic acid and benzoic acid were chosen as representative alkyl and aryl carboxylic acids to undergo attempted catalytic amidation with bromosilanol **6h**, where aromatic carboxylic acids are known to be more difficult to amidate compared to aliphatic ones.^10j^ 4-Methylbenzylamine, morpholine, and *N*-methylbenzylamine were chosen as representative primary, cyclic, and acyclic secondary amines, which are increasingly resistant to amidation. Aniline was also selected as an aromatic amine with reduced nucleophilicity. These acid–amine combinations allow benchmarking against catalytic amidation reactions in the literature.

**Figure 5:**
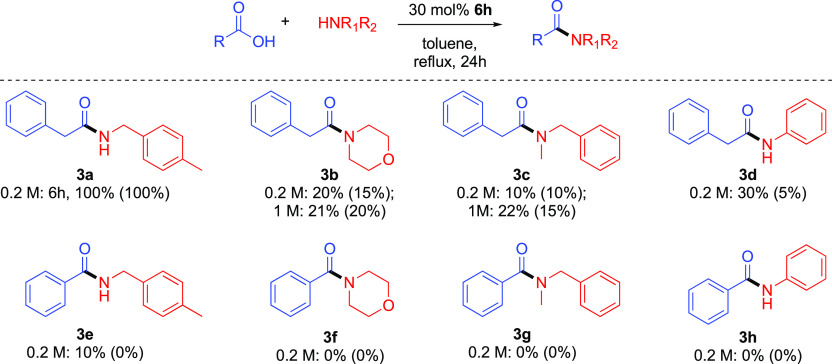
Substrate scope of catalytic amidation with bromosilanol **6h** at 0.2 or 1.0 M concentration in both acid and amine with isolated yields after acid–base workup and dry column vacuum chromatography. The figures in parentheses are isolated yield of a background reaction without catalyst, where the amide products were pure after acid–base workup. Amide **3a** precipitated from the reaction mixture on cooling and could be collected by filtration. Amide **3d** was triturated with *n*-hexane to remove unreacted aniline.

There are very few reports on high-yielding *catalytic* amidation reactions of aromatic carboxylic acids in the ideal 1:1 acid/amine stoichiometry in the literature,^4−9^ and perhaps unsurprisingly, catalytic quantities of bromosilanol **6h** failed to provide any quantities of amides **3f–3h**, which are among the most difficult combinations of acid and amine (Figure 5). Pleasingly, some activity was found for the formation of aromatic amide **3e** albeit in low conversion after 24 h. Aliphatic amide **3a** was formed in quantitative yield in a reaction time of 6 h, albeit where the background reaction is itself complete in 24 h. This result demonstrates the need to run background reactions when reporting direct amidation reactions. For tertiary amides **3b** and **3c**, some catalytic activity was observed over the background reaction rate, but the acceleration was only modest, and increasing the concentration did not ameliorate the situation. In these cases, after evaporative removal of the toluene solvent as the first step in the workup procedure, quantities of a white solid in a yellow oil was observed which, when isolated, proved to be hexa(4-bromophenyl)disiloxane,^29^ characterized for the first time by crystallography (see the Supporting Information), thereby implicating catalyst deactivation by condensation. In contrast, for anilide **3d**, the catalyst activity over the background reaction was more marked compared with what might have been expected, with the more nucleophilic secondary amines giving rise to amides **3b** and **3c**. With these admittedly disappointing results in hand, we sought to conduct additional kinetic experiments to interrogate the system further.^30,31^

**Figure 6:**
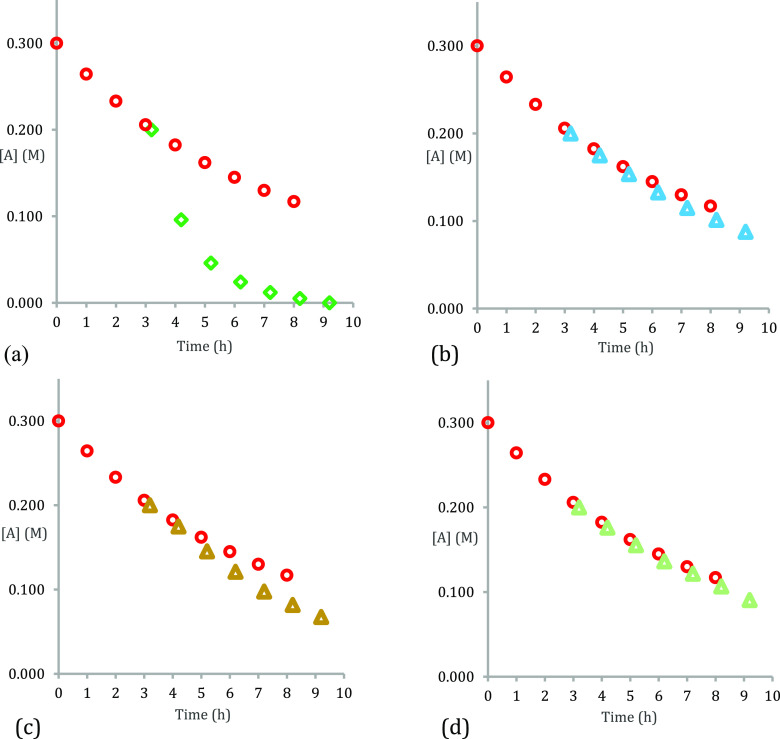
“Same excess” concentration vs time plots for the model amidation reaction (cf., Figure 1b) (a, top left) red circles *t*_0_ = 0.3 M [**1a**], 0.3 M [**2a**], and 0.06 M [**6h**] and time-adjusted green diamonds *t*_3.2_ = 0.2 M [**1a**], 0.2 M [**2a**], and 0.06 M [**6h**]; (b, top right) blue triangles with added 0.1 M [**3a**]; (c, bottom left) brown triangles with added 0.1 M [H_2_O]; and (d, bottom right) green triangles with added 0.1 M [**3a**] amide and 0.1 M [H_2_O].

Accordingly, a “same excess” experiment^32^ using catalyst **6h** for the model system (cf., Figure 1a) and time-adjusted analysis^33^ showed that the reaction profiles did not overlay (Figure 6a), in line with our expectations regarding the previously observed loss of catalyst integrity as determined by NMR spectroscopy (cf., Figures 2 and 3) and by the observation of disiloxane formation when using secondary amines. However, a second same excess experiment with added product amide **3a** gave plots that nearly overlaid (Figure 6b), showing instead that for this acid–amine combination, amide product inhibition is the dominant factor at play rather than catalyst decomposition, and these findings are also consistent with the determined catalyst order.^31^ A further reaction with water added instead (Figure 6c) shows that it also contributes to the inhibition (although we expect it is lost as it is generated in refluxing toluene). A fourth experiment (Figure 6d) with both added water and amide gave plots that almost perfectly overlaid. The very slight divergence of this latter plot can therefore be attributed to the catalyst decomposition.

**Figure 7:**
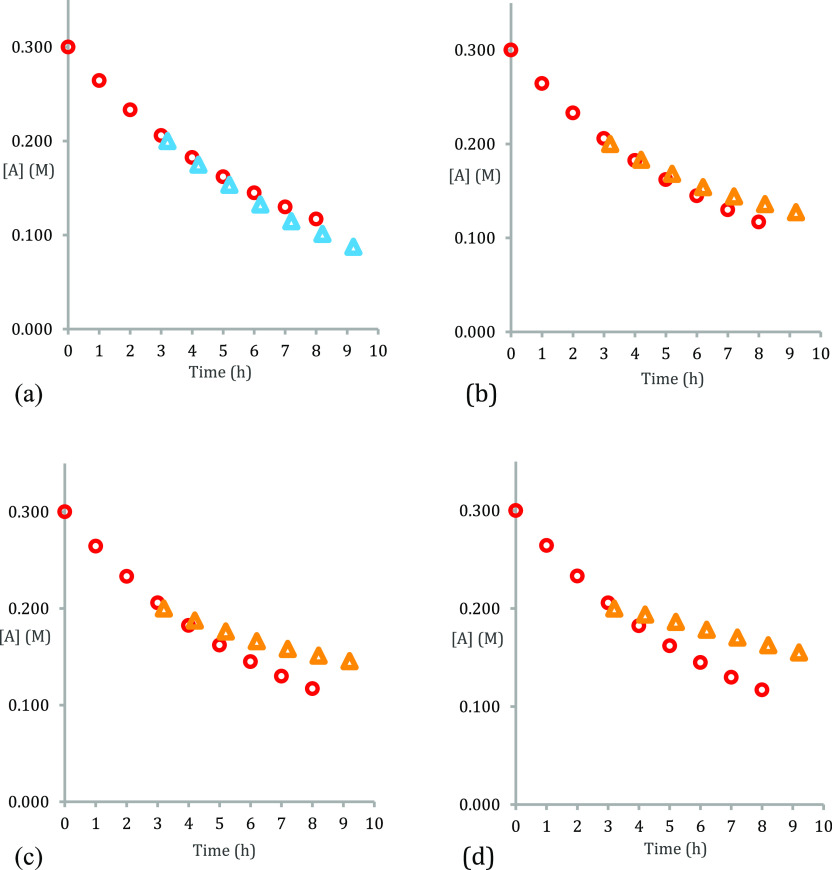
“Same excess” concentration vs time plots for the model amidation reaction (cf., Figure 1b) (a, top left) red circles: *t*_0_ = 0.3 M [**1a**], 0.3 M [**2a**], and 0.06 M [**6h**]; time-adjusted blue diamonds: *t*_3.2_ = 0.2 M [**1a**], 0.2 M [**2a**], 0.06 M [**6h**], and 0.1 M [**3a**]; (b, top right) orange triangles with added 0.1 M [**3d**] amide; (c, bottom left) orange triangles with added 0.1 M [**3c**]; and (d, bottom right) orange triangles with added 0.1 M [**3b**] amide and 0.1 M [H_2_O].

With this system being established, it was realized that it could be exploited to examine any inhibitory effect of *different amides* without the need to establish new conditions (Figure 7). Accordingly, each of anilide **3d** and tertiary amides **3b** and **3c** were individually added in further same excess experiments. Inspection of the plots reveal that anilide **3d** more strongly inhibits the reaction than amide product **3a** itself (Figure 7b). Tertiary amides **3b** and **3c** (Figure 7d,c respectively) inhibit the reaction even more strongly. The experiments, therefore, demonstrate that while it is to be expected that tertiary amides are inherently harder to form than secondary amides, to their reinforcing detriment, tertiary amide products inhibit the reaction more strongly than secondary amides. These results, therefore, go some way to rationalize the marked differences in reactivity in the catalytic formation of secondary amide **3a** versus tertiary amides **3b** and **3c** (cf., Figure 5).

Turning now to the question of catalyst decomposition quantification, ^1^H NMR methods had been used in the initial screening of arylsilanols (cf., Figure 2) to quantify catalyst integrity but suffered from overlapping resonances of catalyst, disiloxane, substrates, and products in the aromatic region of the spectrum. In an effort to overcome this difficulty, a HPLC method was developed for ex situ monitoring of catalyst integrity. Catalyst integrity monitoring experiments (0.06 M **6h** in toluene) at reflux, on its own, and with the individual components (0.2 M in acid, amine, or amide) of the model reaction (cf., Figure 1b) showed that the silanol was thermally stable, acid and amide were individually tolerated, and the catalyst concentration did not decrease at all, but the use of primary amine **2a** showed ca. 20% catalyst depletion to 0.050 M after 8 h at reflux (see the Supporting Information for data). These experiments show that silanol **6h** is sensitive to base-mediated decomposition. For secondary amine *N*-methylbenzylamine, the catalyst concentration reduced to 0.042 M (ca. 30% catalyst depletion), consistent with the increased basicity of secondary alkyl amines over primary alkyl amines. In contrast, for the same experiment employing aniline, 97% catalyst integrity (0.058 M) was observed at the end of the same period, consistent with its reduced basicity.

**Figure 8:**
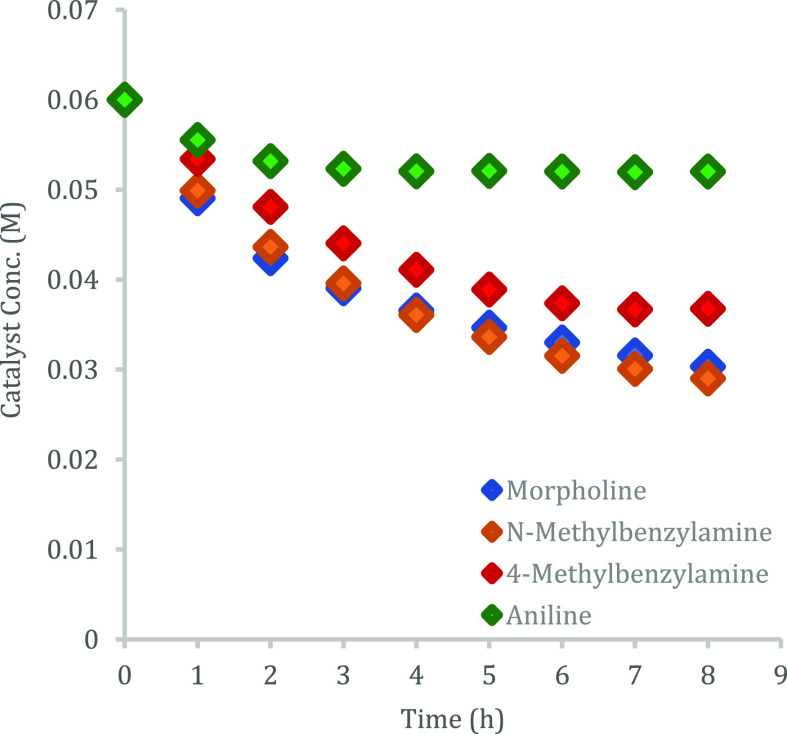
Plot of [**6h**] vs time in catalytic amidation reactions of phenyl acetic acid with 4-methylbenzylamine, *N*-methylbenzylamine, morpholine, and aniline as monitored by HPLC methods.

Thus, in amidation reactions catalyzed by silanol **6h**, it is to be expected that secondary alkyl amines will contribute to more extensive catalyst decomposition than primary alkyl amines. Thus, a second, detrimentally reinforcing—and perhaps now dominant— factor for these amine types is expected to be at play and further explains the poor catalytic conversions of secondary amines into tertiary amides **3b** and **3c** (cf., Figure 5). In the case of aniline, catalyst decomposition is expected to be less important and rationalizes the modest catalytic activity observed despite aniline’s lower nucleophilicity. In the event, ex situ HPLC monitoring of catalytic amidation runs of phenylacetic acid with 4-methylbenzylamine, morpholine, *N*-methylbenzylamine, and aniline to give amides **3a**–**3d** with 39, 49, 52, and 13% catalyst depletion, respectively, after 8 h refluxing in toluene (Figure 8). Evidently, under the conditions of the amidation, more severe catalyst depletion occurs than with each individual amine alone. We infer that acid and amine must act in conjunction to effect catalyst decomposition, and the more basic the amine, the more severe the depletion. Catalyst decomposition in the case of secondary amines is further accentuated by prolonged high concentrations of acid and base since the conversion to tertiary amide products is low.

**Scheme 1:**
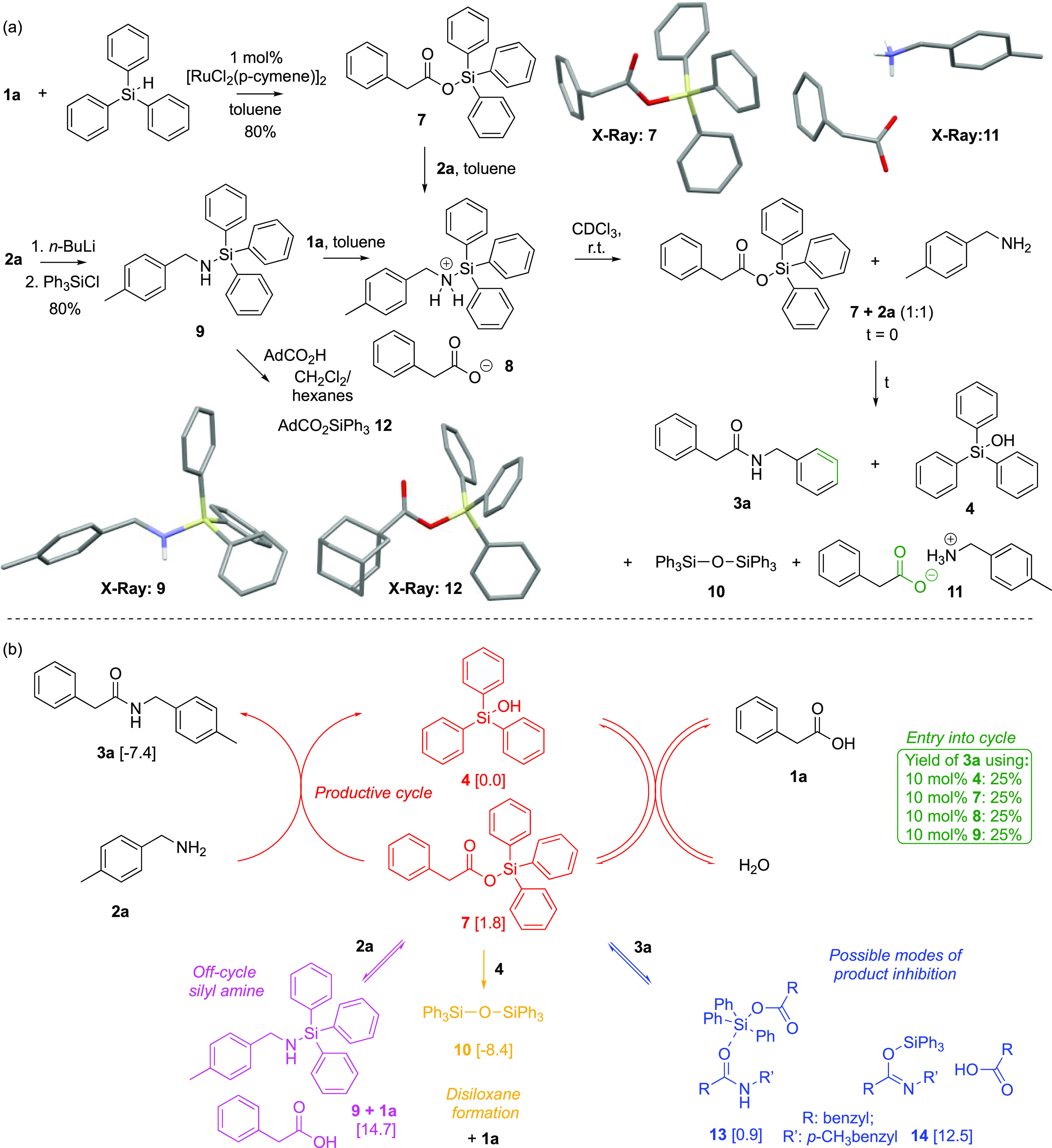
(a, Top) Preparation of Silyl Ester **7** and Silyl Amine **9** and Chemistry of the Resulting Silanaminium Carboxylate Salt **8** and (b, Bottom) Plausible Catalytic Cycle Numbers in square brackets are computed relative free energies Δ*G*_384_ in kcal/mol @ 4 atm. standard state (∼0.13 M) for discrete species involved in the catalytic cycle using the B3LYP+GD3+BJ/Def-TZVPP/SCRF = toluene density functional procedure (see ref (37)).

To elucidate the mechanism of these triarylsilanol-catalyzed direct amidation reactions, silyl ester **7**—as a putative intermediate^34^ in the catalytic cycle—was prepared by the modification of a known procedure from triphenylsilane^35^ (Scheme 1a). Attempted formation of amide **3a** via the *stoichiometric* reaction of silyl ester **7** with amine **2a** in toluene at room temperature led unexpectedly instead to the immediate formation of a white precipitate, which we formulate as silanaminium carboxylate salt **8**.^36^ In support of this formulation, a precipitate that behaved identically was obtained when silyl amine **9** was combined in a stoichiometric fashion with carboxylic acid **1a**. To our surprise, redissolution of salt **8** in rigorously dried chloroform-*d* gave a clean 1:1 mixture of ester **7** and amine **2a**. This mixture evolved over several days at room temperature to produce a mixture of amide **3a**, silanol **4**, disiloxane **10**, and ammonium carboxylate salt **11**. Here, we invoke productive attack of amine **2a** on ester **7** to give amide **3a**, thereby releasing free silanol **4**. Amine-mediated condensation of silanol **4** with a second equivalent of ester evidently allows the formation of disiloxane **10**, thereby concomitantly producing the ammonium carboxylate salt **11**. In accord with these observations, attempts to crystallize the silanaminium salt **8** from toluene, chloroform, or dichloromethane provided only ammonium carboxylate salt **11**. In contrast, the mixing of adamantane-1-carboxylic acid with silyl amine **9** allowed for the crystallization (CH_2_Cl_2_/hexane) of adamantyl silyl ester **12**. Inspection of the residual solution showed that no amide formation had occurred, showing that all the other downstream species must arise from the initial nucleophilic attack on silyl ester **7** (itself immediately regenerated on dissolution of the silanaminium salt **8**), where attack of **12** is prohibited under these conditions for steric reasons. These experiments, therefore, implicate silyl ester **7** as the key intermediate in the formation of amide **3a**, which is also evidently energetically accessible from silanol **4** under the conditions of the catalytic reactions and supported by computations.^37^ We invoke a mechanistic scenario that allows for any Lewis basic lone pair in the system to attack at the acyl carbon or at the silicon center of silyl ester **7** (Scheme 1b). Thus, attack at the acyl carbon by amine **2a** irreversibly gives product **3a**, whereas attack at Si gives silyl amine **9**; attack by water at either C or Si return silanol **4** and carboxylic acid **1a**; attack by silanol **4** at Si irreversibly gives disiloxane **10**, while attack at C is a degenerate exchange. Computations also helped to identify the possible mechanisms for amide inhibition as a Lewis base by interaction with the Si center; two of these are shown in Scheme 1b as amide-silyl ester dispersion complex **13** and silyl imidate **14**.^37,38^ The high calculated free energy of silyl imidate **14** does not support an inhibitory mechanism, whereas dispersion complex **13** is a better candidate. Finally, the use of *catalytic* quantities (10 mol %) of either silyl ester **7**, silanaminium salt **8** or silyl amine **9** were *all* found to provide identical levels of conversion in the model reaction as triphenylsilanol **4** itself (cf., Figure 1b) and is consistent with our mechanistic proposal.^39^

## Conclusions

In conclusion, we have discovered that triarylsilanols act as the first silicon-centered catalysts for direct amidation of carboxylic acids with amines. Synthesis and screening of a range of electronically differentiated triaryl silanols identified tris(*p*-haloaryl)silanols as more active catalysts than the parent triphenylsilanol, where the bromide congener was found to be the most active. Although catalyst degradation had been observed by inspection of the final product mixtures by NMR spectroscopy, same excess experiments instead identify amide product inhibition as the dominant detrimental effect in these catalytic runs, and is testament to the power of RPKA methods. Given that most catalytic direct amidation reactions involve Lewis acidic catalysts, Lewis basic amide product inhibition may be widespread.^13b^ Tertiary amide products were found to be more inhibitory than secondary amide products, thereby detrimentally reinforcing the inherent lower reactivity of secondary amines compared to primary amines in direct amidation reactions. As observed above, we expect this effect to be universal in other direct amidation reactions catalyzed by Lewis acidic catalysts. In addition, the silanol catalyst was found to be subject to condensation to catalytically inactive disiloxane under the reaction conditions, the extent of which correlated with the basicity of the amine employed. Since secondary alkyl amines are more basic than primary alkyl amines, this further detrimentally impacts on the use of secondary amines to form tertiary amide products. Studies using an authentically synthesized triaryl silylester as a putative intermediate in the catalytic system, and supported by calculations, enable a plausible mechanism of amidation to be proposed whereby the Lewis acidic silicon and acyl carbon centers of the silyl ester are able to interact with any Lewis basic species, including the amide product.

## Experimental Section

### General Considerations

Unless otherwise noted, reagents were purchased from commercial sources and used as received. Di-*t*-butylhydridosilanol (**A1**),^40^ dicyclohexanesilanediol (**B2**),^41^ 1,1,3,3-tetra-*t*-butyldisiloxane-1,3-diol (**C2**),^42^ tris(4-*N*,*N*-dimethylaminophenyl)silane (**5a**),^19a^ tris(4-chlorophenyl)silane (**5f**),^19d^ tris(3-chlorophenyl)silane (**5g**),^19e^ tris(3-(trifluoromethyl)phenyl)silane (**5i**),^19g^ and tetrakis(trimethylsilyl)methane were available as previously synthesized legacy chemicals in the Lickiss Group, Imperial College London. *t*-Butyldimethylsilanol (**A4**), diphenylsilanediol (**B3**), and 1,1,3,3-tetraphenyldisiloxane-1,3-diol (**C1**), incompletely condensed silsesquioxanes **D1** and **D2** and triphenylsilanol (**4**) were commercially available and used as received. 4-Methylbenzylamine was distilled under reduced pressure from CaH_2_ and stored in Schlenk tubes over activated 4 Å molecular sieves.

All reactions were carried out in oven-dried glassware, under an inert atmosphere of nitrogen, unless otherwise stated. Reaction temperatures other than ambient temperature were achieved using DrySyn heating blocks, dry ice/acetone bath (−78 °C), and ice/water bath (0 °C). Volatiles were removed in vacuo by rotary evaporation. Kieselgel-60 F254 pre-coated aluminum-backed plates were used for analytical thin layer chromatography and visualized using UV light (254 nm) or chemical staining with basic aqueous potassium permanganate solution.

^1^H NMR (400 MHz), ^13^C{^1^H} NMR (101MHz), ^19^F/^19^F{^1^H} NMR (377 MHz), and ^29^Si{^1^H} NMR (80 MHz) spectra were recorded at 298 K on a Bruker AV400 spectrometer. Chemical shifts (δ) are reported in ppm relative to solvent signals (δ = 7.26 and 77.16 ppm for CDCl_3_). Coupling constants (*J*) are quoted in Hz. Abbreviations used for multiplicity are as follows: s—singlet, d—doublet, dd—doublet of doublets, ddd—doublet of doublet of doublets, ddt—doublet of doublet of triplets, t—triplet, tt—triplet of triplets, app.t—apparent triplet, q—quartet, app. quint—apparent quintet, br—broad, m—multiplet. Structural assignments were made with additional information from gCOSY, gHSQC, and gHMBC experiments. Melting points were measured using a Lambda Photometrics MPA 100 OptiMelt melting point apparatus. High-resolution mass spectroscopy (HRMS) spectra were recorded by the Imperial College Department of Chemistry Mass Spectrometry Service. X-ray crystal structures were determined using an Agilent Xcalibur 3E diffractometer; for details, see the Supporting Information.

### Procedures for the Synthesis of Molecular Silica Analogues **A2**, **A3**, **A5**, **B1**, **B4**, and **B5**

The appropriate silane (10.0 mmol) was added dropwise to a vigorously stirred solution of triethylamine (2.8 mL, 20.1 mmol) in Et_2_O (30.0 mL) and H_2_O (1.0 mL). After 45 min, the mixture was transferred to a separating funnel containing an aqueous solution of HCl (1.0 M, 21.0 mL, 21.0 mmol). The aqueous layer was extracted with Et_2_O (3 × 10.0 mL), the combined organic portions were washed with brine, dried over MgSO_4_, filtered, and the volatiles were evaporated.

#### *t*-Butyldiphenylsilanol (**A2**)^43^

*t*-Butyldiphenylsilanol was prepared using *t*-butyl(chloro)diphenylsilane (2.6 mL, 10.0 mmol). Then, it was purified by silica plug and eluted with hexane, to remove non-polar components, and then with THF to yield *t*-butyldiphenylsilanol (**A2**) (2.36 g, 9.2 mmol, 92%) as a white solid; mp 59.4–60.3 °C. ATR–FTIR 3256 cm^–1^; ^1^H NMR (400 MHz, CDCl_3_): δ 7.73–7.71 (m, 4H), 7.44–7.36 (m, 6H), 1.88 (br s, 1H), 1.07 (s, 9H); ^13^C{^1^H} NMR (101 MHz, CDCl_3_): δ 135.3, 134.9, 129.8, 127.9, 26.7, 19.2; ^29^Si{^1^H} NMR (80 MHz, CDCl_3_): δ −5.6; HRMS (EI^+^) *m*/*z*: [M]^+•^ calcd for C_16_H_20_OSi, 256.1283; found, 256.1282.

#### Triethylsilanol (**A3**)^44^

Triethylsilanol (**A3**) was prepared using triethylchlorosilane (1.7 mL, 10.0 mmol). Then, it was purified by silica plug and eluted with hexane, to remove non-polar components, and then with Et_2_O to yield triethylsilanol (**A3**) (1.16 g, 8.8 mmol, 88%) as a colorless oil. ATR–FTIR 3293 cm^–1^; ^1^H NMR (400 MHz, CDCl_3_): δ 2.70 (s, 1H), 0.94 (t, *J* = 8.0 Hz, 9H), 0.56 (q, *J* = 8.0 Hz, 6H); ^13^C{^1^H} NMR (101 MHz, CDCl_3_): δ 6.7, 5.9; ^29^Si{^1^H} NMR (80 MHz, CDCl_3_): δ 19.0; HRMS (EI^+^) *m*/*z*: [M]^+•^ calcd for C_6_H_16_OSi, 132.0970; found, 132.0970.

#### Methyldiphenylsilanol (**A5**)^45^

Methyldiphenylsilanol (**A5**) was prepared using chloro(methyl)diphenylsilane (2.1 mL, 10.0 mmol). Then, it was purified by silica plug and eluted with hexane, to remove non-polar components, and then with Et_2_O to yield methyldiphenylsilanol (**A5**) (2.1 g, 9.8 mmol, 98%) as a colorless oil. ATR–FTIR 3278 cm^–1^; ^1^H NMR (400 MHz, CDCl_3_): δ 7.65–7.63 (m, 4H), 7.49–7.45 (m, 2H), 7.43–7.39 (m, 4H), 3.28 (br s, 1H), 0.67 (s, 3H); ^13^C{^1^H} NMR (101 MHz, CDCl_3_): δ 137.1, 134.1, 129.9, 128.0, −1.2; ^29^Si{^1^H} NMR (80 MHz, CDCl_3_): δ −2.8; HRMS (EI^+^) *m*/*z*: [M]^+•^ calcd for C_13_H_14_OSi, 214.0814; found, 214.0813.

#### Di-*i*-propylsilanediol (**B1**)^46^

Di-*i*-propylsilanediol (**B1**) was prepared using diisopropyldichlorosilane (1.8 mL, 10.0 mmol), followed by recrystallization from (hexane/CH_2_Cl_2_, 8:2) to yield di-*i*-propylsilanediol (**B1**) (1.29 g, 8.6 mmol, 86%) as colorless needles; mp 113.2–114.4 °C. ATR–FTIR 3235 cm^–1^; ^1^H NMR (400 MHz, CDCl_3_): δ 2.05 (s, 2H), 0.99 (d, *J* = 6.5 Hz, 12H), 1.02–0.93 (m, 2H); ^13^C{^1^H} NMR (101 MHz, CDCl_3_): δ 17.0, 12.7; ^29^Si{^1^H} NMR (80 MHz, CDCl_3_): δ −3.7; HRMS (EI^+^) *m*/*z*: [M]^+•^ calcd for C_6_H_16_O_2_Si, 148.0920; found, 148.0923.

#### Procedure for the Synthesis of Di(naphthalen-1-yl)silanediol (**B4**)^14a^

To a solution of 1-bromonaphthalene (7.0 mL, 50.0 mmol) in Et_2_O (300 mL) at −78 °C was added *n*-BuLi (2.43 M, 41.15 mL, 100 mmol) at a rate of 3 mL/min. The resulting pale yellow-orange solution was stirred at −78 °C for 1 h, allowed to warm to room temperature and stirred for 2 h. Subsequently, the solution was re-cooled to −78 °C and transferred dropwise by cannula to a solution of silicon tetrachloride (2.86 mL, 25 mmol) in Et_2_O (200 mL) at −78 °C with immediate precipitation of a white solid. After complete transfer, the mixture was allowed to warm to room temperature and stirred for 48 h. The solvent was removed in vacuo to afford a yellow oil containing a white precipitate, and a saturated solution of aqueous NaHCO_3_ was added to neutralize the mixture to pH 7. The mixture was extracted with Et_2_O (3 × 25 mL), the combined organics were washed with brine, dried over MgSO_4_, filtered, and evaporated. Trituration (hexane/Et_2_O, 8:2) afforded di(naphthalen-1-yl)silanediol (**B4**) (6.59 g, 23.3 mmol, 93%) as a white solid; mp 155.2–156.2 °C. ATR–FTIR 3565, 3353 cm^–1^; ^1^H NMR (400 MHz, methanol-*d*_4_): δ 8.41 (d, *J* = 8.3 Hz, 2H), 7.97 (dd, *J* = 6.8, 1.3 Hz, 2H), 7.90 (dd, *J* = 8.3, 1.1 Hz, 2H), 7.82 (dd, *J* = 7.7, 1.8 Hz, 2H), 7.46–7.33 (m, 6H), 4.90 (s, 2H); ^13^C{^1^H} NMR (101 MHz, methanol-*d*_4_): δ 138.4, 136.3, 136.2, 134.8, 131.6, 130.2, 129.6, 126.7, 126.4, 126.0; ^29^Si{^1^H} NMR (80 MHz, methanol-*d*_4_): δ −29.2; HRMS (ES^–^, TOF) *m*/*z*: [M – H]^−^ calcd for C_20_H_15_O_2_Si, 315.0841; found, 315.0837.

#### Procedure for the Synthesis of Di-*t*-butylsilanediol (**B5**)^47^

A solution of di-*t*-butylhyridosilanol (**A1**) (96 mg, 0.6 mmol) in THF (5 mL) was added dropwise over 10 min to an aqueous solution of NaOH (7.7 M, 248 μL, 1.9 mmol) with vigorous stirring and open to air to allow the escape of the hydrogen generated. Once the addition was complete, the mixture was monitored by TLC (hexane/Et_2_O, 9:1) until complete disappearance of the starting silane was observed. The solvent was removed in vacuo, and aqueous HCl solution (1.0 M, 2 mL, 2 mmol) was added to the resulting emulsion. Et_2_O (5 mL) was added, and the aqueous phase was extracted with Et_2_O (2 × 5 mL). The combined organics were washed with brine, dried over MgSO_4_, filtered, and evaporated to yield a white solid. The solid was purified by silica plug and eluted with hexane, to remove non-polar components, and then with Et_2_O to give di-*t*-butylsilanediol (**B5**) (103 mg, 0.58 mmol, 97%) as colorless crystals; mp 151.3–152.3 °C. ATR–FTIR 3293 cm^–1^; ^1^H NMR (400 MHz, CDCl_3_): δ 2.17 (br s, 2H), 1.04 (s, 18H); ^13^C{^1^H} NMR (101 MHz, CDCl_3_): δ 27.4, 19.9; ^29^Si{^1^H} NMR (80 MHz, CDCl_3_): δ −7.1; HRMS (ES^–^, TOF) *m*/*z*: [M – H + HCO_2_H]^−^ calcd for C_9_H_21_O_4_Si, 221.1209; found, 221.1202.

### General Procedure for the Molecular Silica Analogue and Silanol Screening (Figures 1 and 2)

Phenylacetic acid **1a** (136 mg, 1.0 mmol), 4-methylbenzylamine **2a** (130 μL, 1.0 mmol), molecular silicon analogues **A1–5**, **B1–5**, **C1–2**, **D1–2** or triarylsilanols **4**, **6a–k** (0.1 mmol), and toluene (5.0 mL) were placed in a 10 mL round-bottom flask equipped with a stirrer bar. The mixture was purged under a flow of nitrogen for 5 min before bringing to reflux for 1 h. The reaction mixture was allowed to cool, and the solvent was removed in vacuo. CDCl_3_ was added until a homogeneous solution was observed at which point decamethylcyclopentasiloxane (10 μL, 0.0258 mmol) was added as an internal standard. A ^1^H NMR spectrum was recorded, and conversions were calculated by integration against the internal standard. Reactions were conducted in triplicate, and the average conversion is reported. For the use of silanols **4**, **6a–k** catalyst integrity was also determined by integration against the internal standard.

### General Procedures **A1–A3** and **B1–B3** for the Formation of Triarylsilanes and Silanols, Respectively

#### General Procedure **A1**

A solution of aryl bromide (18 mmol) in Et_2_O (10 mL) was added over 5 min to magnesium turnings (437 mg, 18 mmol) that had been vigorously pre-stirred for 1 h. The reaction mixture was stirred as described below, and the resulting solution was cooled to −78 °C and trichlorosilane (0.45 mL, 4.5 mmol) was added. After stirring as described below, the solvent was removed in vacuo and aqueous HCl solution (0.1 M, 10 mL, 1 mmol) was added. The aqueous phase was extracted with Et_2_O (3 × 5 mL), and the combined organics were washed with brine, dried over MgSO_4_, filtered, and evaporated.

#### General Procedure **A2**

To a stirred solution of arylbromide (18 mmol) in Et_2_O (25 mL) was added a solution of *n*-BuLi in hexane (2.27 M, 8.13 mL, 18.5 mmol) at −78 °C. After allowing the reaction mixture to stir at 0 °C for 1 h, then room temperature for 1.5 h, it was cooled to −78 °C, and trichlorosilane (0.45 mL, 4.5 mmol) was added dropwise. The reaction mixture was stirred at −78 °C for 2 h, allowed to warm to room temperature for 2 h, and aqueous HCl solution (0.1 M, 10 mL, 1 mmol) was added. The layers were separated, the aqueous phase was extracted with Et_2_O (3 × 5 mL), and the combined organics were washed with brine, dried over MgSO_4_, filtered, and evaporated.

#### General Procedure **A3**

To a stirred solution of arylbromide (10 mmol) in anhydrous Et_2_O (30 mL) held at −78 °C was added a solution of *i*PrMgCl in THF (1.61 M, 6.06 mL, 10 mmol) over 30 min. After stirring at −78 °C for 30 min, trichlorosilane (0.30 mL, 3 mmol) was added dropwise and the reaction mixture was allowed to warm to room temperature overnight. The precipitate formed was removed by filtration, and the solvent was removed in vacuo.

#### General Procedure **B1**

A solution of the silane in CH_3_CN was added to a stirred solution of [Ru(*p*-cymene)Cl_2_]_2_ (1.8 mg, 0.01 mmol) in CH_3_CN (1 mL) and H_2_O (138 μL). The solution was brought to 80 °C, stirred as detailed below, and the solvent was evaporated.

#### General Procedure **B2**

A solution of silane (2.0 mmol) in THF (10 mL) was added dropwise over 10 min to an aqueous solution of NaOH (7.7 M, 600 μL, 4.62 mmol) while vigorously stirring in the open air to allow the escape of generated H_2_. Once TLC monitoring showed the reaction was complete, the solvent was removed in vacuo and aqueous HCl solution (1.0 M, 5 mL, 5 mmol) was added to the resulting emulsion. Et_2_O (10 mL) was added, the layers were separated, and the aqueous phase was further extracted with Et_2_O (3 × 5 mL). The combined organics were washed with brine, dried over MgSO_4_, filtered, and evaporated.

#### General Procedure **B3**

10 wt % Pd/C (100 mg, 0.1 mmol) and H_2_O (100 μL, 5.5 mmol) were added sequentially to a solution of silane (1.0 mmol) in THF (5 mL) at room temperature with immediate evolution of gas. The mixture was stirred for 45 min, passed through a plug of silica, eluting with CH_2_Cl_2_, and the solvent was evaporated.

#### Tris(4-methoxyphenyl)silane (**5b**)^19b^

Tris(4-methoxyphenyl)silane (**5b**) was prepared according to general procedure **A1** with stirring for 2 h at room temperature for Grignard formation and 4 h at reflux for aryl Grignard addition. Purification by column chromatography (hexane/Et_2_O, 1:1), gave tris(4-methoxyphenyl)silane (**5b**) (400 mg, 1.13 mmol, 25%) as a colorless oil, that over weeks solidified into a white solid; mp 75.2–76.1 °C. ATR–FTIR 2114 cm^–1^; ^1^H NMR (400 MHz, CDCl_3_): δ 7.48 (d, *J* = 8.6 Hz, 6H), 6.92 (d, *J* = 8.6 Hz, 6H), 5.41 (s, ^1^*J*_Si–H_ = 196.5 Hz 1H), 3.82 (s, 9H); ^13^C{^1^H} NMR (101 MHz, CDCl_3_): δ 161.1, 137.4, 125.0, 114.0, 55.2; ^29^Si{^1^H} NMR (80 MHz, CDCl_3_): δ −19.3. A mass spectrum molecular ion could not be observed by electron or chemical ionization techniques.

#### Tri-*p*-tolylsilane (**5c**)^19c^

Tri-*p*-tolylsilane (**5c**) was prepared according to general procedure **A1** with stirring for 5 h at room temperature for Grignard formation and 4 h at reflux for aryl Grignard addition. Purification by column chromatography (hexane) gave tri-*p*-tolylsilane (**5c**) (542 mg, 1.8 mmol, 40%) as a white solid; mp 81.6–83.0 °C. ATR–FTIR 2114 cm^–1^; ^1^H NMR (400 MHz, CDCl_3_): δ 7.51 (d, *J* = 7.7 Hz, 6H), 7.23 (d, *J* = 7.7 Hz, 6H), 5.47 (s, ^1^*J*_Si–H_ = 197.6 Hz, 1H), 2.40 (s, 9H); ^13^C{^1^H} NMR (101 MHz, CDCl_3_): δ 139.8, 135.9, 130.3, 129.0, 21.7; ^29^Si{^1^H} NMR (80 MHz, CDCl_3_): δ −18.6.

#### Tris(4-(*t*-butyl)phenyl)silane (**5d**)^19d^

Tris(4-(*t*-butyl)phenyl)silane (**5d**) was prepared according to general procedure **A2** followed by recrystallization from hexane to give tris(4-(*t*-butyl)phenyl)silane (**5d**) (1.54 g, 3.6 mmol, 80%) as a colorless solid; mp 168.4–169.7 °C. ^1^H NMR (400 MHz, CDCl_3_): δ 7.55–7.52 (m, 6H), 7.41–7.38 (m, 6H), 5.42 (s, ^1^*J*_Si–H_ = 197.1 Hz, 1H), 1.32 (s, 27H); ^13^C{^1^H} NMR (101 MHz, CDCl_3_): δ 152.7, 135.8, 130.4, 125.1, 34.9, 31.4; ^29^Si{^1^H} NMR (80 MHz, CDCl_3_): δ −19.2; HRMS (EI^+^) *m*/*z*: [M]^+•^ calcd for C_30_H_40_Si, 428.2899; found, 428.2916.

#### Tris(4-fluorophenyl)silane (**5e**)^19c^

Tris(4-fluorophenyl)silane (**5e**) was prepared according to general procedure **A1** with stirring at 0 °C, followed by reflux for 1 h to complete Grignard formation and 16 h at room temperature for aryl Grignard addition. Purification by column chromatography (hexane) gave tris(4-fluorophenyl)silane (**5e**) (1.13 g, 3.6 mmol, 80%) as a colorless oil that over time crystallized as colorless crystals; mp 42.3–43.9 °C. ATR–FTIR 2129 cm^–1^; ^1^H NMR (400 MHz, CDCl_3_): δ 7.67–7.62 (m, 6H), 7.21–7.16 (m, 6H), 5.61 (s, ^1^*J*_Si–H_ = 201.8 Hz, 1H); ^13^C{^1^H} NMR (101 MHz, CDCl_3_): δ 164.5 (d, ^1^*J*_C–F_ = 250.3 Hz, *para*), 137.9 (d, ^3^*J*_C–F_ = 7.7 Hz, *ortho*), 128.6 (d, ^4^*J*_C–F_ = 3.6 Hz, *ipso*), 115.7 (d, ^2^*J*_C–F_ = 20.0 Hz, *meta*); ^19^F NMR (377 MHz, CDCl_3_): δ −109.6 (tt, ^3^*J*_F–H_ = 9.2 Hz, ^4^*J*_F–H_ = 5.6 Hz); ^29^Si{^1^H} NMR (80 MHz, CDCl_3_): δ −18.8; HRMS (EI^+^) *m*/*z*: [M – H]^+^ calcd for C_18_H_12_SiF_3_, 313.0660; found, 313.0670.

#### Tris(4-bromophenyl)silane (**5h**)^19f^

Tris(4-bromophenyl)silane (**5h**) was prepared according to general procedure **A2** using 1,4-dibromobenzene (4.72 g, 20.0 mmol), *n*-BuLi (2.5 M in hexane, 8.00 mL, 20.0 mmol), and trichlorosilane (0.61 mL, 6.0 mmol). Recrystallization from hexane gave tris(4-bromophenyl)silane (**5h**) (2.68 g, 5.4 mmol, 90%) as white crystalline needles; mp 109.9–110.5 °C. ^1^H NMR (400 MHz, CDCl_3_): δ 7.53 (d, *J* = 8.2 Hz, 6H), 7.37 (d, *J* = 8.2 Hz, 6H), 5.38 (s, ^1^*J*_Si–H_ = 204.1 Hz, 1H); ^13^C{^1^H} NMR (101 MHz, CDCl_3_): δ 137.3, 131.7, 131.1, 125.5; ^29^Si{^1^H} NMR (80 MHz, CDCl_3_): δ −18.4; HRMS (EI^+^) *m*/*z*: [M]^+•^ calcd for C_18_H_12_^79^Br_3_Si, 492.8258; found, 492.8271.

#### Tris(4-(trifluoromethyl)phenyl)silane (**5j**)^19d^

Tris(4-(trifluoromethyl)phenyl)silane (**5j**) was prepared according to general procedure **A1**, using THF (9 mL) as the reaction solvent, with stirring at room temperature for 1 h followed by reflux for 1 h to complete Grignard formation and 16 h at room temperature for aryl Grignard addition. Purification by column chromatography (hexane) gave tris(4-(trifluoromethyl)phenyl)silane (**5j**) (1.17 g, 2.52 mmol, 56%) as a colorless solid; mp 74.5–75.3 °C. ATR–FTIR 2162 cm^–1^; ^1^H NMR (400 MHz, CDCl_3_): δ 7.71–7.67 (m, 12H), 5.60 (s, ^1^*J*_Si–H_ = 206.5 Hz, 1H); ^13^C{^1^H} NMR (101 MHz, CDCl_3_): δ 136.4, 136.2, 132.7 (q, ^2^*J*_C–F_ = 32.4 Hz), 125.1 (q, ^3^*J*_C–F_ = 3.7 Hz), 124.1 (q, ^1^*J*_C–F_ = 272.5 Hz); ^19^F{^1^H} NMR (377 MHz, CDCl_3_): δ −63.2; ^29^Si{^1^H} NMR (80 MHz, CDCl_3_): δ −18.6; HRMS (EI^+^) *m*/*z*: [M]^+•^ calcd for C_21_H_13_F_9_Si, 464.0643; found, 464.0629.

#### Tris(3,4,5-trifluorophenyl)silane (**5k**)

Tris(3,4,5-trifluorophenyl)silane (**5k**) was prepared according to general procedure **A3**, followed by recrystallization from hexane to give tris(3,4,5-trifluorophenyl)silane (**5k**) (773 mg, 1.83 mmol, 61%) as colorless crystals; mp 80–82 °C. ^1^H NMR (400 MHz, CDCl_3_): δ 7.10–7.06 (app. t, *J*_H–F_ = 6.8 Hz, 6H), 5.39 (s, ^1^*J*_Si–H_ = 215 Hz, 1H); ^13^C{^1^H} NMR (101 MHz, CDCl_3_): δ 151.9 (ddd, ^1^*J*_C–F_ = 256.5 Hz, ^2^*J*_C–F_ = 9.9 Hz, ^3^*J*_C–F_ = 2.6 Hz, *meta*), 141.8 (dt, ^1^*J*_C–F_ = 257.6 Hz, ^2^*J*_C–F_ = 14.9 Hz, *para*), 126.7 (q, *J*_C–F_ = 4.1 Hz, *ipso*), 119.4 (dd, ^2^*J*_C–F_ = 14.3 Hz, ^3^*J*_C–F_ = 5.5 Hz, *ortho*); ^19^F{^1^H} NMR (377 MHz, CDCl_3_): δ −132.3 (d, *J*_F–F_ = 20.5 Hz, *meta*), −155.4 (t, *J*_F–F_ = 20.5 Hz, *para*); ^29^Si{^1^H} NMR (80 MHz, CDCl_3_): δ −16.9; HRMS (EI^+^) *m*/*z*: [M – H]^+^ calcd for C_18_H_6_SiF_9_, 421.0095; found, 421.0103.

#### Tris(4-*N*,*N*-dimethylaminophenyl)silanol (**6a**)^19a^

Tris(4-*N*,*N*-dimethylaminophenyl)silanol (**6a**) was prepared according to general procedure **B1** using silane **5a** (599 mg, 1.54 mmol) in CH_3_CN (2.5 mL) for 24 h. Purification by column chromatography (hexane/EtOAc, 9:1) gave tris-(4-*N*,*N*-dimethylaminophenyl)silanol (**6a**) (475 mg, 1.174 mmol, 76%) as a white solid; mp 183.0–183.7 °C. ^1^H NMR (400 MHz, CDCl_3_): δ 7.54 (d, *J* = 8.7 Hz, 6H), 6.75 (d, *J* = 8.7 Hz, 6H), 2.99 (br s, 18H), 2.39 (s, 1H); ^13^C{^1^H} NMR (101 MHz, CDCl_3_): δ 151.4, 136.4, 122.2, 111.8, 40.3; ^29^Si{^1^H} NMR (80 MHz, CDCl_3_): δ −10.6; HRMS (ES^+^, TOF) *m*/*z*: [M + H]^+^ calcd for C_24_H_32_N_3_OSi, 406.2315; found, 406.2316.

#### Tris(4-methoxyphenyl)silanol (**6b**)

Tris(4-methoxyphenyl)silanol (**6b**) was prepared according to general procedure **B1** using an added solution of silane **5b** (175 mg, 0.5 mmol) in CH_3_CN (1.5 mL) for 5 h. Purification by silica plug, followed by eluting with hexane to remove non-polar components and then with CH_2_Cl_2_ gave tris(4-methoxyphenyl)silanol (**6b**) (124 mg, 0.375 mmol, 71%) as a colorless oil. ATR–FTIR 3349 cm^–1^; ^1^H NMR (400 MHz, CDCl_3_): δ 7.54 (d, *J* = 8.5 Hz, 6H), 6.92 (d, *J* = 8.5 Hz, 6H), 3.82 (s, 9H), 2.38 (s, 1H); ^13^C{^1^H} NMR (101 MHz, CDCl_3_): δ 161.3, 136.7, 127.0, 113.8, 55.2; ^29^Si{^1^H} NMR (80 MHz, CDCl_3_): δ −11.7; HRMS (ES^–^, TOF) *m*/*z*: [M – H]^−^ calcd for C_21_H_21_O_4_Si, 365.1209; found, 365.1213.

#### Tris-*p*-tolylsilanol (**6c**)^21b^

Tris-*p*-tolylsilanol (**6c**) was prepared according to general procedure **B2** using silane **5c** on a 1.5 mmol scale. Purification by silica plug, followed by eluting with hexane, to remove non-polar components, and then with Et_2_O gave tri-*p*-tolylsilanol (**6c**) (449 mg, 1.41 mmol, 94%) as a white solid; mp 100.7–101.2 °C. ^1^H NMR (400 MHz, CDCl_3_): δ 7.54 (d, *J* = 7.7 Hz, 6H), 7.22 (d, *J* = 7.7 Hz, 6H), 2.58 (s, 1H), 2.40 (s, 9H); ^13^C{^1^H} NMR (101 MHz, CDCl_3_): δ 140.1, 135.2, 132.1, 128.8, 21.7; ^29^Si{^1^H} NMR (80 MHz, CDCl_3_): δ −11.8; HRMS (EI^+^) *m*/*z*: [M]^+•^ calcd for C_21_H_22_OSi, 318.1440; found, 318.1447.

#### Tris(4-(*t*-butyl)phenyl)silanol (**6d**)

Tris(4-(*t*-butyl)phenyl)silanol (**6d**) was prepared according to general procedure **B3** using silane **5d** to give tris(4-(*t*-butyl)phenyl)silanol (**6d**) (444 mg, 1.0 mmol, 100%) as a white solid; mp 226–227 °C. ATR–FTIR 3662 cm^–1^; ^1^H NMR (400 MHz, CDCl_3_): δ 7.60 (d, *J* = 8.3 Hz, 6H), 7.41 (d, *J* = 8.3 Hz, 6H), 2.39 (s, 1H), 1.33 (s, 27H); ^13^C{^1^H} NMR (101 MHz, CDCl_3_): δ 153.1, 135.0, 132.2, 125.0, 34.9, 31.4; ^29^Si{^1^H} NMR (80 MHz, CDCl_3_): δ −12.4; HRMS (APCI^–^) *m*/*z*: [M – H]^−^ calcd for C_30_H_39_OSi, 443.2765; found, 443.2753.

#### Tris(4-fluorophenyl)silanol (**6e**)^21c^

Tris(4-fluorophenyl)silanol (**6e**) was prepared according to general procedure **B2** using silane **5e** on a 0.6 mmol scale. Purification by silica plug, followed by eluting with hexane, to remove non-polar components, and then with Et_2_O gave tris(4-fluorophenyl)silanol (**6e**) (190 mg, 0.58 mmol, 96%) as a white solid; mp 88.2–89.7 °C. ATR–FTIR 3207 cm^–1^; ^1^H NMR (400 MHz, CDCl_3_): δ 7.56 (ddt, *J* = 8.4, 6.1, 2.3 Hz, 6H), 7.12–7.07 (m, 6H), 2.72 (s, 1H); ^13^C{^1^H} NMR (101 MHz, CDCl_3_): δ 164.6 (d, ^1^*J*_C–F_ = 250.6 Hz, *para*), 137.2 (d, ^3^*J*_C–F_ = 7.8 Hz, *ortho*), 130.5 (d, ^4^*J*_C–F_ = 4.3 Hz, *ipso*), 115.5 (d, ^2^*J*_C–F_ = 19.9 Hz, *meta*); ^19^F{^1^H} NMR (377 MHz, CDCl_3_): δ −109.4; ^29^Si{^1^H} NMR (80 MHz, CDCl_3_): δ −12.86; HRMS (ES^–^, TOF) *m*/*z*: [M – H]^−^ calcd for C_18_H_12_OSiF_3_, 329.0610; found, 329.0613.

#### Tris(4-chlorophenyl)silanol (**6f**)^21d^

Tris(4-chlorophenyl)silanol (**6f**) was prepared according to general procedure **B2** using silane **5f**. Purification by silica plug and eluting with hexane, removing non-polar components, and then Et_2_O gave tris(4-chlorophenyl)silanol (**6f**) (713 mg, 1.88 mmol, 94%) as a white solid; mp 119.9–120.7 °C. ATR–FTIR 3144 cm^–1^; ^1^H NMR (400 MHz, CDCl_3_): δ 7.51–7.48 (m, 6H), 7.40–7.37 (m, 6H), 2.64 (s, 1H); ^13^C{^1^H} NMR (101 MHz, CDCl_3_): δ 137.2, 136.3, 132.6, 128.6; ^29^Si{^1^H} NMR (80 MHz, CDCl_3_): δ −12.9.

#### Tris(3-chlorophenyl)silanol (**6g**)

Tris(3-chlorophenyl)silanol (**6g**) was prepared according to general procedure **B2** using silane **5g** on a 1.9 mmol scale. Purification by silica plug, followed by eluting with hexane, to remove non-polar components, and then with Et_2_O gave tris(3-chlorophenyl)silanol (**6g**) (728 mg, 1.9 mmol, 100%) as a colorless oil. ATR–FTIR 3262 cm^–1^; ^1^H NMR (400 MHz, CDCl_3_): δ 7.55–7.54 (m, 3H), 7.46–7.42 (m, 6H), 7.35–7.31 (m, 3H), 3.34 (s, 1H); ^13^C{^1^H} NMR (101 MHz, CDCl_3_): δ 136.4, 134.8, 134.6, 132.9, 130.9, 129.8; ^29^Si{^1^H} NMR (80 MHz, CDCl_3_): δ −14.6; HRMS (EI^+^) *m*/*z*: [M]^+•^ calcd for C_18_H_13_OSi^35^Cl_3_, 377.9801; found, 377.9816.

#### Tris(4-bromophenyl)silanol (**6h**)^21c^

Tris(4-bromophenyl)silanol (**6h**) was prepared according to general procedure **B2** using silane **5h**. Purification by silica plug, followed by eluting with hexane, to remove non-polar components, and then with Et_2_O gave tris(4-bromophenyl)silanol (**6h**) (995 mg, 1.9 mmol, 97%) as a white solid; mp 125.9–126.5 °C. ATR–FTIR 3155 cm^–1^; ^1^H NMR (400 MHz, CDCl_3_): δ 7.54 (d, *J* = 8.4 Hz, 6H), 7.42 (d, *J* = 8.4 Hz, 6H), 2.56 (s, 1H); ^13^C{^1^H} NMR (101 MHz, CDCl_3_): δ 136.5, 133.0, 131.5, 125.9; ^29^Si{^1^H} NMR (80 MHz, CDCl_3_): δ −12.6; HRMS (ES^–^, TOF) *m*/*z*: [M – H]^−^ calcd for C_18_H_12_OSi^79^Br_3_, 508.8208; found, 508.8219.

#### Tris(3-(trifluoromethyl)phenyl)silanol (**6i**)

Tris(3-(trifluoromethyl)phenyl)silanol (**6i**) was prepared according to general procedure **B2** using silane **5i** on a 0.6 mmol scale. Purification by silica plug, followed by eluting with hexane, to remove non-polar components, and then with Et_2_O gave tris(3-(trifluoromethyl)phenyl)silanol (**6i**) (273 mg, 0.57 mmol, 95%) as a white solid; mp 82–83 °C. ATR–FTIR 3202 cm^–1^; ^1^H NMR (400 MHz, CDCl_3_): δ 7.89–7.88 (m, 3H), 7.77–7.74 (m, 6H), 7.58–7.54 (m, 3H), 2.83 (s, 1H); ^13^C{^1^H} NMR (101 MHz, CDCl_3_): δ 138.3, 135.0, 131.3 (q, *J* = 3.6 Hz), 131.0 (q, *J* = 32.1 Hz), 128.8, 127.7 (q, *J* = 3.7 Hz), 124.2 (q, *J* = 272.5 Hz); ^19^F{^1^H} NMR (377 MHz, CDCl_3_): δ −62.9; ^29^Si{^1^H} NMR (80 MHz, CDCl_3_): δ −14.6; HRMS (ES^–^, TOF) *m*/*z*: [M – H]^−^ calcd for C_21_H_12_OF_9_Si, 479.0514; found, 479.0506.

#### Tris(4-(trifluoromethyl)phenyl)silanol (**6j**)

Tris(4-(trifluoromethyl)phenyl)silanol (**6j**) was prepared according to general procedure **B2** using silane **5j** on a 1.5 mmol scale. Purification by silica plug, followed by eluting with hexane, to remove non-polar components, and then with Et_2_O gave tris(4-(trifluoromethyl)phenyl)silanol (**6j**) (595 mg, 1.24 mmol, 85%) as a white solid; mp 101.8–102.9 °C. ATR–FTIR 3194 cm^–1^; ^1^H NMR (400 MHz, CDCl_3_): δ 7.74 (d, *J* = 7.8 Hz, 6H), 7.68 (d, *J* = 7.8 Hz, 6H), 3.19 (s. 1H); ^13^C{^1^H} NMR (101 MHz, CDCl_3_): δ 138.3, 135.3, 132.8 (q, *J* = 32.3 Hz), 125.0 (q, *J* = 3.8 Hz), 124.0 (q, *J* = 272.5 Hz); ^19^F{^1^H} NMR (377 MHz, CDCl_3_): δ −63.2; ^29^Si{^1^H} NMR (79 MHz, CDCl_3_): δ −14.5; HRMS (ES^–^, TOF) *m*/*z*: [M – H]^−^ calcd for C_21_H_12_OSiF_9_, 479.0514; found, 479.0504.

#### Tris(3,4,5-trifluorophenyl)silanol (**6k**)

Tris(3,4,5-trifluorophenyl)silanol (**6k**) was prepared according to general procedure **B2** using silane **5k** on a 0.6 mmol scale to give tris-(3,4,5-trifluorophenyl)silanol (**6k**) (269 mg, 0.6 mmol, 99%) as a white solid; mp 266–268 °C. ATR–FTIR 3222 cm^–1^; ^1^H NMR (400 MHz, CDCl_3_): δ 7.14 (app. t, *J*_H–F_ = 6.9 Hz, 6H), 2.98 (s, 1H); ^13^C{^1^H} NMR (101 MHz, CDCl_3_): δ 151.8 (ddd, ^1^*J*_C–F_ = 256.8 Hz, ^2^*J*_C–F_ = 10.4 Hz, ^3^*J*_C–F_ = 2.6 Hz, *meta*), 142.0 (dt, ^1^*J*_C–F_ = 257.5 Hz, ^2^*J*_C–F_ = 15.1 Hz, *para*), 127.8 (q, *J* = 3.4 Hz, *ipso*), 118.7 (dd, ^2^*J*_C–F_ = 13.9 Hz, ^3^*J*_C–F_ = 5.4 Hz, *ortho*); ^19^F NMR (377 MHz, CDCl_3_): δ −132.4 (dd, ^3^*J*_F–F_ = 20.0, ^3^*J*_F–H_ = 6.3 Hz, *meta*), −155.2 (tt, ^3^*J*_F–F_ = 20.0, ^4^*J*_F–H_ = 6.9 Hz, *para*); ^29^Si{^1^H} NMR (80 MHz, CDCl_3_): δ −15.9; HRMS (ES^–^, TOF) *m*/*z*: [M – H]^−^ calcd for C_18_H_6_OSiF_9_, 437.0044; found, 437.0032.

### General Procedure for Conversion versus Time Plots with Catalytic Quantities of Silanols **4**, **6e–f**, and **6h** (Figure 4)

Phenylacetic acid (0.41 g, 3.0 mmol), 4-methylbenzylamine (0.38 mL, 3.0 mmol), triarylsilanols **4**, **6e**, **6f**, **6h** (10 or 30 mol %), tetrakis(trimethylsilyl)methane (30 mg, 0.098 mmol) as internal standard, and toluene (15 mL) were placed within a 100 mL two neck round-bottom flask. The mixture was purged under a flow of nitrogen for 5 min before bringing to reflux. After the elapsed time, an aliquot from the reaction mixture was taken and transferred to a glass vial. Chloroform-*d* was added until a homogeneous solution was observed at which point the ^1^H NMR spectra were recorded. Conversions were calculated by integration against the internal standard. Reactions were conducted in triplicate, and the average conversions are reported.

### General Procedure for Silanol **6h** Catalyzed Preparation of Amides (Figure 5)

Carboxylic acid (5.0 mmol), amine (5.0 mmol), tris(4-bromophenyl)silanol **6h** (0.76 g, 1.5 mmol, 30 mol %), and toluene (25 mL) were charged into a 1000 mL round-bottom flask. This mixture was purged under a flow of nitrogen for 5 min after which the mixture was heated to reflux for 24 h. The solution was then allowed to cool and concentrated in vacuo. The residue was diluted with EtOAc (20 mL), and the resulting solution was washed with aqueous NaOH (25 mL, 0.4 M) and aqueous HCl (25 mL, 1.0 M) solutions. The organic layer was dried over MgSO_4_, filtered, and evaporated. The residue was purified by dry column vacuum chromatography (2–8% EtOAc in petroleum ether) to yield amide products.

#### *N*-(4-Methylbenzyl)-2-phenylacetamide (**3a**)^10j^

Following the general procedure, the product precipitated from the reaction upon cooling. Collection by filtration and washing with toluene gave amide **3a** (0.72 g, 100%) as a white solid; mp 139.0–140.0 °C (lit.^10j^ mp 139.0–139.3 °C); ^1^H NMR (400 MHz, CDCl_3_): δ 7.37–7.32 (m, 2H), 7.31–7.27 (m, 3H), 7.11–7.06 (m, 4H), 5.64 (br s, 1H), 4.37 (d, *J* = 5.7 Hz, 2H), 3.63 (s, 2H), 2.31 (s, 3H); ^13^C{^1^H} NMR (101 MHz, CDCl_3_): δ 170.9, 137.2, 135.1, 134.9, 129.6, 129.4, 129.1, 127.6, 127.5, 44.0, 43.5, 21.2.

#### 1-Morpholino-2-phenylethan-1-one (**3b**)^10j^

Following the general procedure gave amide **3b** (0.21 g, 1.0 mmol, 20%) as a pale yellow crystalline solid; mp 65.8–66.8 °C (lit.^10j^ mp 65–67 °C); ^1^H NMR (400 MHz, CDCl_3_): δ 7.37–7.34 (m, 2H), 7.30–7.27 (m, 3H), 3.76 (s, 2H), 3.67 (s, 4H), 3.52–3.49 (m, 2H), 3.47–3.45 (m, 2H); ^13^C{^1^H} NMR (101 MHz, CDCl_3_): δ 169.7, 134.8, 128.8, 128.6, 126.9, 66.8, 66.5, 46.6, 42.2, 40.9.

#### *N*-Benzyl-*N*-methyl-2-phenylacetamide (**3c**)^10j^

Following the general procedure gave amide **3c** (0.12 g, 0.5 mmol, 10%) as a colorless oil; ^1^H NMR (400 MHz, CDCl_3_): δ major rotamer 7.40–7.10 (m, 10H), 4.65 (s, 2H), 3.82 (s, 2H), 2.93 (s, 3H); minor rotamer 7.40–7.10 (m, 10H), 4.56 (s, 2H), 3.79 (s, 2H), 2.99 (s, 3H); ^13^C{^1^H} NMR (101 MHz, CDCl_3_): δ 171.5, 171.1, 137.3, 136.5, 135.1, 134.9, 128.9, 128.8, 128.7, 128.6, 128.0, 127.6, 127.3, 126.8, 126.4, 53.6, 50.9, 41.2, 40.9, 35.2, 34.0.

#### *N*,2-Diphenylacetamide (**3d**)^10j^

Following the general procedure followed by trituration with *n*-hexane gave amide **3d** (0.35 g, 1.6 mmol, 30%) as a white solid; mp 113.4–114.8 °C (lit.^10j^ mp 113–115 °C); ^1^H NMR (400 MHz, CDCl_3_): δ 7.43–7.39 (m, 4H), 7.36–7.33 (m, 3H), 7.30–7.28 (m, 2H), 7.12 (br s, 1H), 7.07 (t, *J* = 7.4 Hz, 1H), 3.74 (s, 2H); ^13^C{^1^H} NMR (101 MHz, CDCl_3_): δ 169.2, 137.7, 134.6, 129.6, 129.4, 129.0, 127.8, 124.6, 119.9, 45.0.

#### *N*-(4-Methylbenzyl)benzamide (**3e**)^10j^

Following the general procedure gave amide **13** (90 mg, 0.4 mmol, 8%) as a white solid; mp 140.3–141 °C (lit.^10j^ mp 140–141 °C); ^1^H NMR (400 MHz, CDCl_3_): δ 7.80 (dd, *J* = 7.1, 1.7 Hz, 2H), 7.55–7.49 (m, 1H), 7.48–7.41 (m, 2H), 7.30–7.25 (m, 2H), 7.19 (d, *J* = 7.8 Hz, 2H), 6.40 (br s, 1H), 4.63 (d, *J* = 5.6 Hz, 2H), 2.37 (s, 3H); ^13^C{^1^H} NMR (101 MHz, CDCl_3_): δ 167.4, 137.5, 135.3, 134.6, 131.5, 129.5, 128.6, 128.0, 127.1, 44.0, 21.2.

### General Procedure for Silanol **6h** Catalyzed Concentration versus Time Plots (Figures 6 and 7)

Phenylacetic acid (0.41g, 3.0 mmol, 1 equiv), 4-methylbenzylamine (0.38 mL, 3.0 mmol, 1 equiv), tris(4-bromophenyl)silanol **6h** (0.46 g, 0.9 mmol, 30 mol %), tetrakis(trimethylsilyl)methane (30 mg, 0.098 mmol) as internal standard, and toluene (15 mL, 0.2 M) were charged into a 100 mL two-neck round-bottomed flask. *N*-(4-Methylbenzyl)-2-phenylacetamide **3a** (0.36 g, 1.5 mmol, 0.1 M) and/or H_2_O (27 μL, 1.5 mmol, 0.1 M) or amides **3b–d** (1.5 mmol, 0.1 M) were added, and the mixture was purged under a flow of N_2_ for 5 min before bringing to reflux. After the elapsed time, an aliquot from the reaction mixture was taken and transferred to a glass vial. Chloroform-*d* was added until a homogeneous solution was observed at which point ^1^H NMR spectra were recorded. Concentrations were calculated by integration against the internal standard. Reactions were conducted in triplicate, and the average concentrations are reported.

### General Procedure for Determining Catalyst **6h** Integrity (Figure 8)

Phenylacetic acid (0.41 g, 3.0 mmol, 1 equiv), amine (3.0 mmol, 1 equiv), tris(4-bromophenyl)silanol **6h** (0.46 g, 0.9 mmol, 30 mol %), durene (30 mg, 0.2 mmol) as internal standard, and toluene (15 mL, 0.2 M) were charged into a 100 mL two-neck round-bottom flask. The mixture was purged under a flow of N_2_ for 5 min before bringing to reflux for the required time. After the elapsed time, an aliquot (100 μL) was taken and added to *i*PrOH (1000 μL) to give a homogeneous solution. Samples were analyzed on a reverse phase 25 cm × 4.6 mm, 5 μm SUPELCOSIL LC-18 column @ 222/230 nm, 80:20 MeCN/H_2_O, flow rate 2.0 mL/min. Catalyst integrity was determined by integration against the internal standard and by reference to a pre-determined calibration curve.

#### Triphenylsilyl 2-Phenylacetate **7**

A stirred solution of triphenylsilane (2.63 g, 10.1 mmol), phenylacetic acid (1.37 g, 10.1 mmol), and [Ru(*p*-cymene)Cl_2_]_2_ (62 mg, 0.10 mmol) in toluene (15 mL) was held at 50 °C for 18 h. The solution turned from pale orange to deep red/brown over this time. The solvent was evaporated, and the solid residue was dissolved in Et_2_O (50 mL) and passed through a column of dry activated carbon. The solvent was evaporated, and recrystallization from hexane gave triphenylsilyl 2-phenylacetate (**7**) (3.18 g, 8.08 mmol, 80%) as colorless blocks; mp 91.2–92.0 °C. ^1^H NMR (400 MHz, CDCl_3_): δ 7.61–7.59 (m, 6H), 7.49–7.44 (m, 3H), 7.39–7.36 (m, 6H), 7.34–7.29 (m, 5H), 3.79 (s, 2H); ^13^C{^1^H} NMR (101 MHz, CDCl_3_): δ 171.2, 135.8, 134.2, 132.1, 130.6, 129.6, 128.7, 128.0, 127.2, 43.3; ^29^Si{^1^H} NMR (80 MHz, CDCl_3_): δ −9.9; HRMS (EI^+^) *m*/*z*: [M]^+•^ calcd for C_26_H_22_O_2_Si, 394.1389; found, 394.1395.

#### *N*-(4-Methylbenzyl)(triphenylsilyl)amine **9**

To a solution 4-methylbenzylamine (113 μL, 0.87 mmol) in hexane (10 mL) at −78 °C was added dropwise a solution of *n*-BuLi in hexane (2.35 M, 370 μL, 0.87 mL) over a 5 min period to form a dull pink solution. Triphenylchlorosilane (258 mg, 0.87 mmol) as a solution in Et_2_O (10 mL) was added dropwise, forming a pale-yellow solution with a white precipitate. The reaction mixture was stirred for 2 h at room temperature, the solvent was evaporated, and the resulting solid was triturated with boiling hexane. The product crystallized from the triturate to give *N*-(4-methylbenzyl)(triphenylsilyl)amine (**9**) (263 mg, 0.70 mmol, 80%) as a colorless crystalline solid; mp 85 °C. ^1^H NMR (400 MHz, CDCl_3_): δ 7.71–7.68 (m, 6H), 7.48–7.44 (m, 3H), 7.43–7.39 (m, 6H), 7.23 (d, *J* = 7.8 Hz, 2H), 7.14 (d, *J* = 7.8 Hz, 2H), 4.11 (d, *J* = 7.5 Hz, 2H), 2.37 (s, 3H), 1.55 (t, *J* = 7.5 Hz, 1H); ^13^C NMR (101 MHz, CDCl_3_): δ 140.6, 136.2, 135.7, 135.1, 129.8, 129.1, 128.0, 127.2, 46.5, 21.2; ^29^Si NMR (80 MHz, CDCl_3_): δ −16.0; HRMS (EI^+^) *m*/*z*: [M]^+•^ calcd for C_26_H_25_NSi, 379.1756; found, 379.1760.

## Data Availability

The data underlying this study are available in the published article and its online Supporting Information as well as openly available as part of the PhD thesis of B. C. Rowley in the Imperial College London Institutional Repository (Spiral) at https://doi.org/10.25560/78224. Computational data are openly available at the Imperial College research data repository at DOI: 10.14469/hpc/12480.

## Supporting Information Available

Copies of NMR spectra for new compounds, catalyst integrity and VTNA plots, and X-ray crystallographic data (PDF)
