# Supplementary material for: On the Use of Triarylsilanols as Catalysts for Direct Amidation of Carboxylic Acids
Source: J Org Chem. 2023 Jul 11;88(14):9853–69. doi: 10.1021/acs.joc.3c00585 (PMC10367078; doi:10.1021/acs.joc.3c00585)
Supplement: Supplementary file 1 — jo3c00585_si_001.pdf [file jo3c00585_si_001.pdf]

ELECTRONIC SUPPORTING INFORMATION

On the use of Triarylsilanols as Catalysts for Direct Amidation of Carboxylic Acids

D. Christopher Braddock,*[a] Ben C. Rowley,[a] Paul D. Lickiss,*[a] Steven J. Fussell,[b] Rabia Qamar,[a] David Pugh,[c] Henry S. Rzepa,[a] and Andrew J. P. White[a]

[a]*Department of Chemistry, Imperial College London, MSRH, White City Campus, 82 Wood Lane, London, W12 0BZ, UK.* [b]*Pfizer Ltd, Chemical R&D, Sandwich, CT13 9NJ, UK.* [c]*Department of Chemistry, King's College London, Britannia House, 7 Trinity Street, London, SE1 1DB, UK.*

Email Address: c.braddock@imperial.ac.uk, p.lickiss@imperial.ac.uk

Cover Page and Contents

pESI 2-34	Copies of ^{1}H, ^{13}C{^{1}H}, ^{19}F/^{19}F{^{1}H}, ^{29}Si{^{1}H} & ^{1}H-^{29}Si HMBC NMR spectra as appropriate for new compounds **5k**, **6b**, **6d**, **6g**, **6i-k**, and **9** and for known (but previously incompletely characterized) **7**
pESI 35	Catalyst integrity plots of [**6h**] vs time in refluxing toluene
pESI 36	VTNA plots of concentration vs normalised timescale to determine order in acid and amine
pESI 36	VTNA plots of concentration vs normalised timescale to determine order in catalyst
pESI 37-46	X-Ray Crystallographic Data for silanes **5e**, **5f**, silanols **6d**, **6e**, **6f**, **6h**, **6k**, disiloxanes of **6e**, **6h** & **6i**, and **7**, **9**, **11** and **12**
pESI 46	References
pESI 47-55	Crystal structure Figures

Copies of ^{1}H, ^{13}C, ^{29}Si NMR spectra, IR spectra and HRMS for all compounds are available open access as part of the PhD thesis of B. C. Rowley in the Imperial College London Institutional Repository (Spiral) at https://doi.org/10.25560/78224.

^{1}H NMR spectrum of tris(3,4,5-trifluorophenyl)silane (5k) (400 MHz, $CDCl_3$)

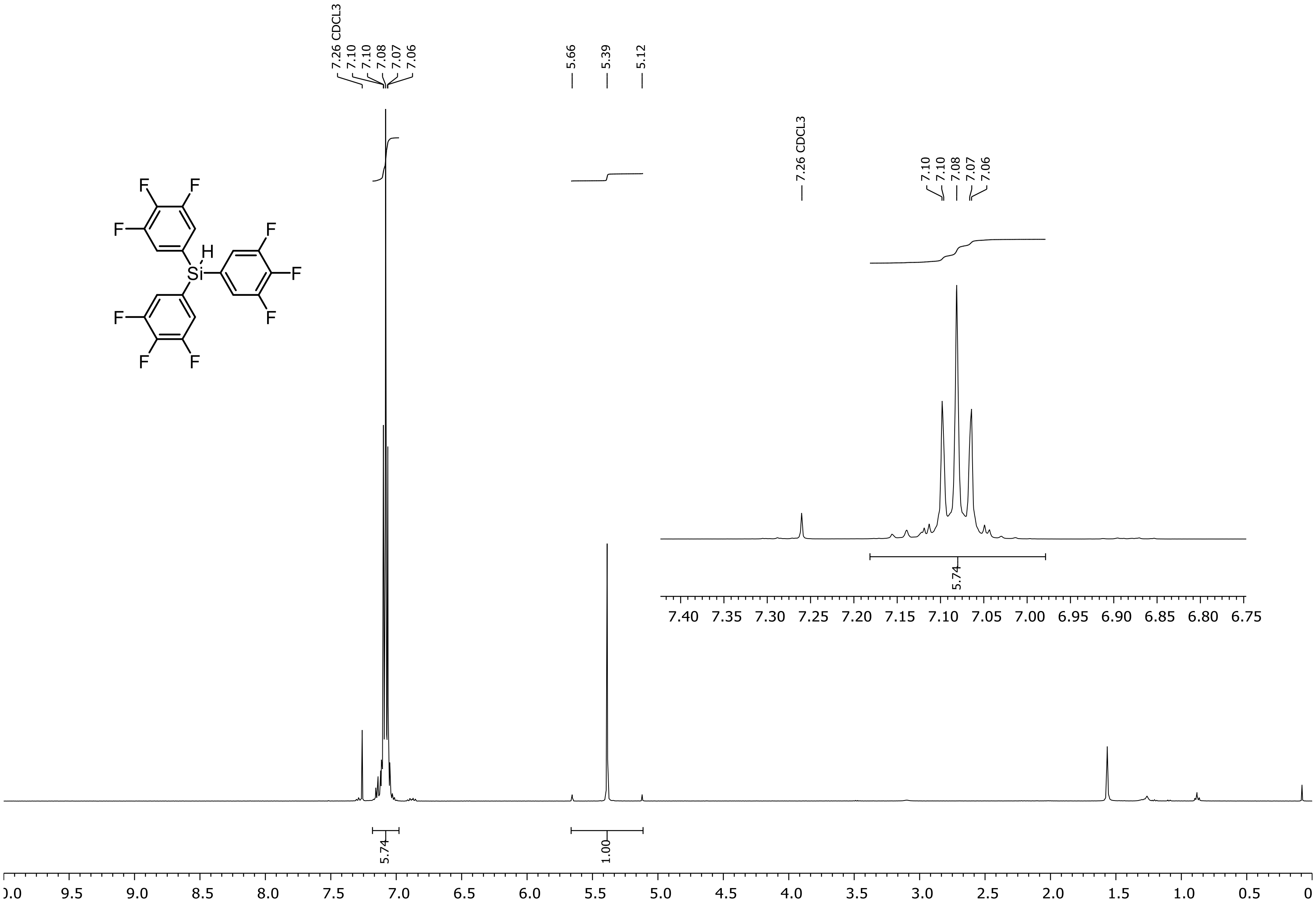

$^{13}C\{^1H\}$ NMR spectrum of tris(3,4,5-trifluorophenyl)silane (5k) (101 MHz, $CDCl_3$)

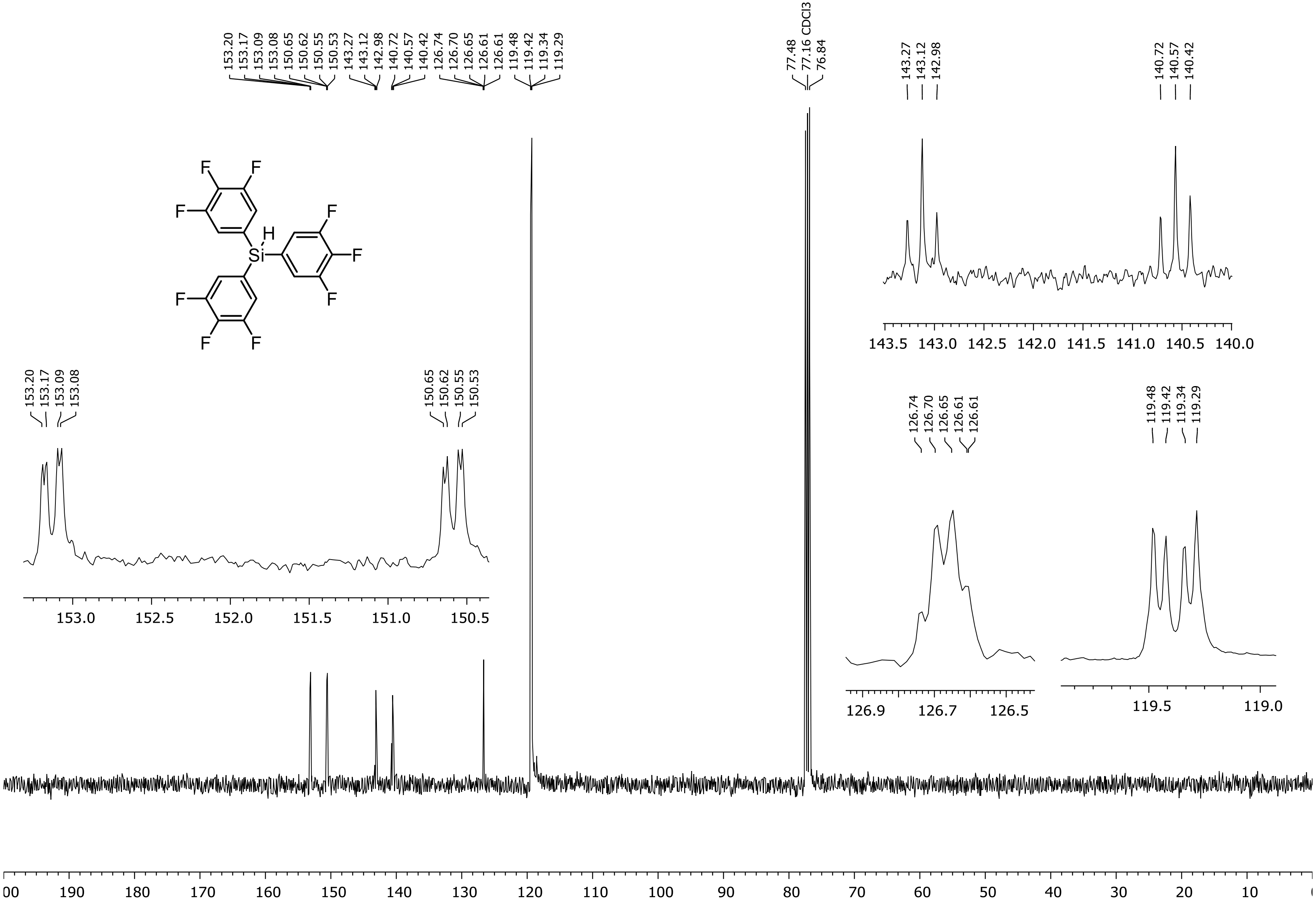

$^{19}F\{^{1}H\}$ NMR spectrum of tris(3,4,5-trifluorophenyl)silane (5k) (377 MHz, $CDCl_3$)

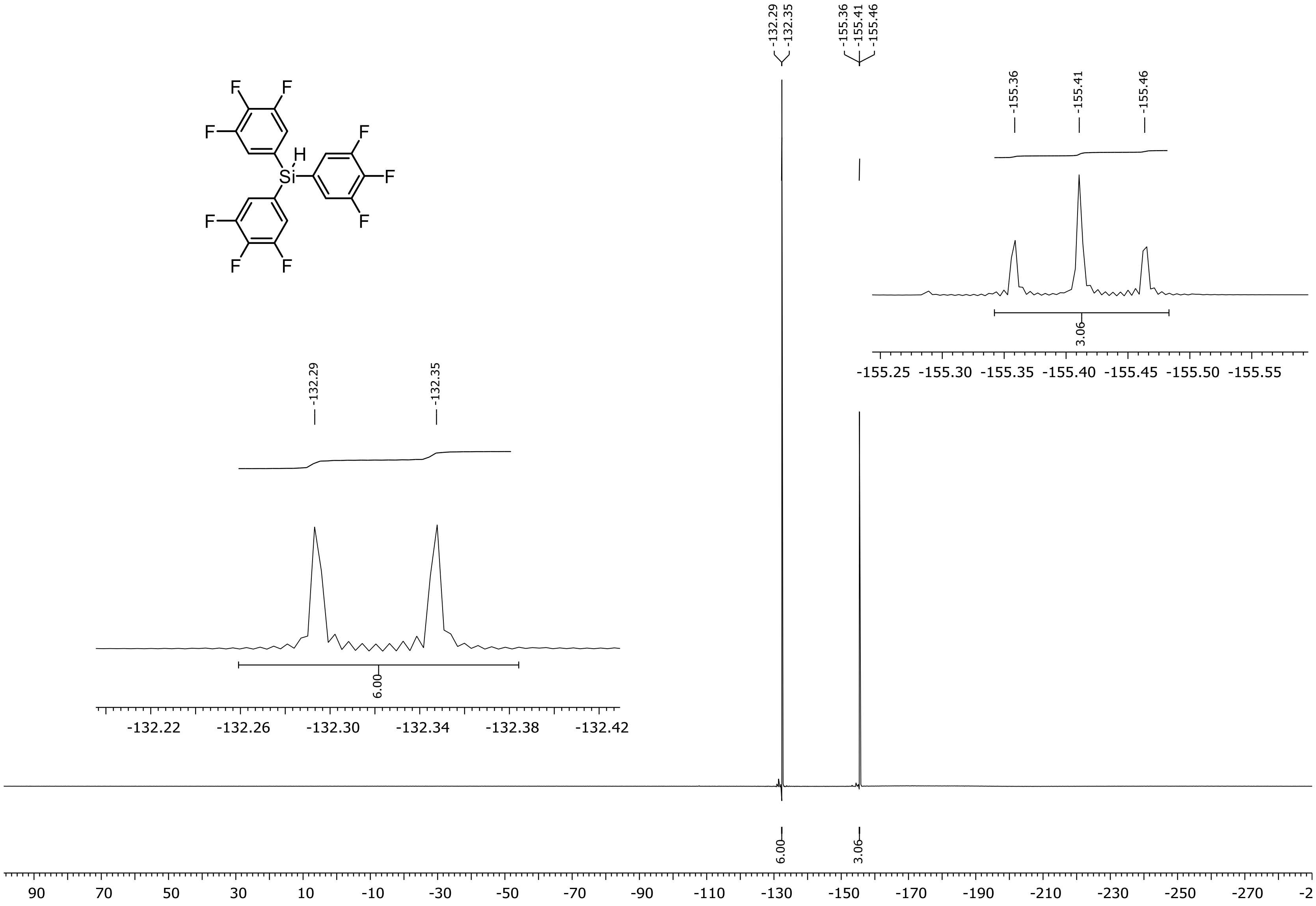

$^{29}Si\{^{1}H\}$ NMR spectrum of tris(3,4,5-trifluorophenyl)silane (5k) (80 MHz, $CDCl_3$)

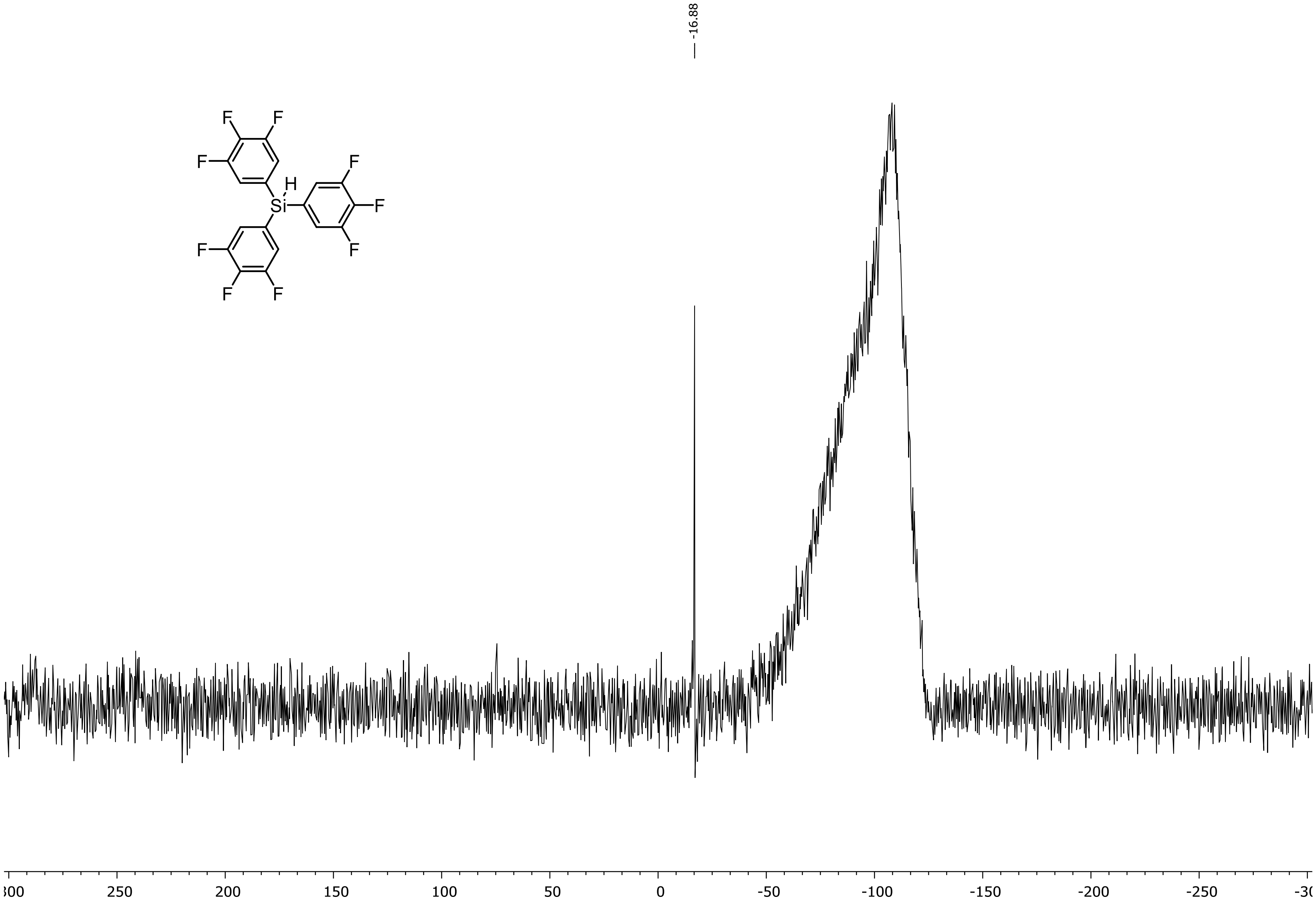

^{1}H NMR spectrum of tris(4-methoxyphenyl)silanol (6b) (400 MHz, $CDCl_3$)

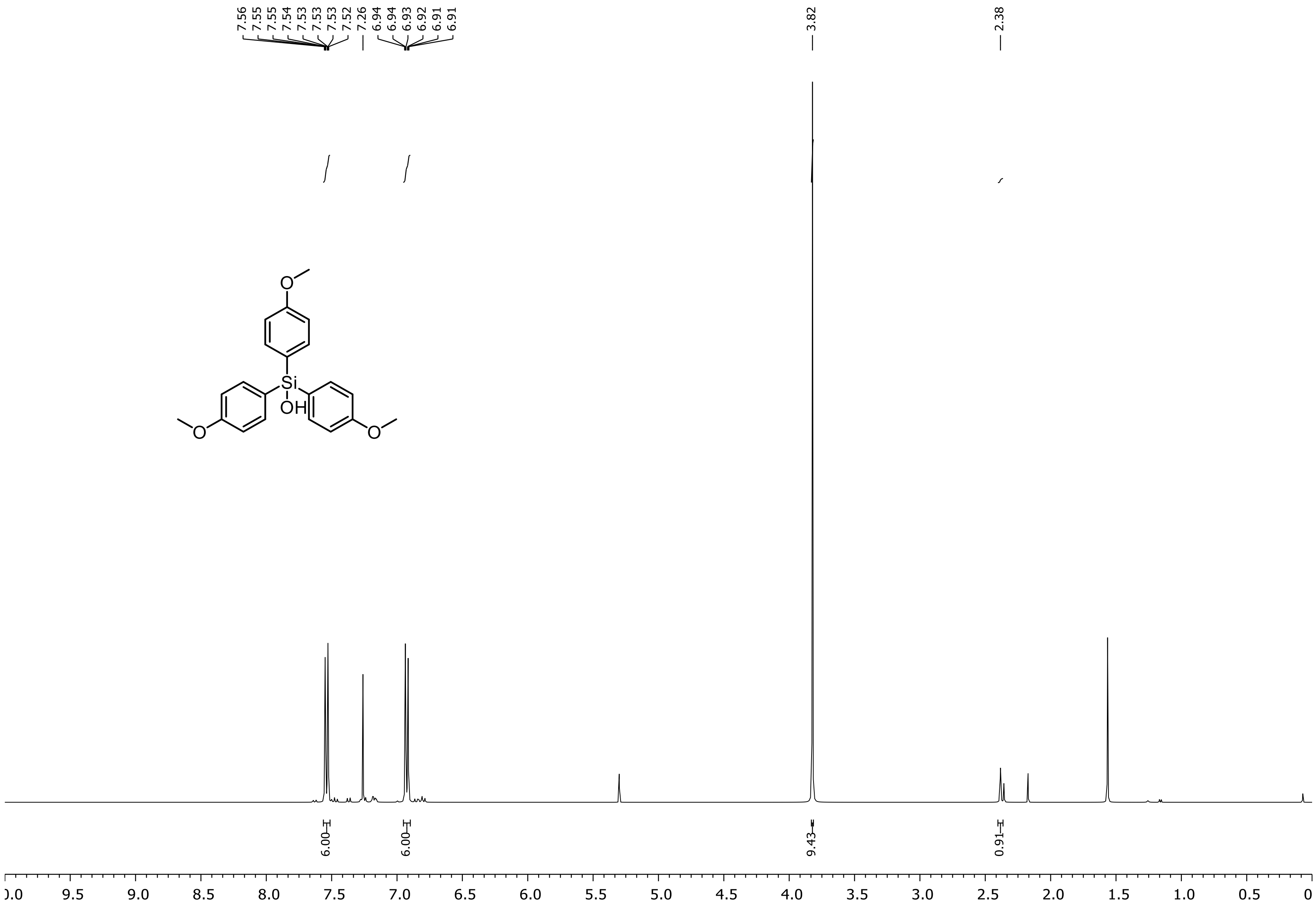

$^{13}C\{^1H\}$ NMR spectrum tris(4-methoxyphenyl)silanol (6b) (101 MHz, $CDCl_3$)

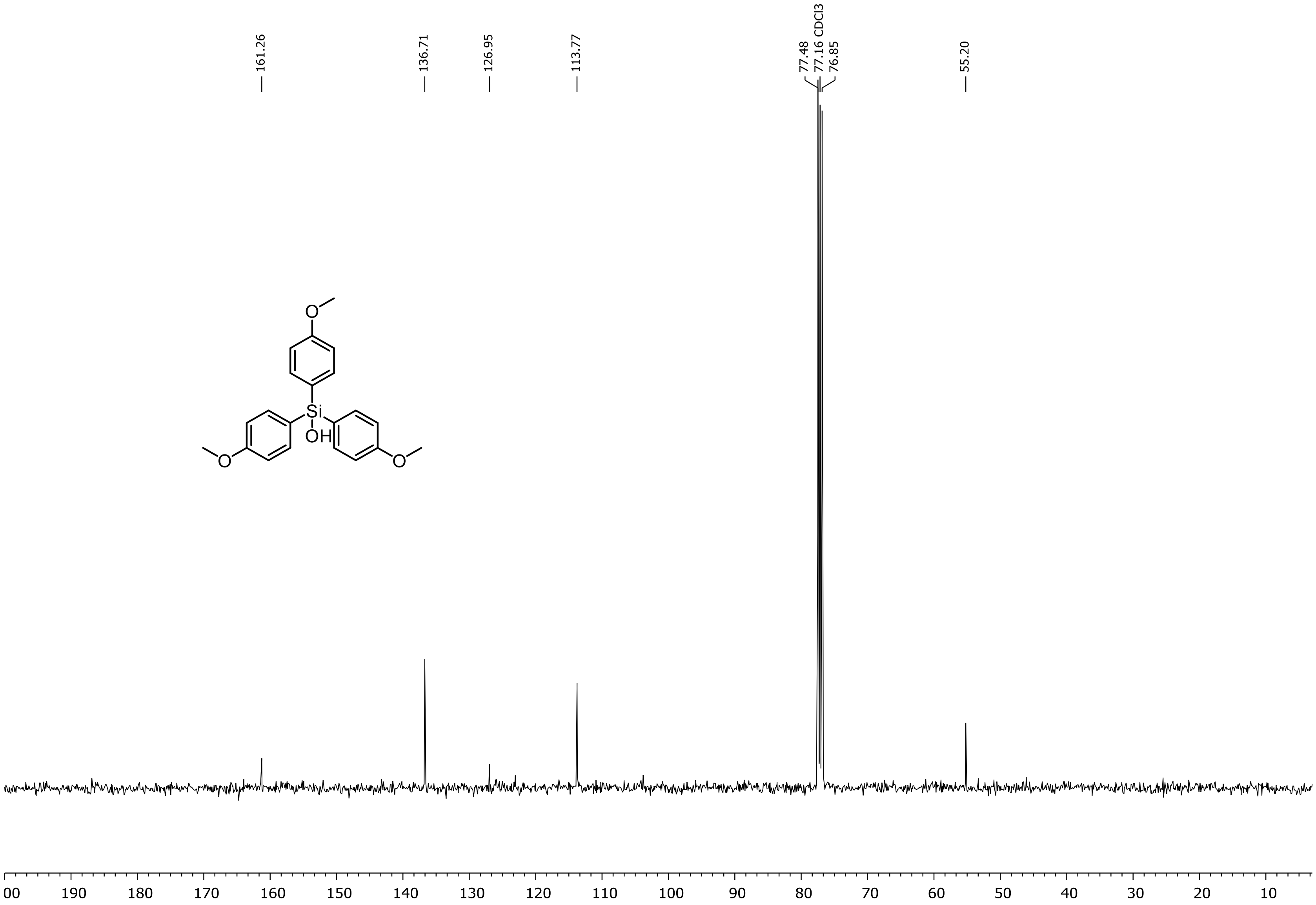

^{1}H – ^{29}Si HMBC NMR spectrum of tris(4-methoxyphenyl)silanol (6b) (400-80 MHz, $CDCl_3$)

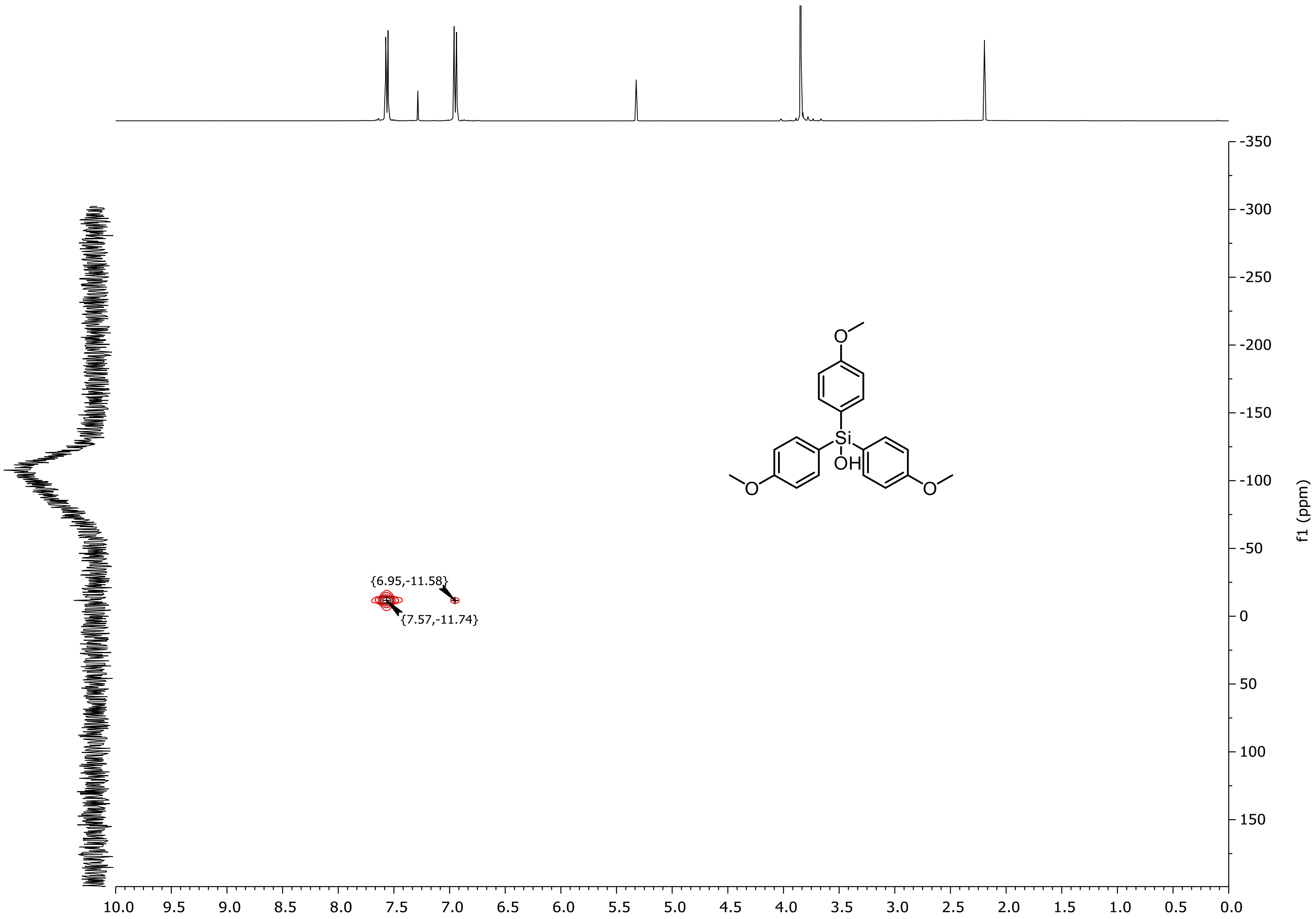

^{1}H NMR spectrum of tris(4-(*t*-butyl)phenyl)silanol (6d) (400 MHz, $CDCl_3$)

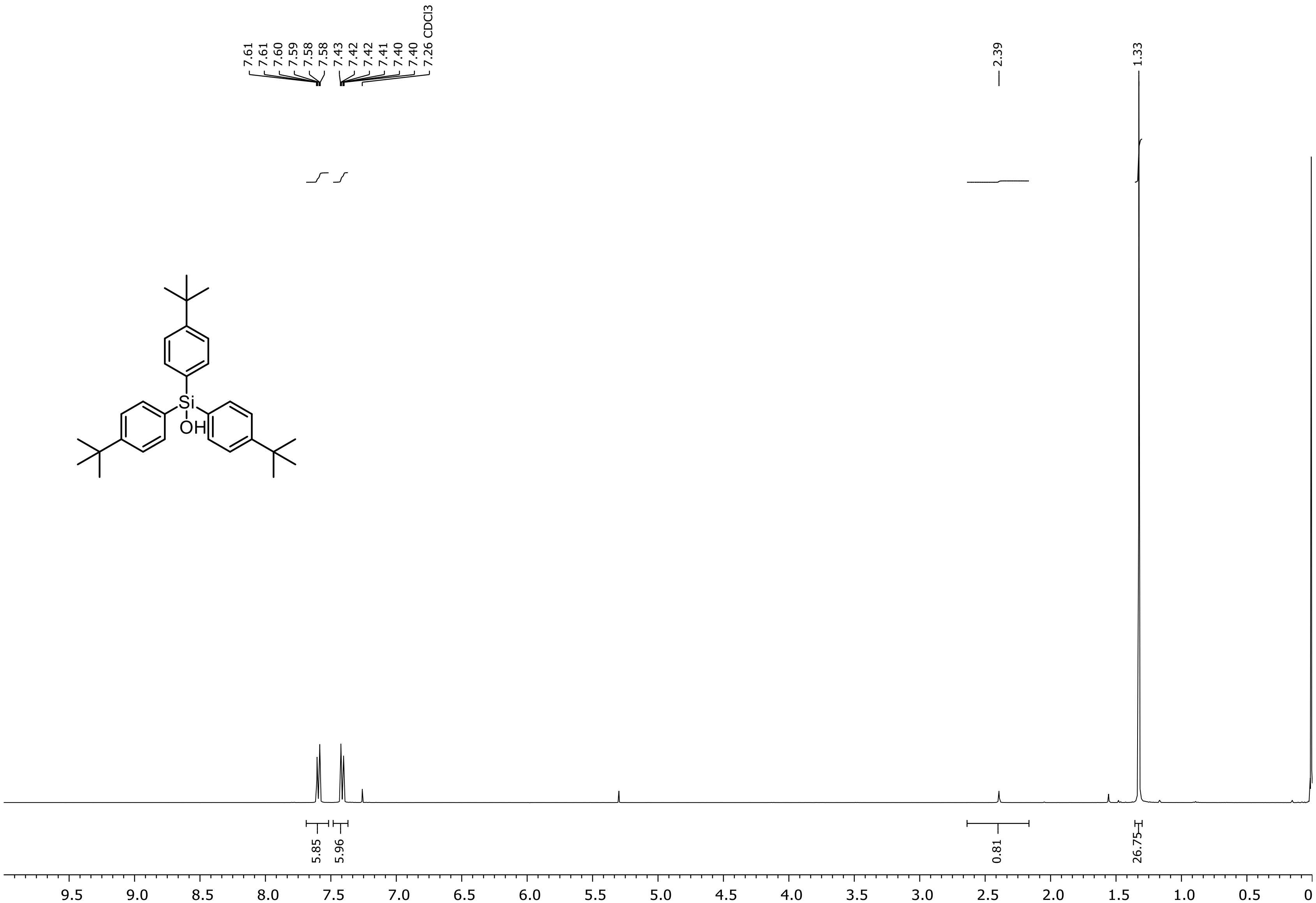

$^{13}C\{^{1}H\}$ NMR spectrum tris(4-(*t*-butyl)phenyl)silanol (6d) (101 MHz, $CDCl_3$)

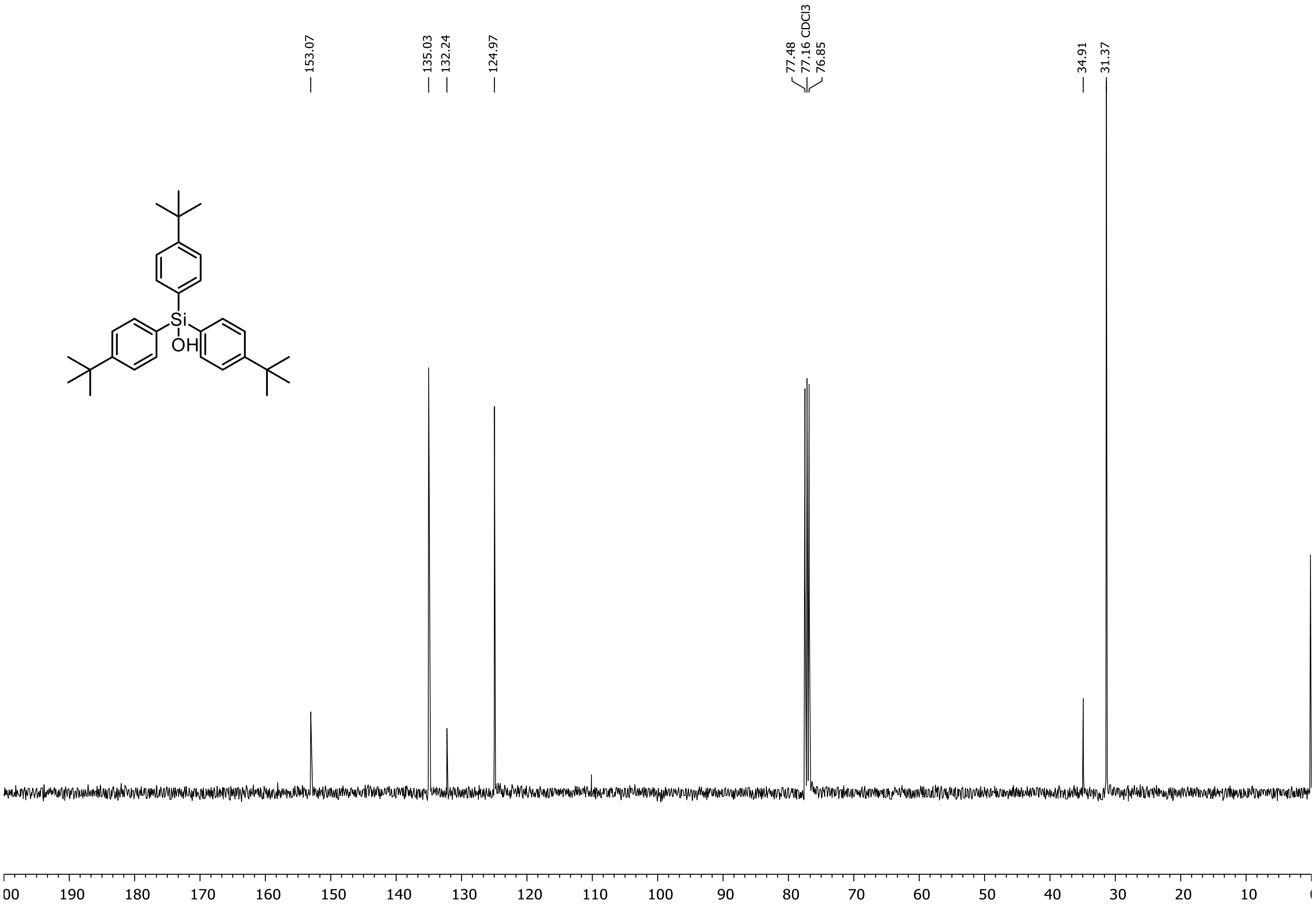

^{1}H – ^{29}Si HMBC NMR spectrum of tris(4-(*t*-butyl)phenyl)silanol (6d) (400-80 MHz, $CDCl_3$)

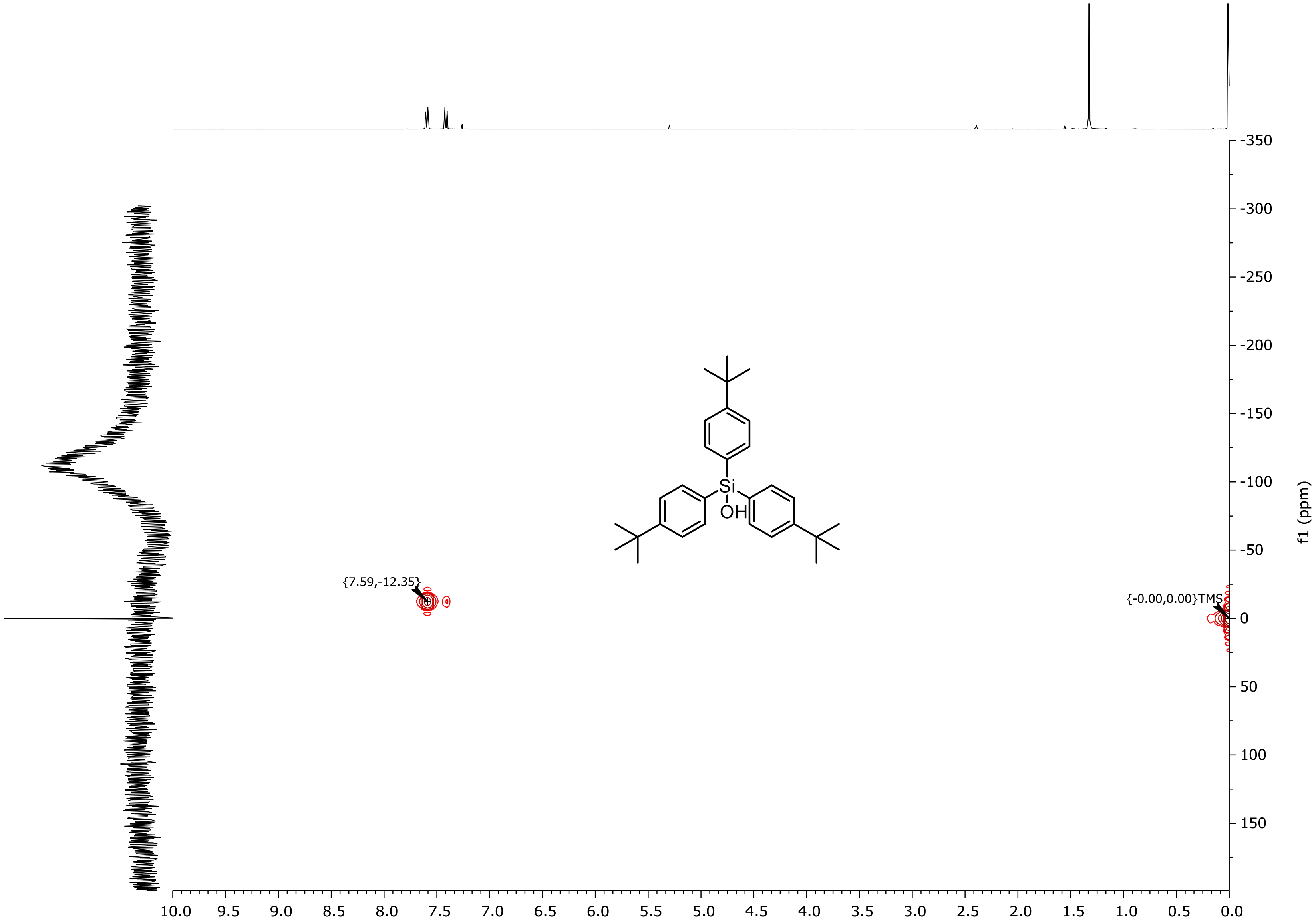

^{1}H NMR spectrum of tris(3-chlorophenyl)silanol (6g) (400 MHz, CDCl$_3$)

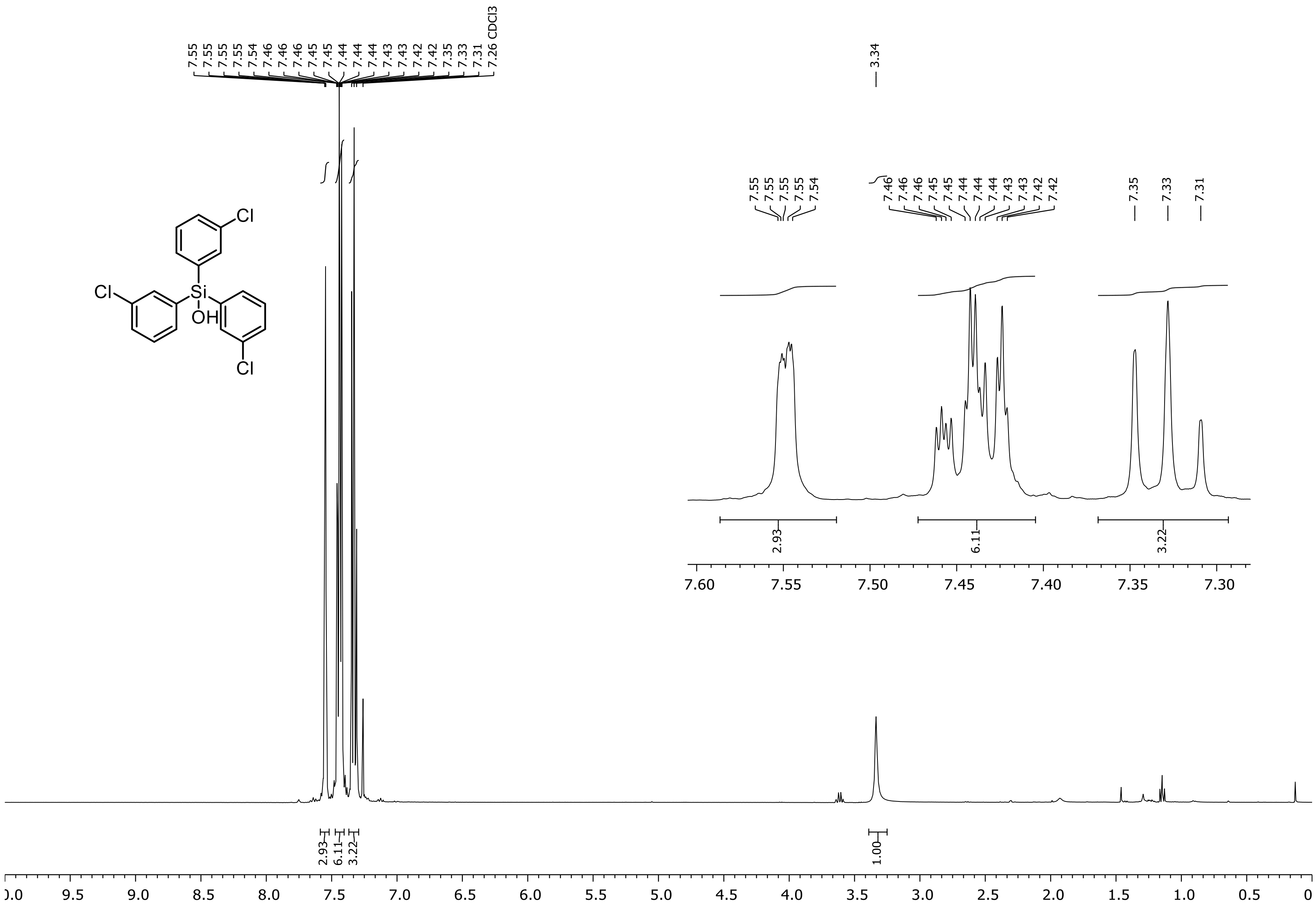

$^{13}C\{^1H\}$ NMR spectrum tris(3-chlorophenyl)silanol (6g) (101 MHz, $CDCl_3$)

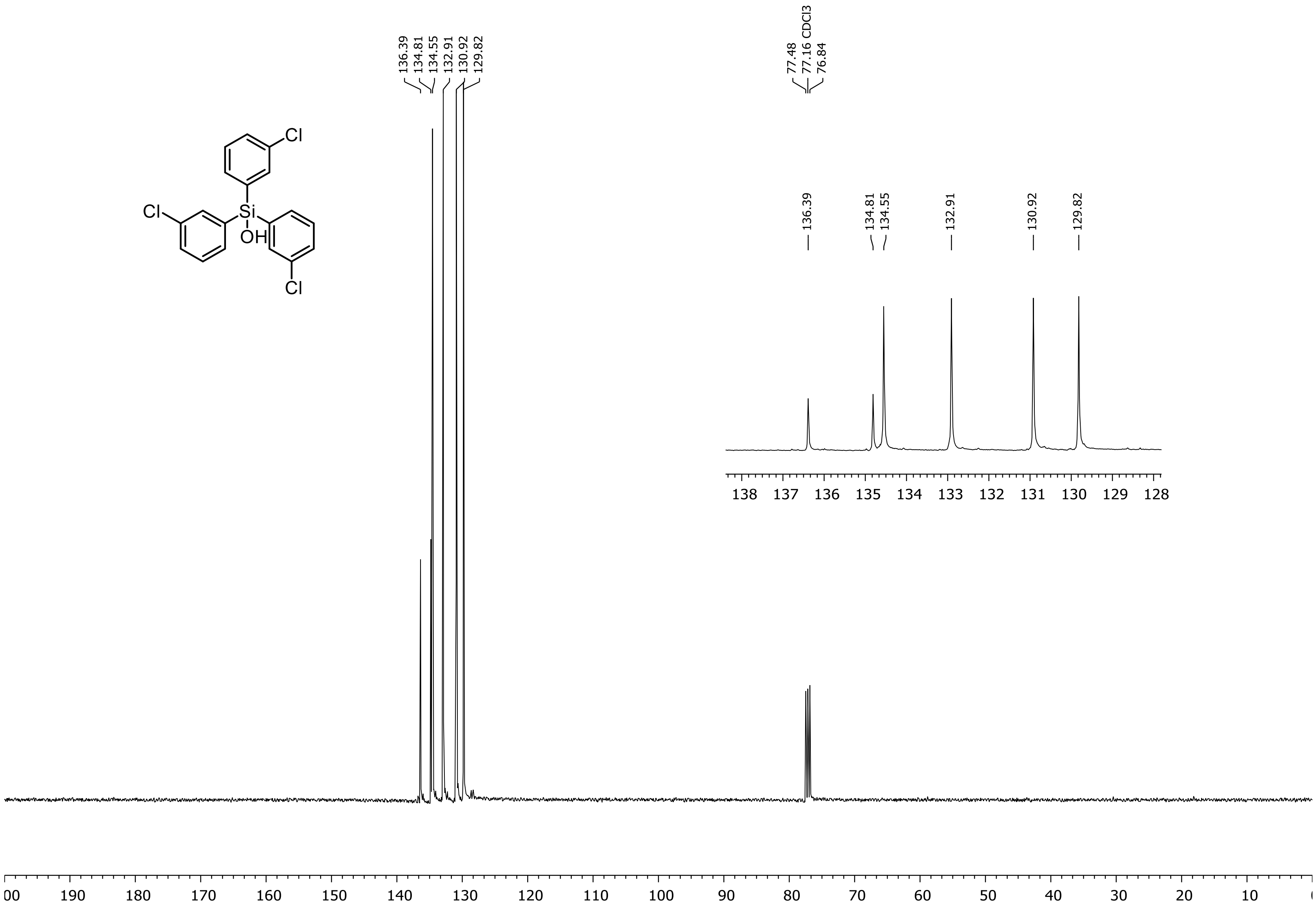

$^{29}Si\{^{1}H\}$ NMR spectrum of tris(3-chlorophenyl)silanol (6g) (80 MHz, $CDCl_3$)

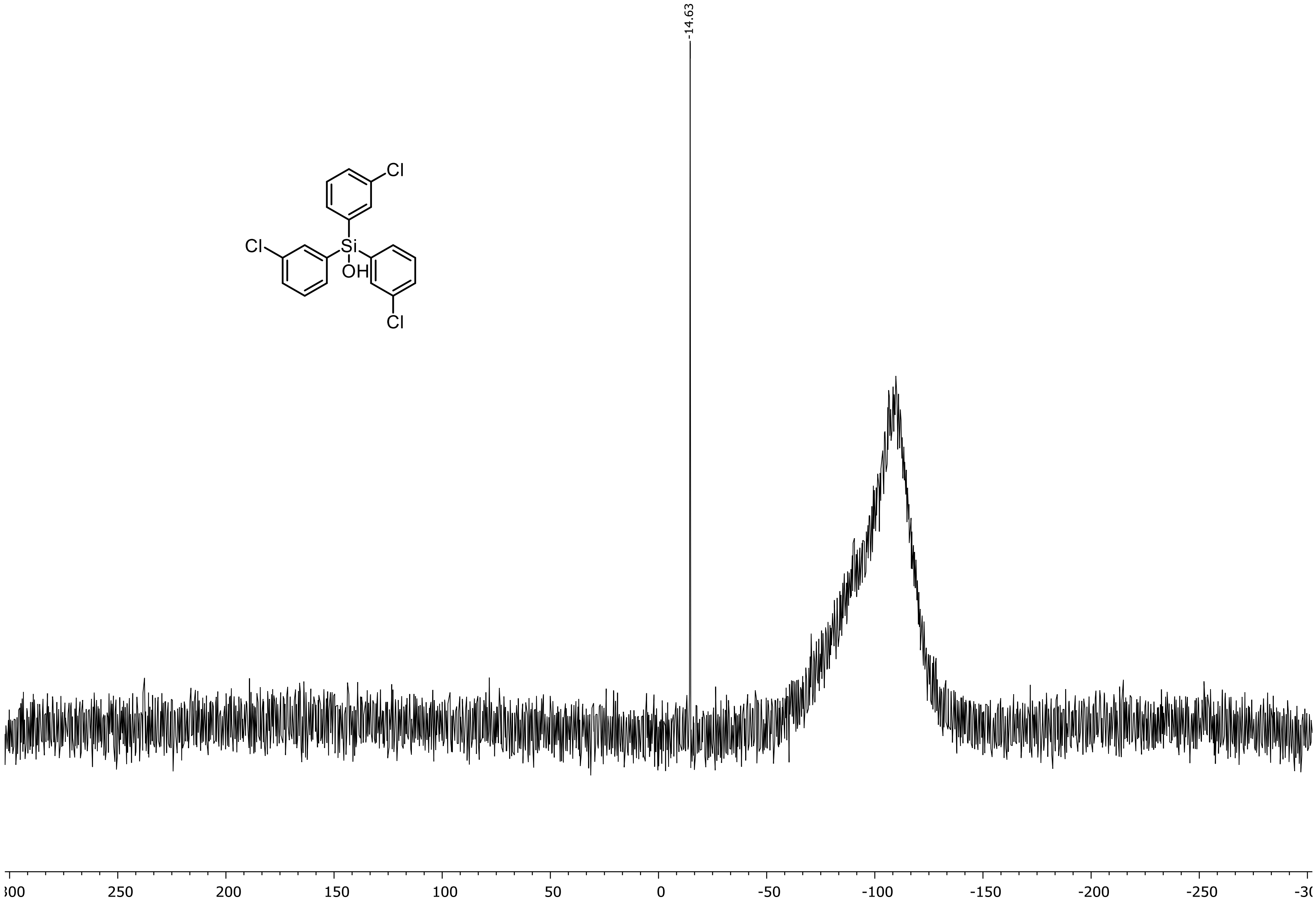

^{1}H NMR spectrum of tris(3-(trifluoromethyl)phenyl)silanol (6i) (400 MHz, $CDCl_3$)

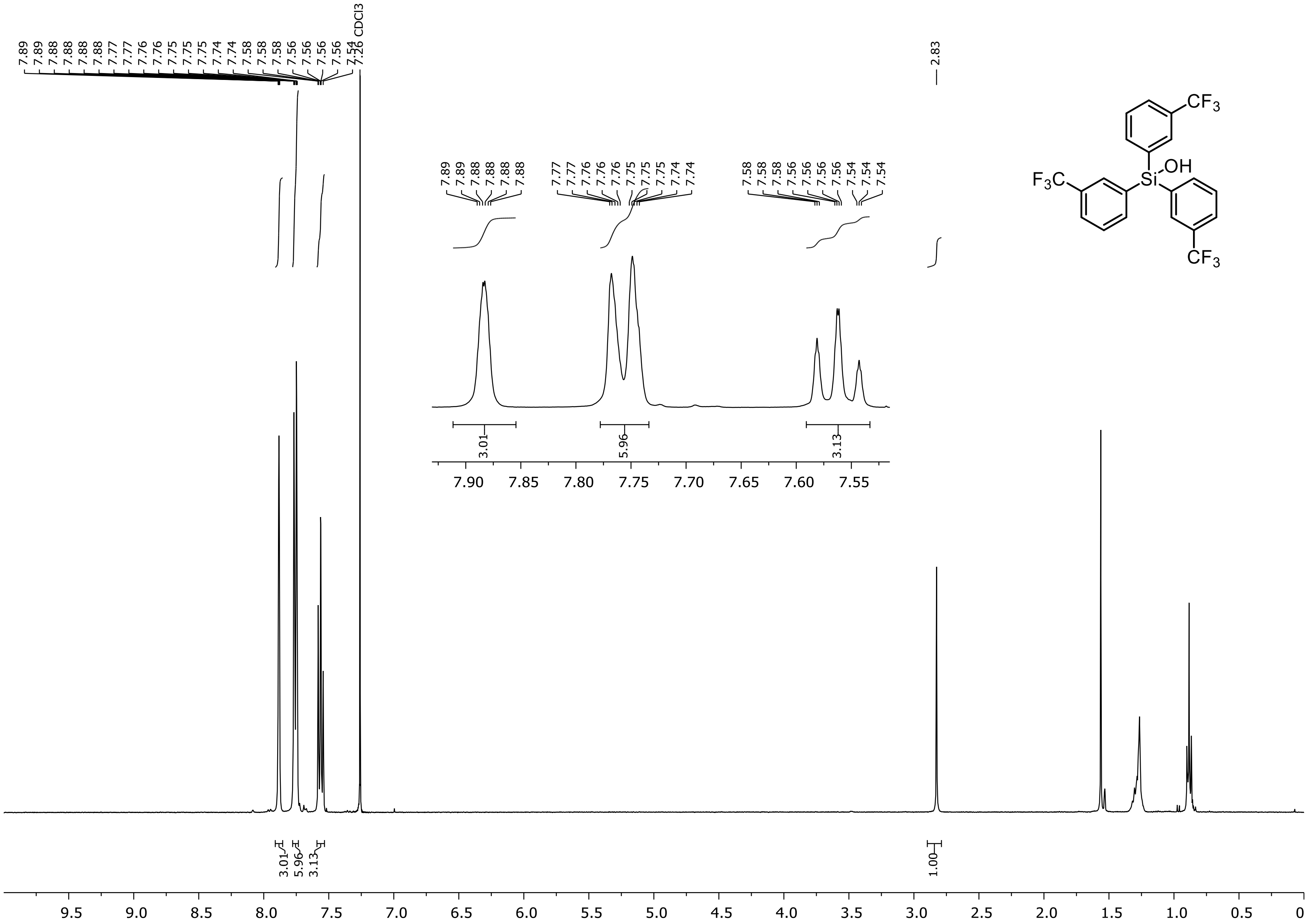

$^{13}C\{^{1}H\}$ NMR spectrum of tris(3-(trifluoromethyl)phenyl)silanol (6i) (101 MHz, $CDCl_3$)

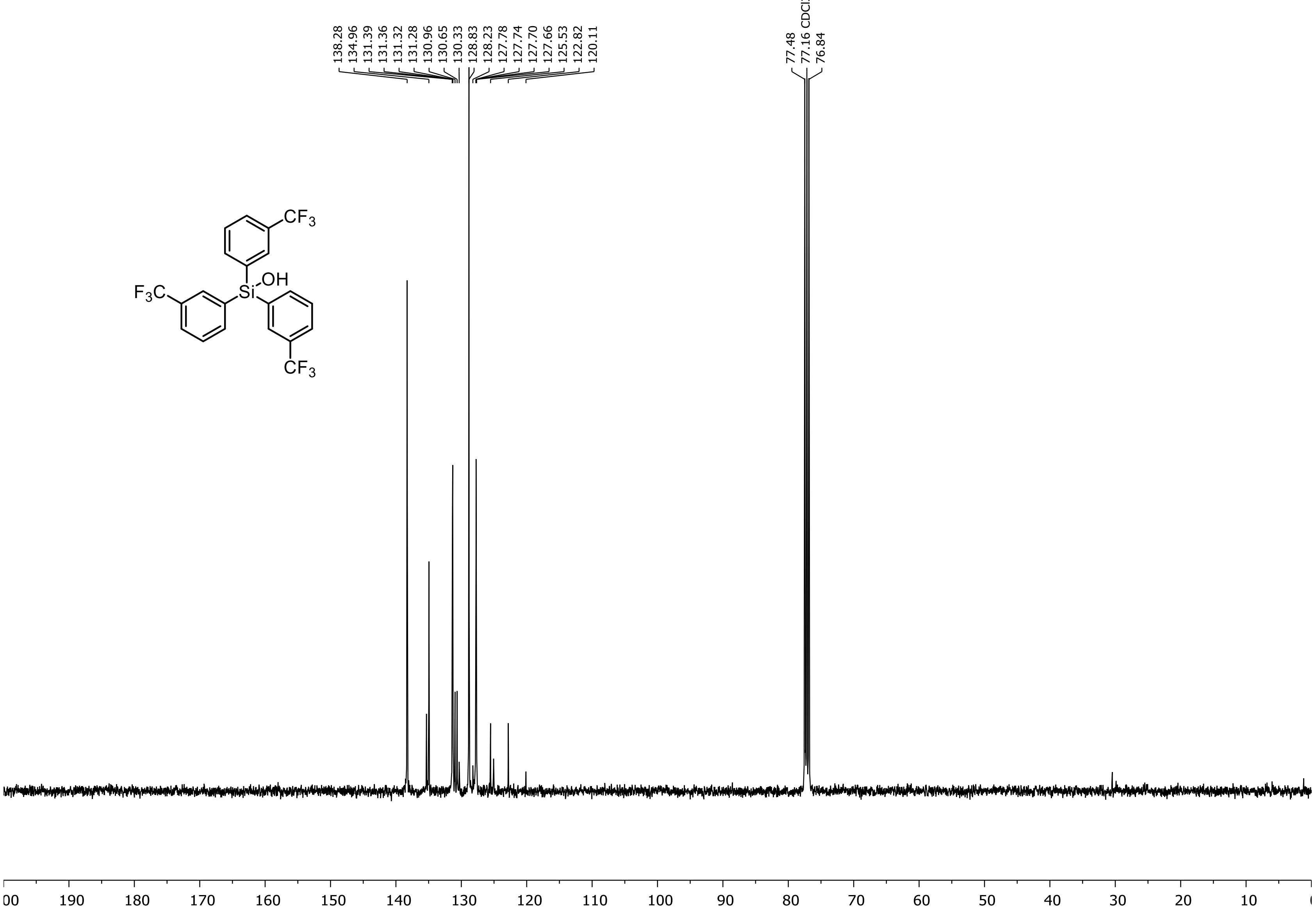

Expanded $^{13}C\{^1H\}$ NMR spectrum of tris(3-(trifluoromethyl)phenyl)silanol (6i) 140 – 1119 ppm (101 MHz, CDCl₃)

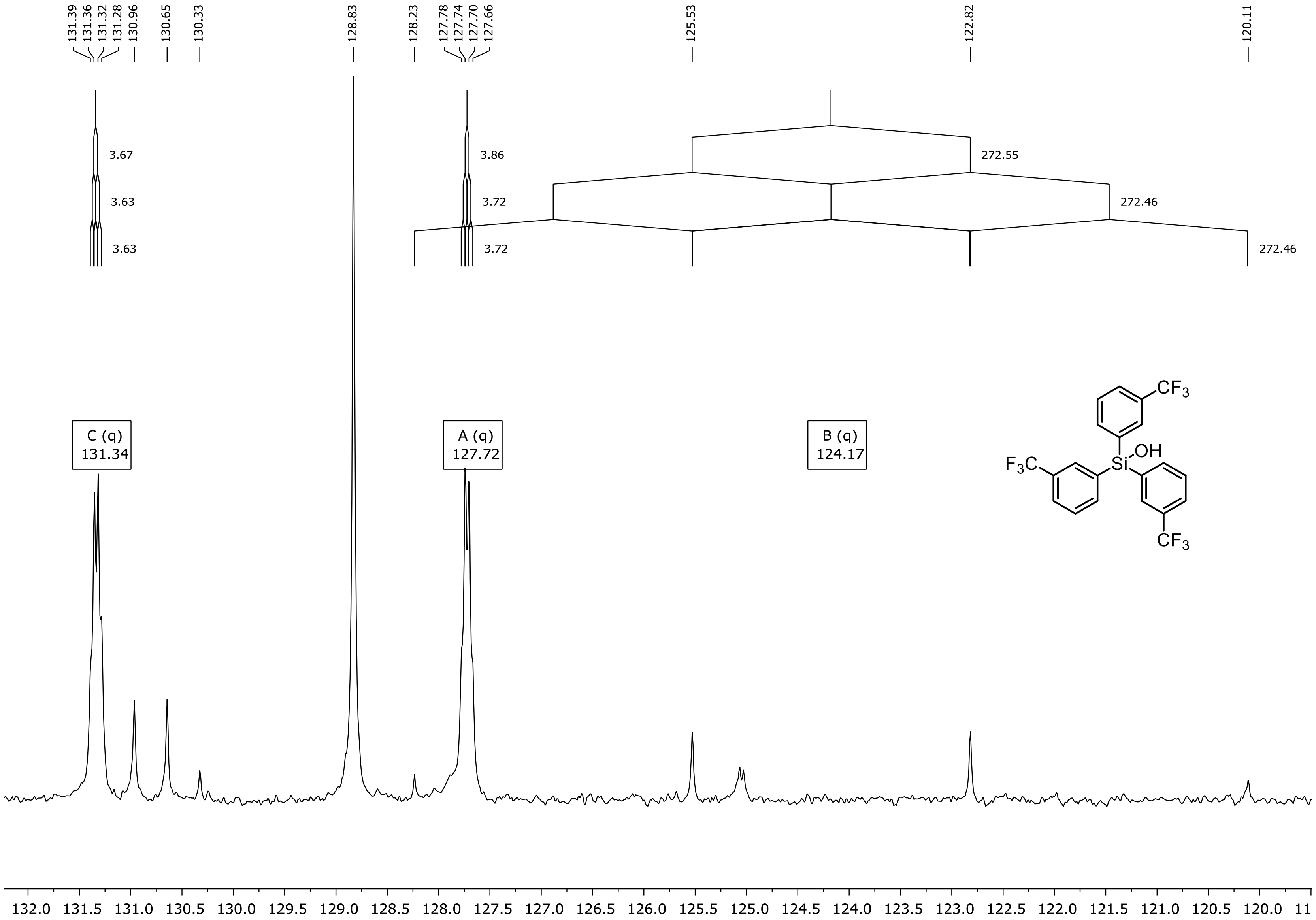

$^{19}F\{^{1}H\}$ NMR spectrum of tris(3-(trifluoromethyl)phenyl)silanol (6i) (377 MHz, $CDCl_3$)

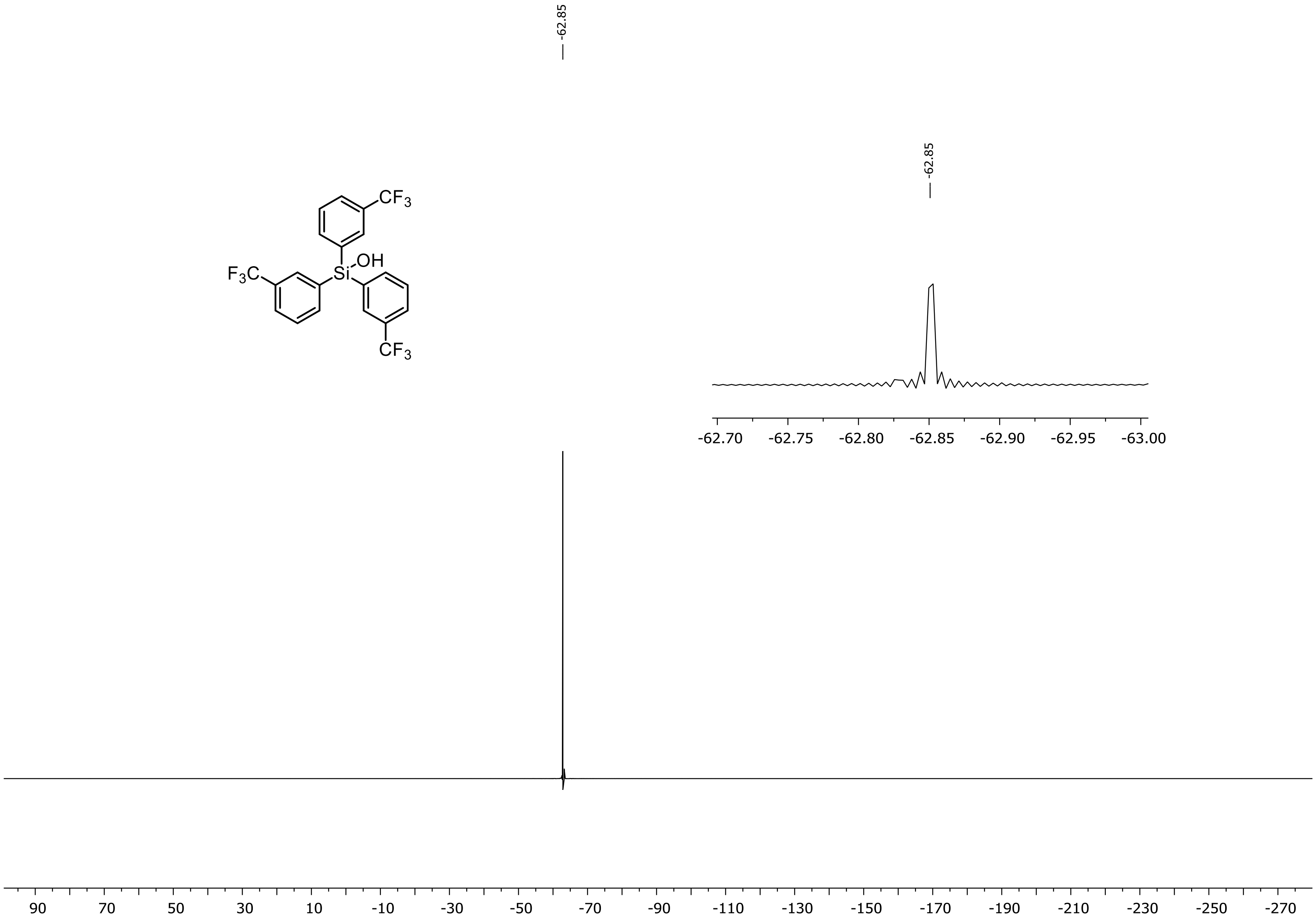

$^{29}Si\{^{1}H\}$ NMR spectrum of tris(3-(trifluoromethyl)phenyl)silanol (6i) (80 MHz, $CDCl_3$)

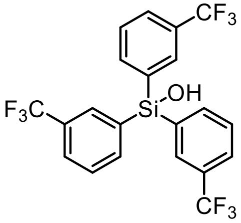

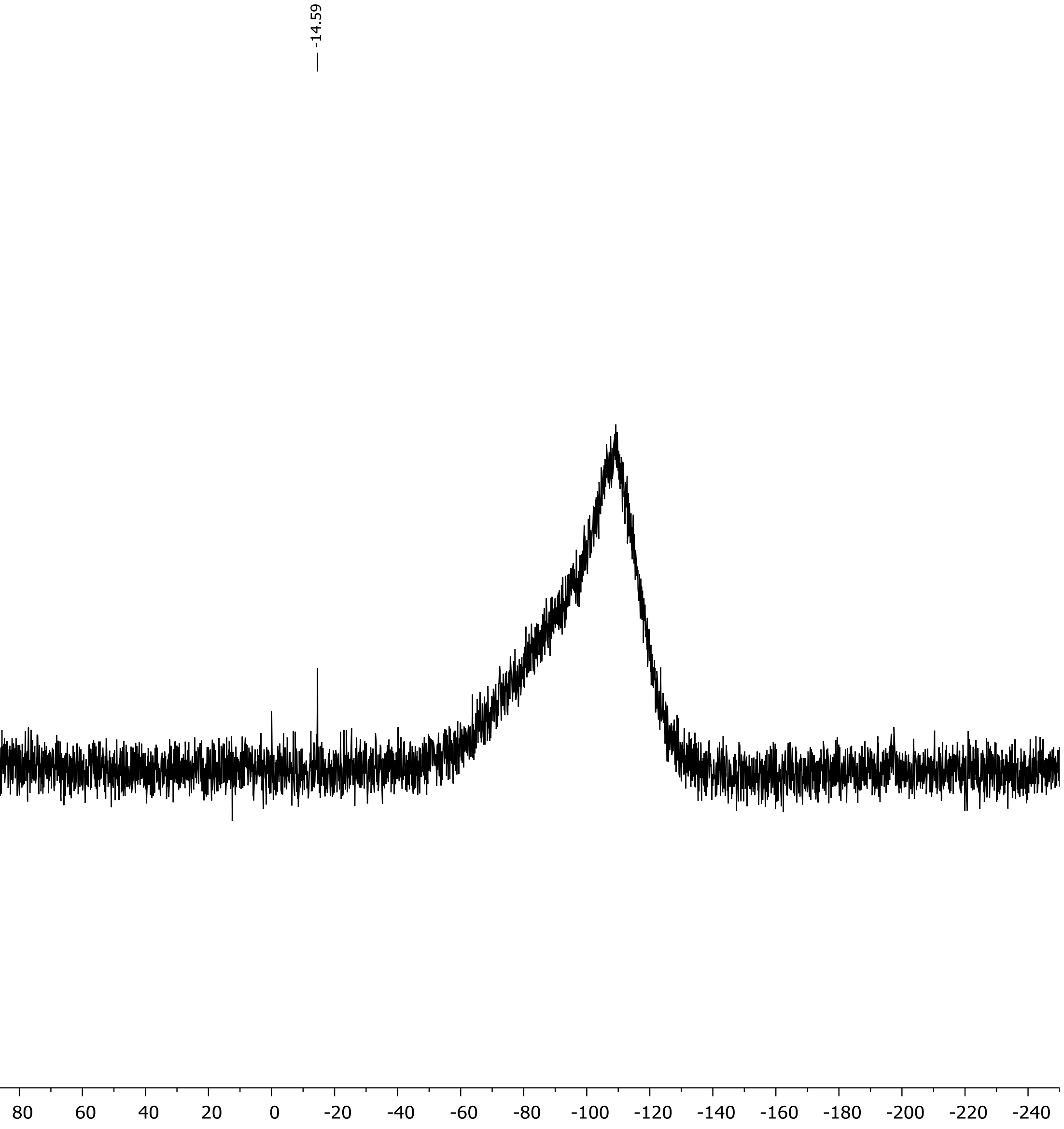

^{1}H NMR spectrum of tris(4-(trifluoromethyl)phenyl)silanol (6j) (400 MHz, $CDCl_3$)

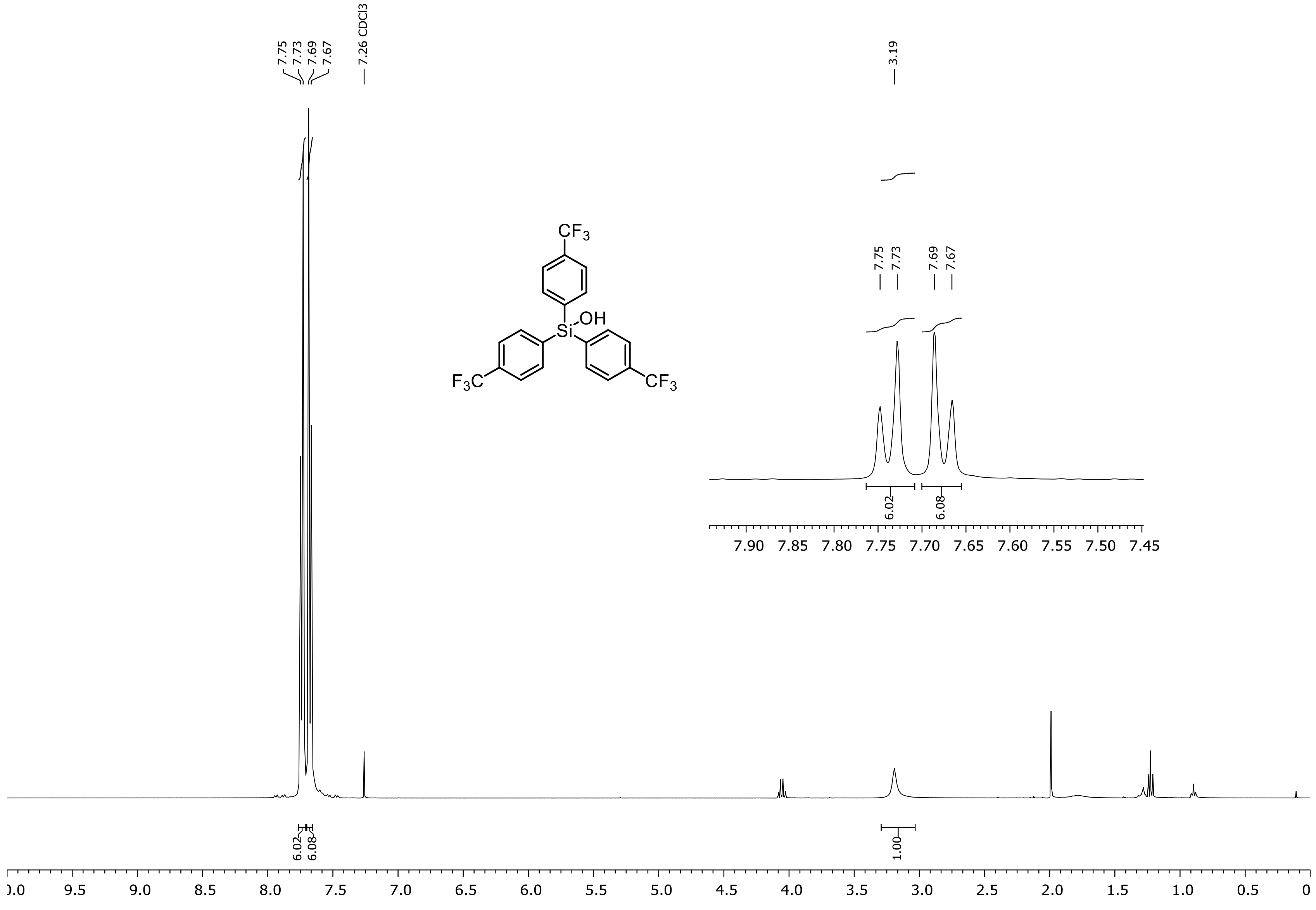

$^{13}C\{^{1}H\}$ NMR spectrum tris(4-(trifluoromethyl)phenyl)silanol (6j) (101 MHz, $CDCl_3$)

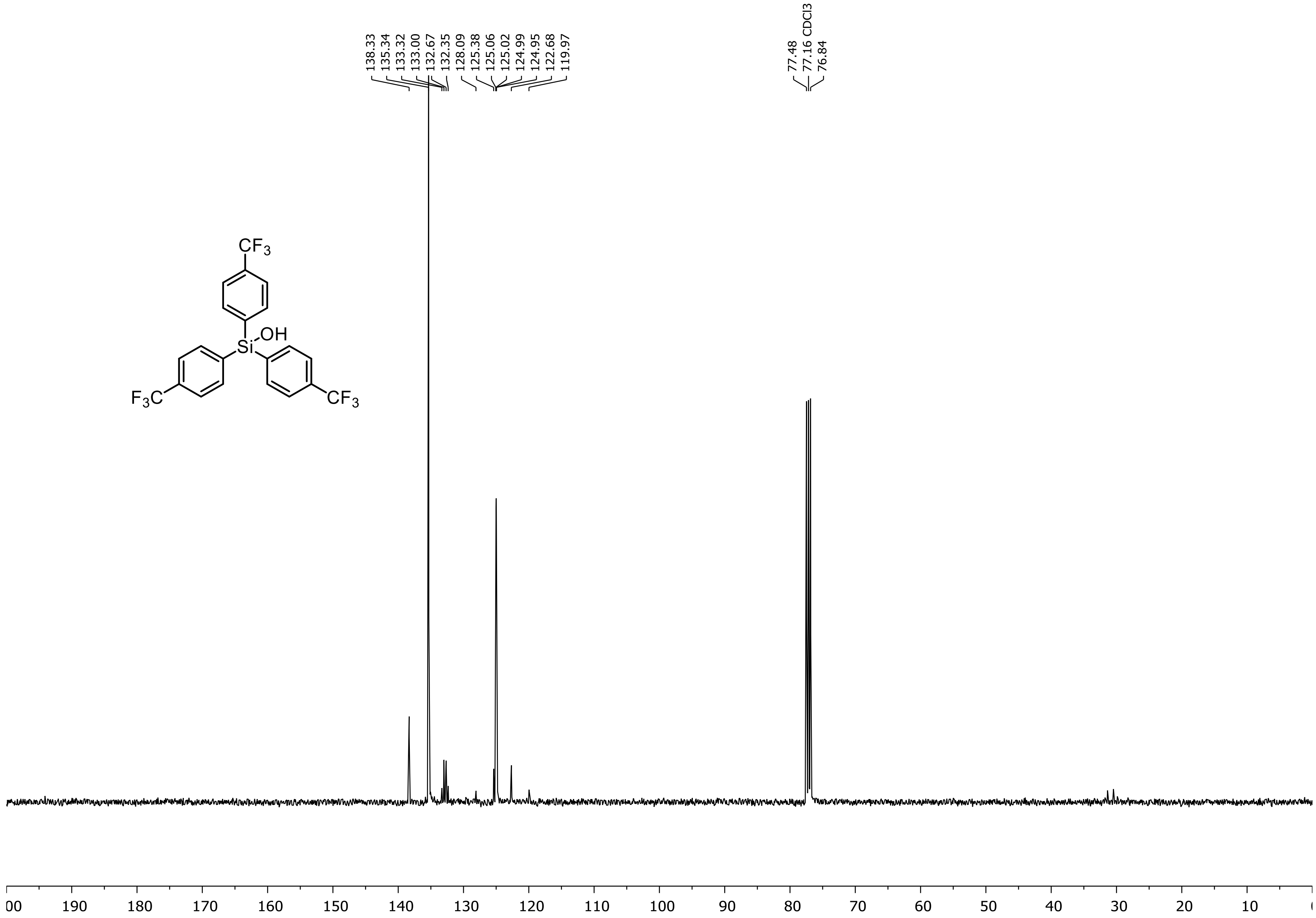

Expanded $^{13}C\{^{1}H\}$ NMR spectrum of tris(4-(trifluoromethyl)phenyl)silanol (6j) 135 – 118 ppm (101 MHz, $CDCl_3$)

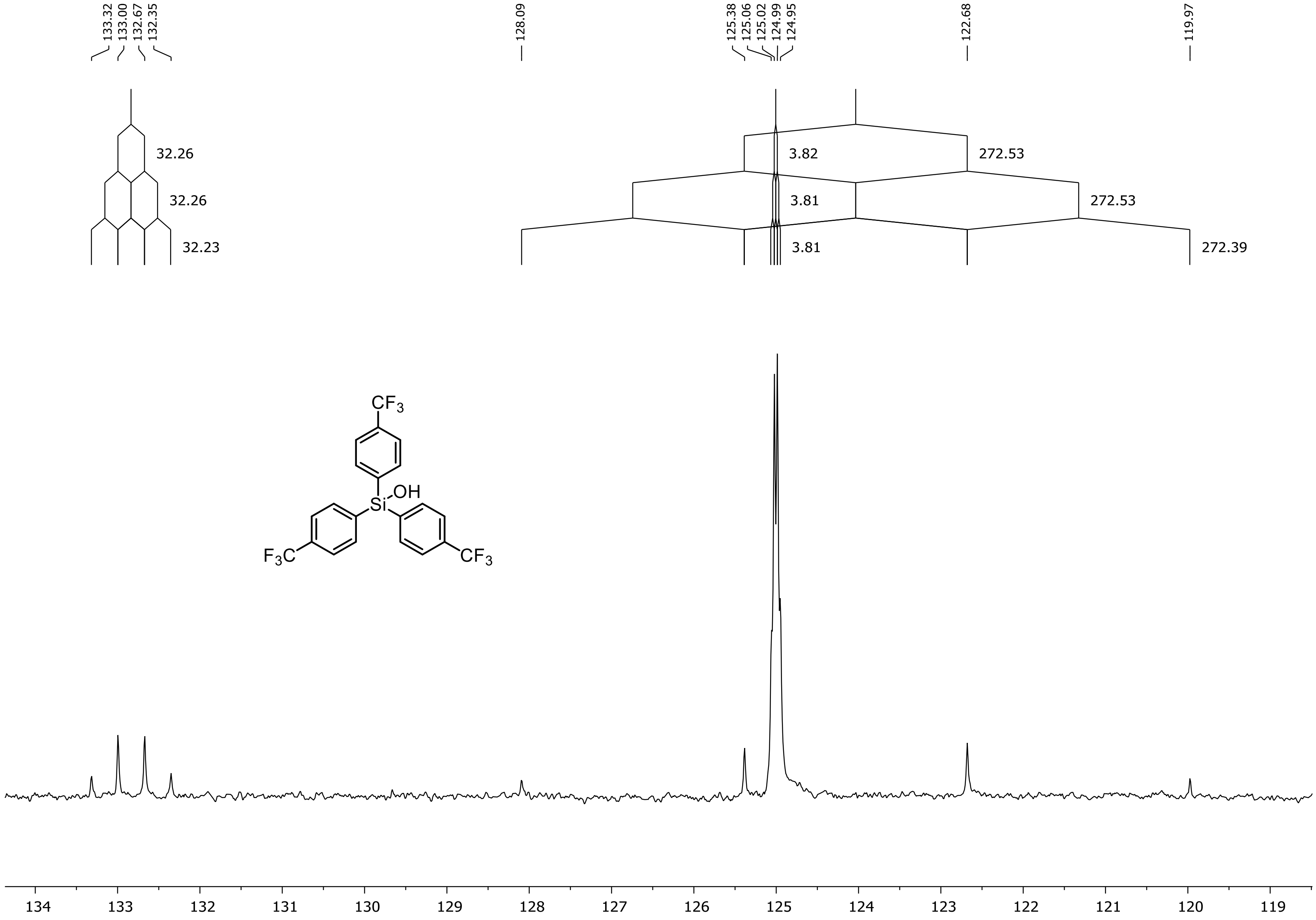

$^{19}F\{^{1}H\}$ NMR spectrum of tris(4-(trifluoromethyl)phenyl)silanol (6j) (377 MHz, $CDCl_3$)

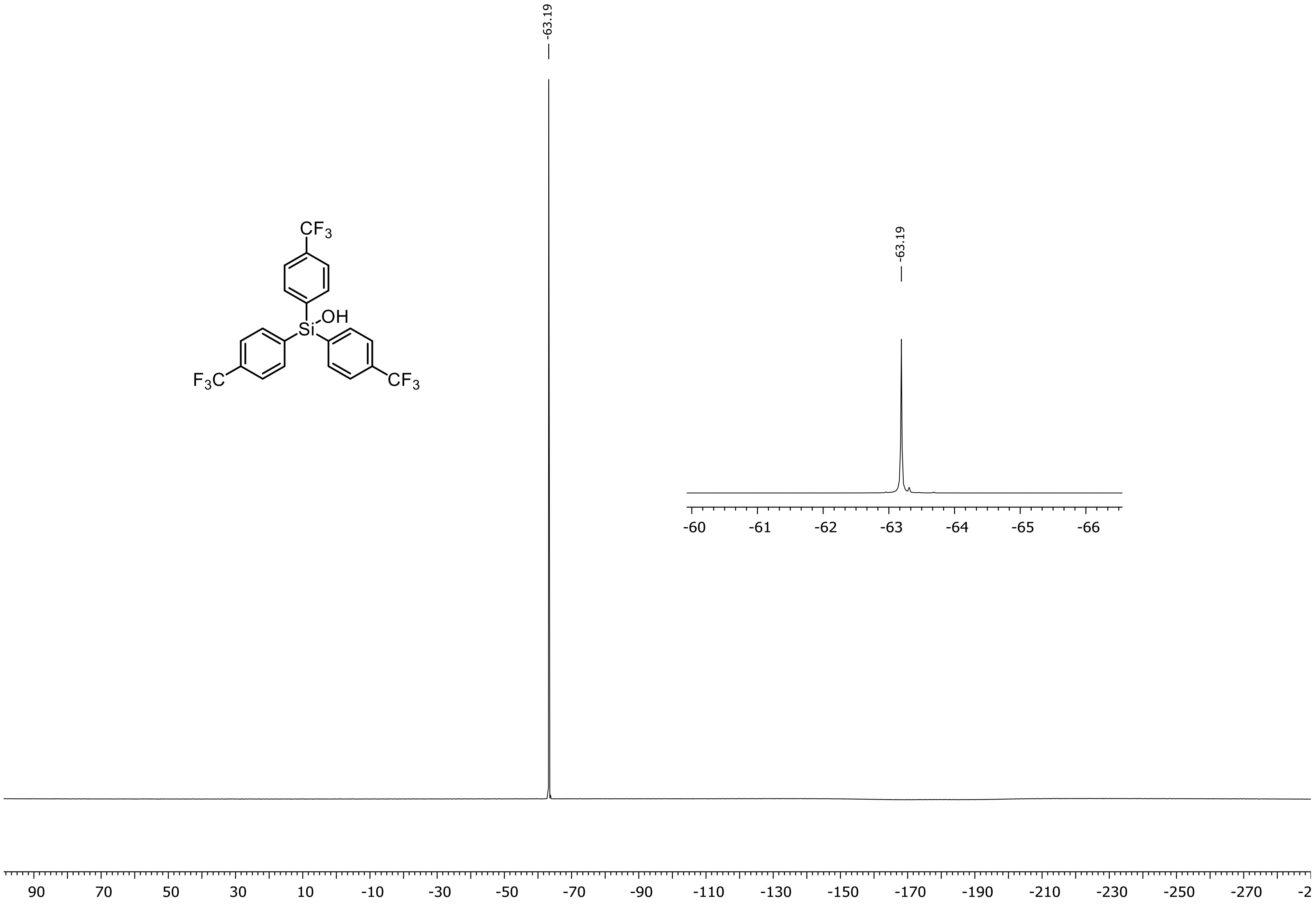

$^{29}Si\{^1H\}$ NMR spectrum of tris(4-(trifluoromethyl)phenyl)silanol (6j) (80 MHz, $CDCl_3$)

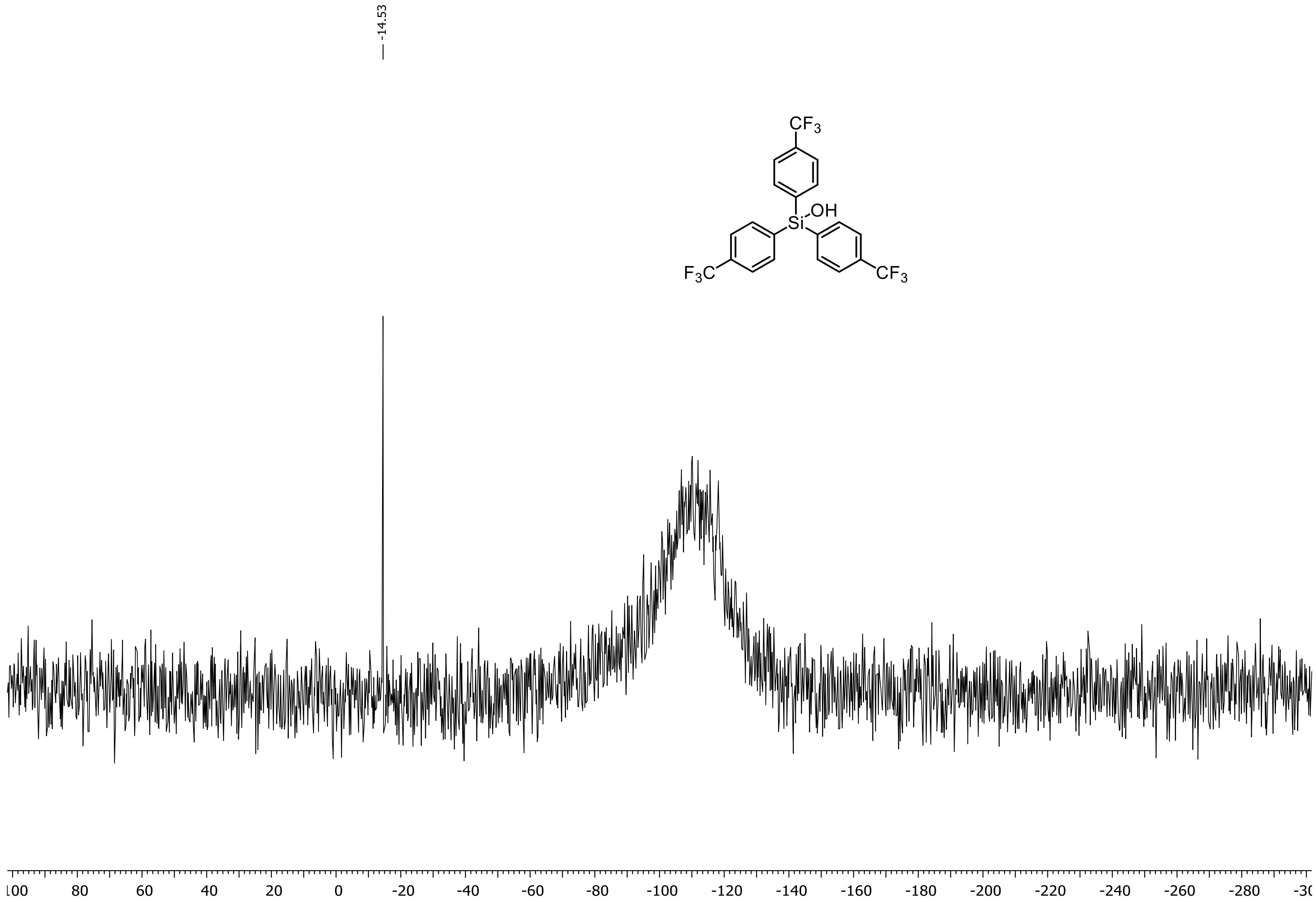

^{1}H NMR spectrum of tris(3,4,5-trifluorophenyl)silanol (6k) (400 MHz, $CDCl_3$)

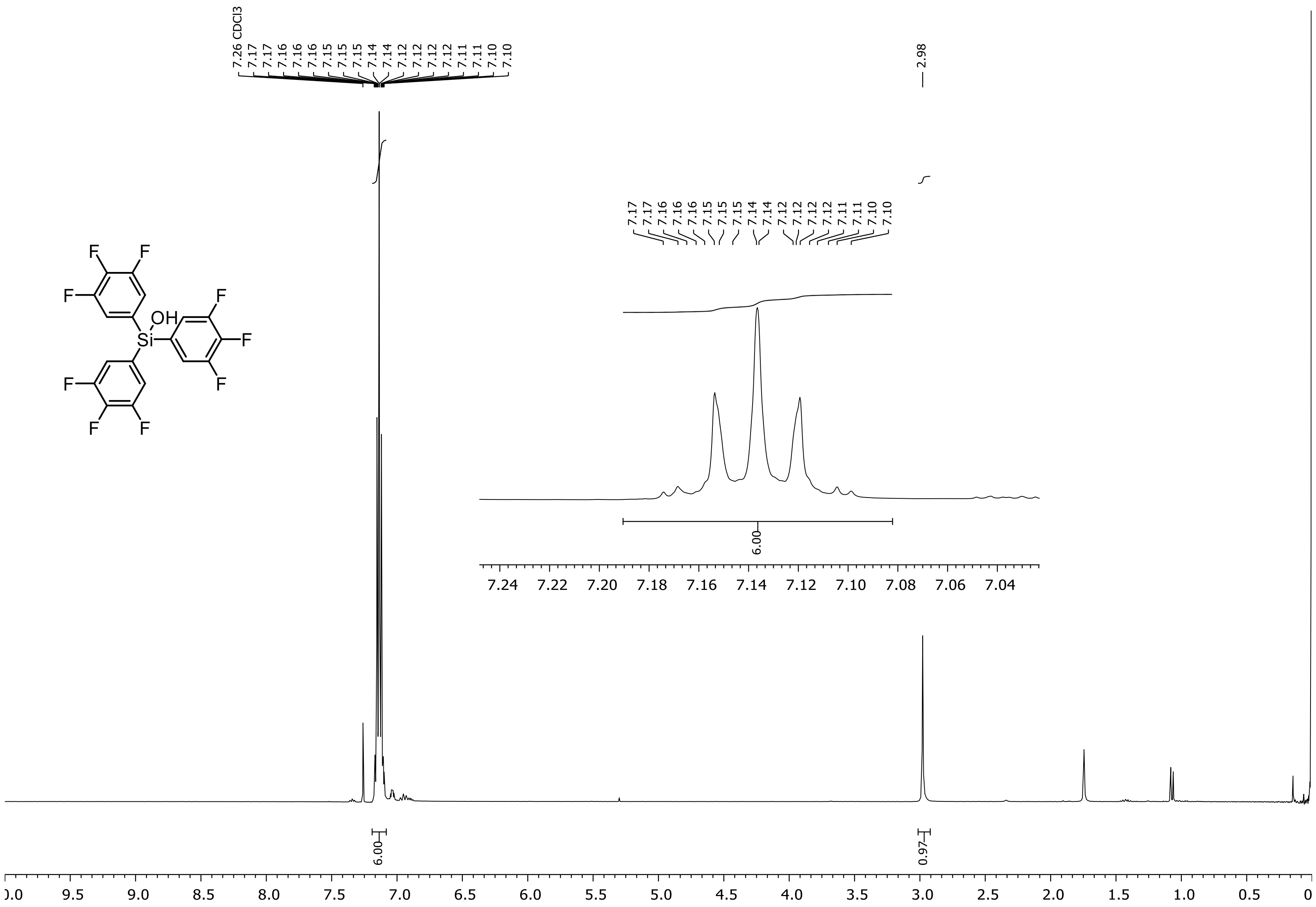

$^{13}C\{^1H\}$ NMR spectrum of tris(3,4,5-trifluorophenyl)silanol (6k) (101 MHz, $CDCl_3$)

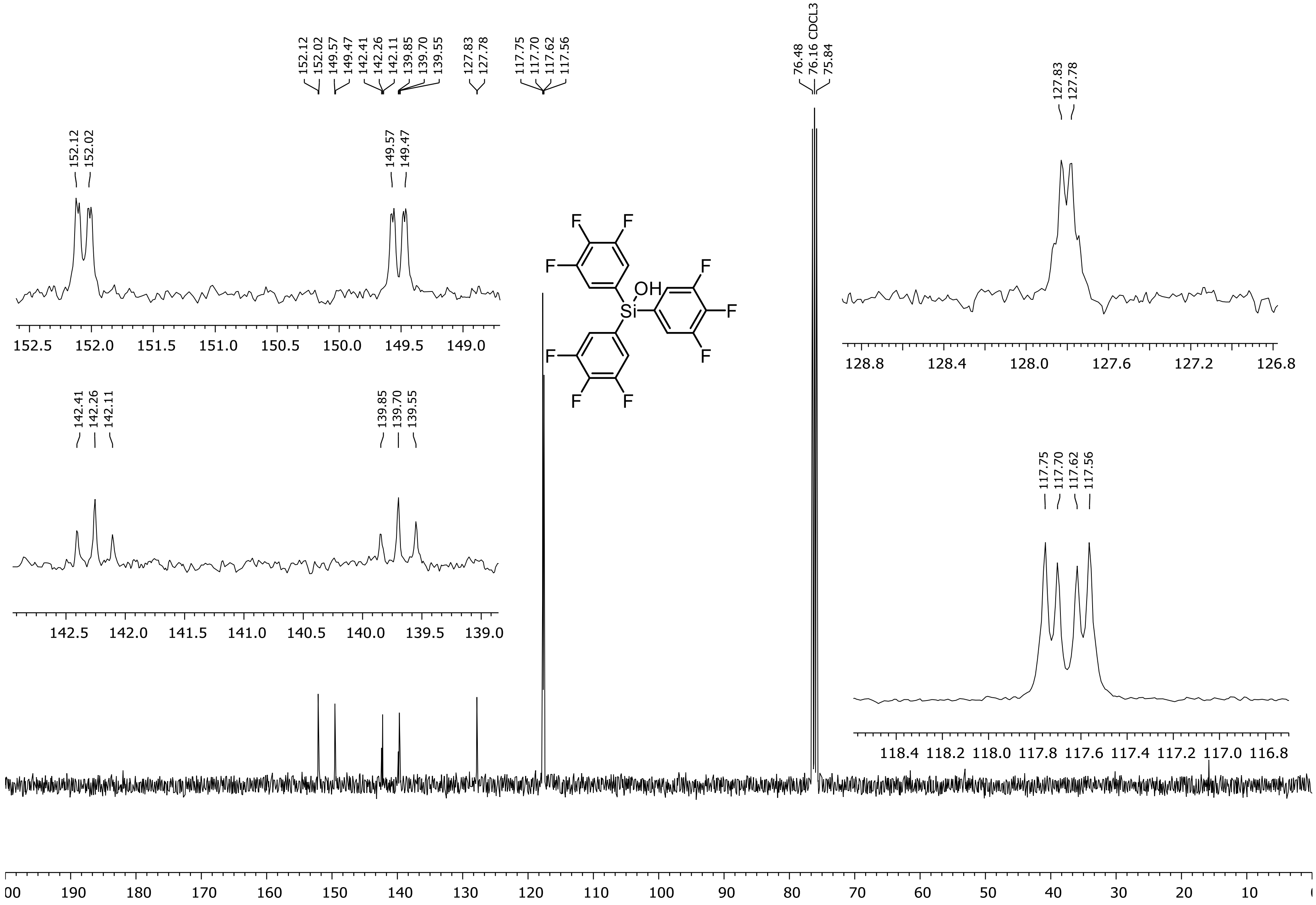

^{19}F NMR spectrum of tris(3,4,5-trifluorophenyl)silanol (6k) (377 MHz, $CDCl_3$)

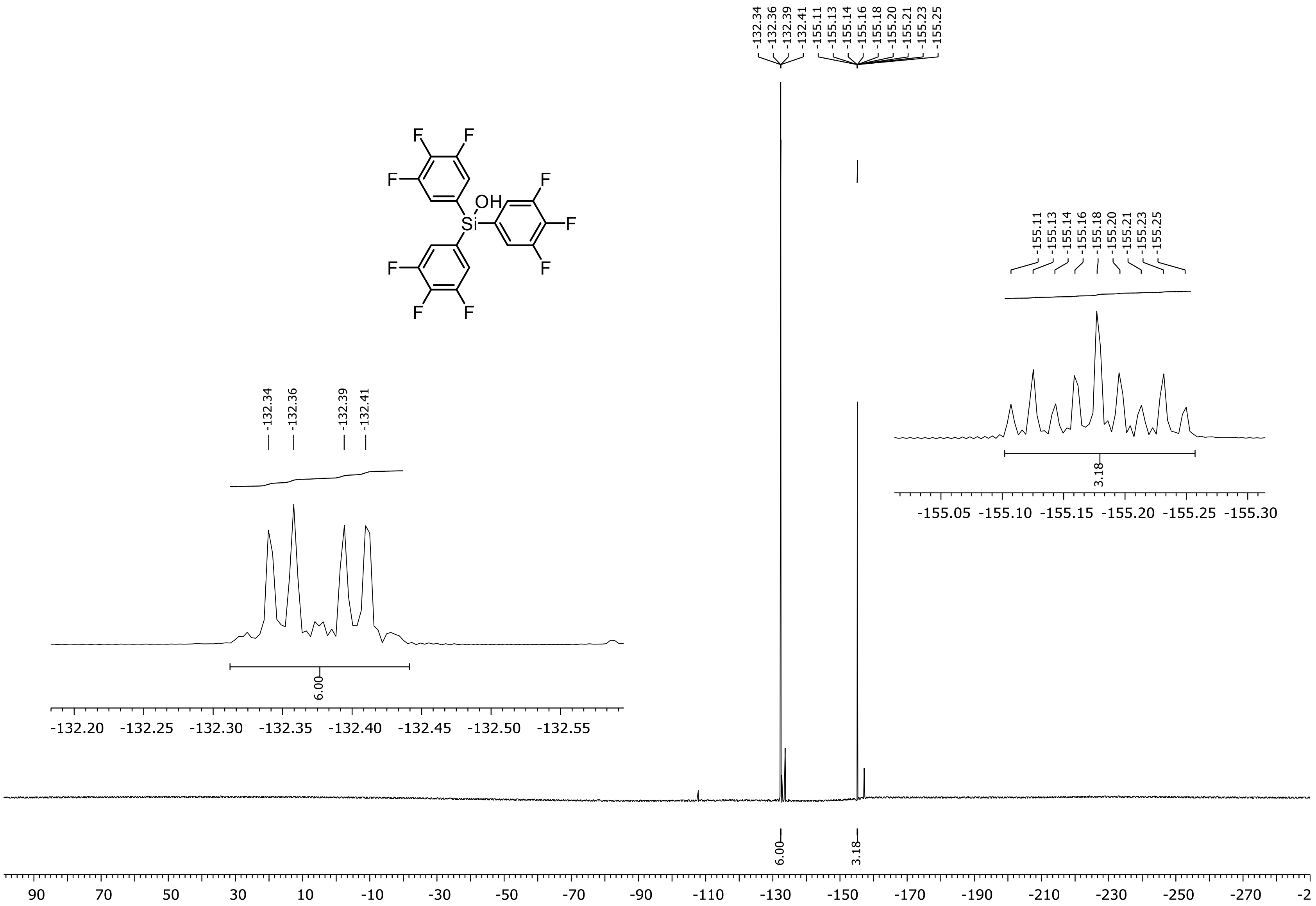

$^{29}Si\{^1H\}$ NMR spectrum of tris(3,4,5-trifluorophenyl)silanol (6k) (80 MHz, $CDCl_3$)

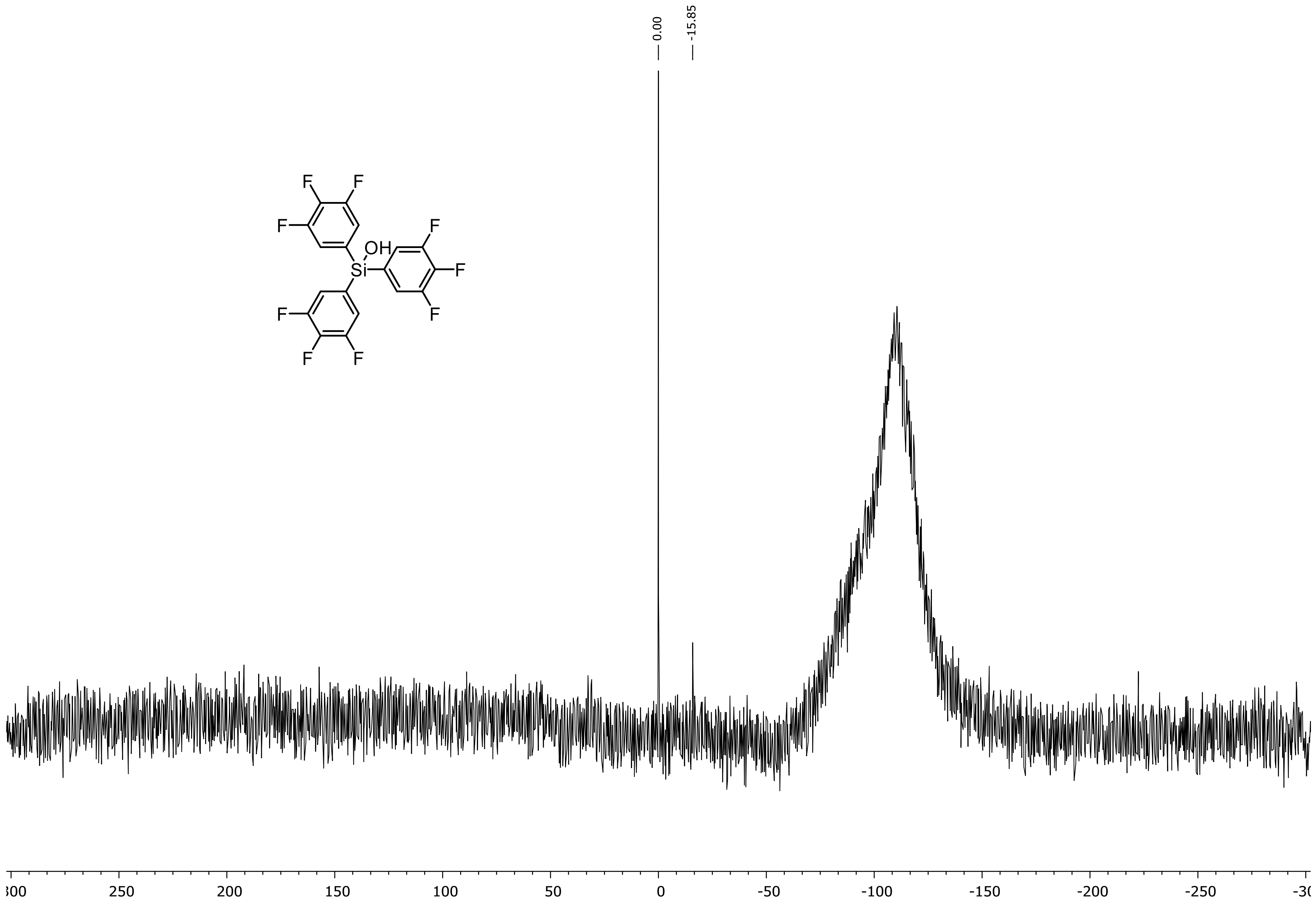

^{1}H NMR spectrum of triphenylsilyl 2-phenylacetate (7) (400 MHz, $CDCl_3$)

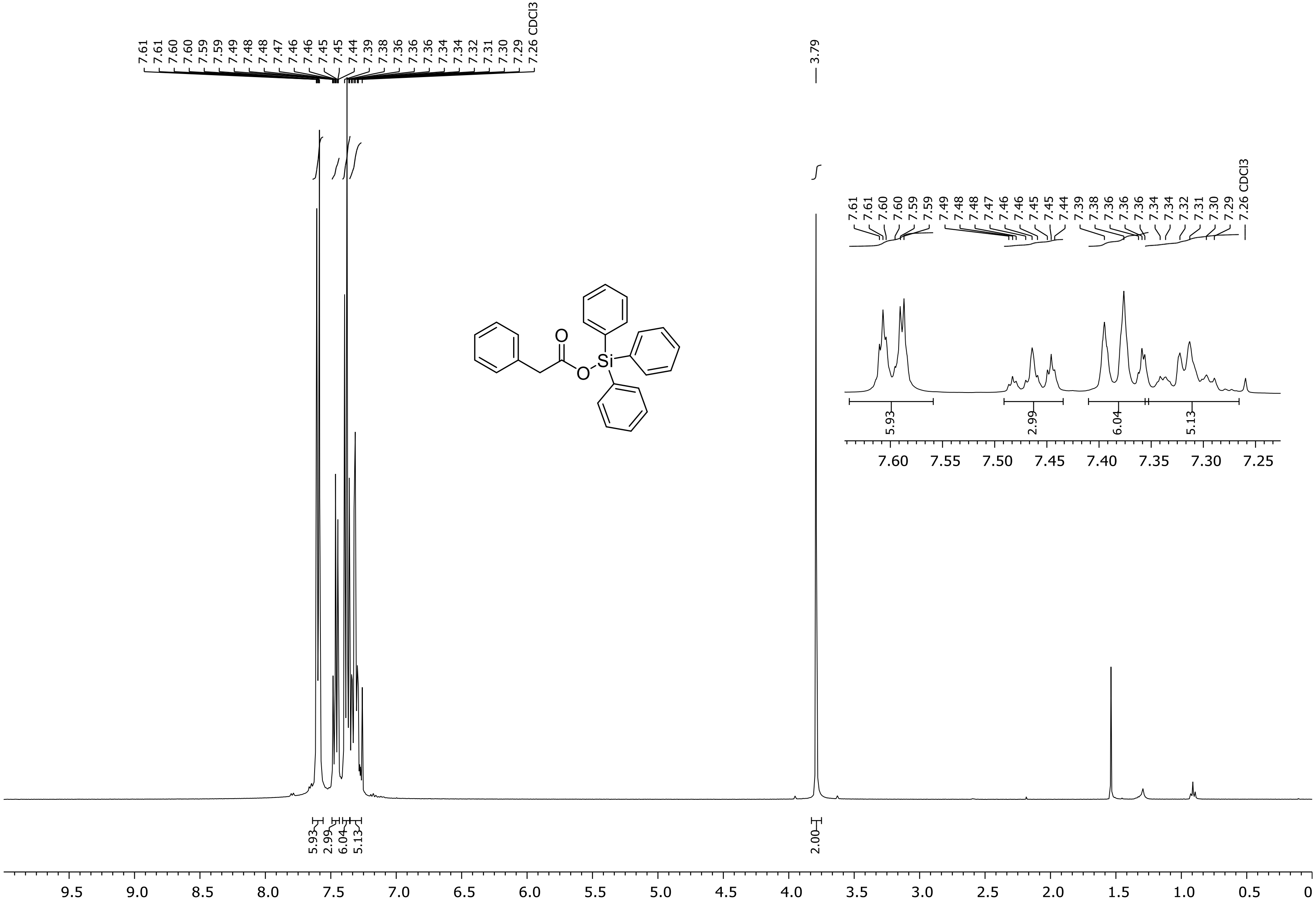

$^{13}C\{^{1}H\}$ NMR spectrum of triphenylsilyl 2-phenylacetate (7) (101 MHz, $CDCl_3$)

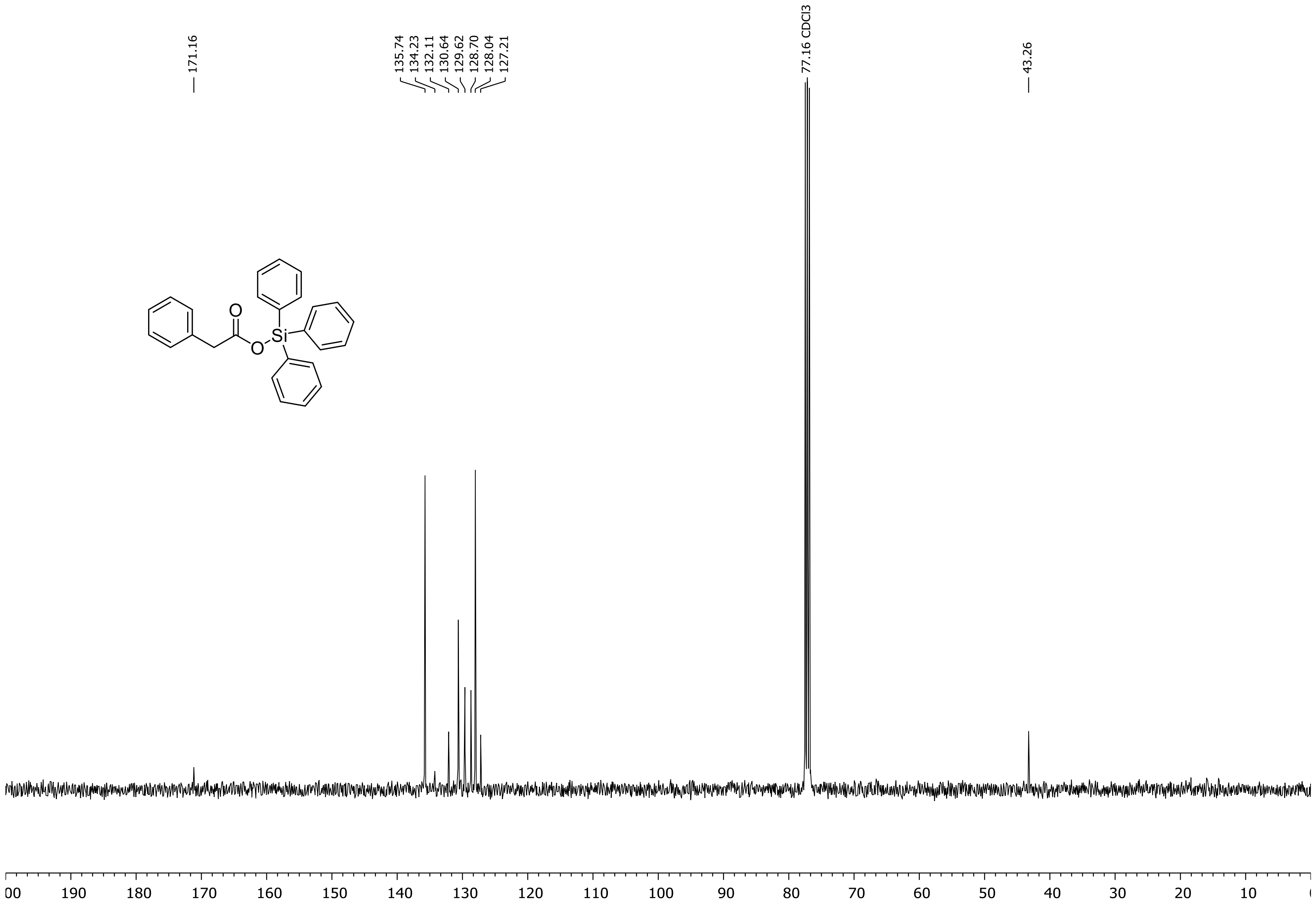

$^{29}Si\{^{1}H\}$ NMR spectrum of triphenylsilyl 2-phenylacetate (7) (80 MHz, $CDCl_3$)

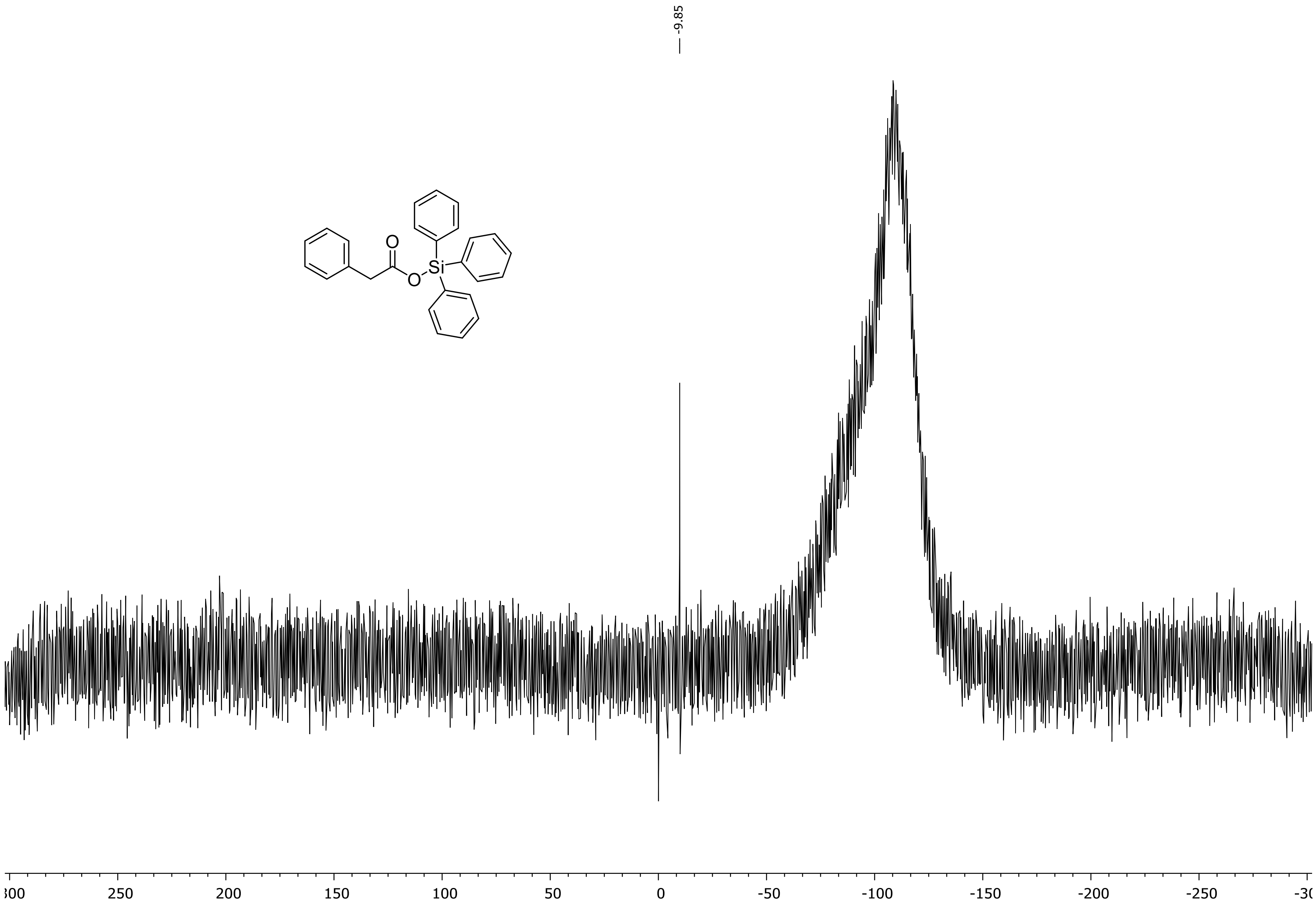

^{1}H NMR spectrum of *N*-(4-methylbenzyl)-1,1,1-triphenylsilanamine (9) (400 MHz, $CDCl_3$)

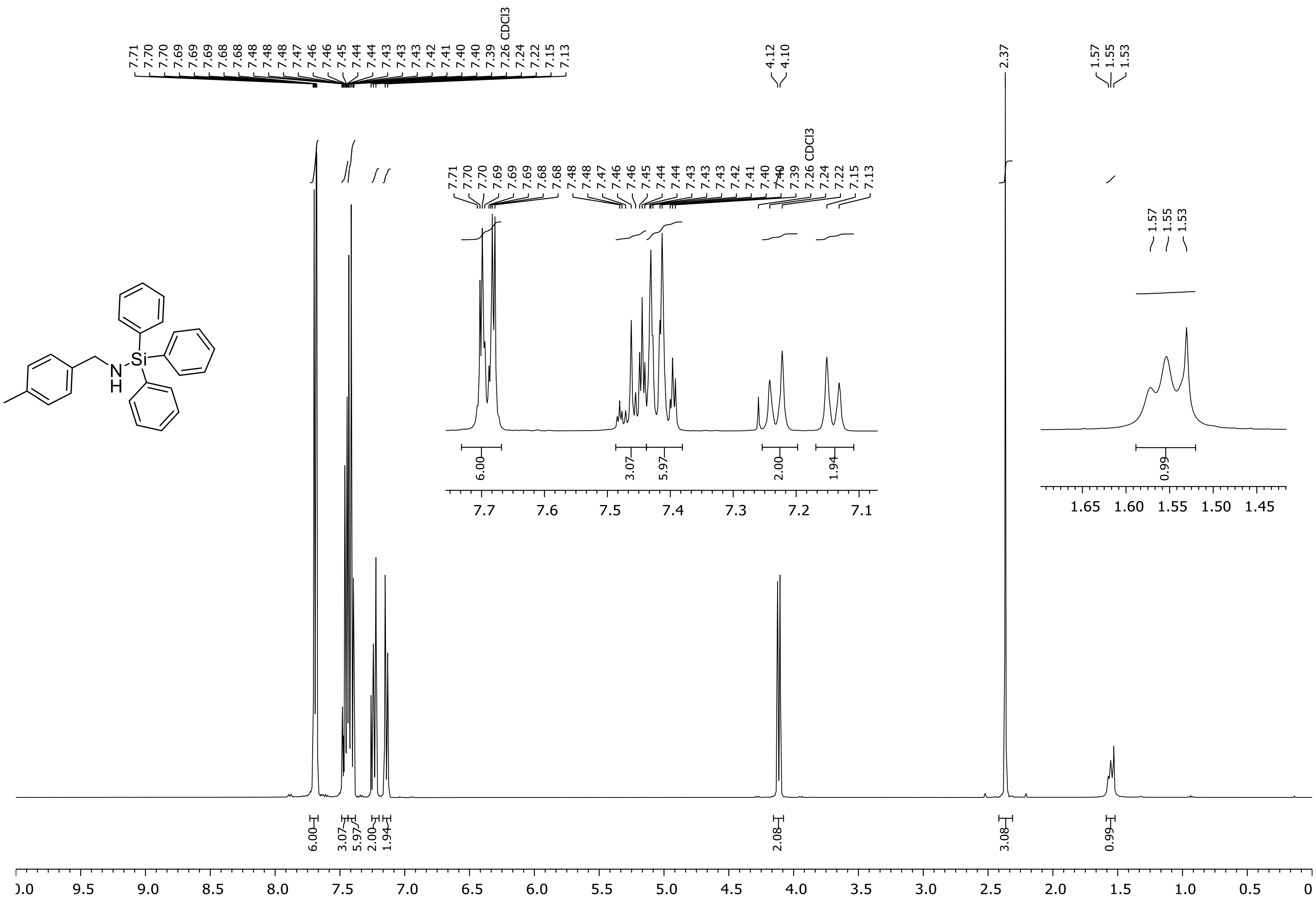

$^{13}C\{^{1}H\}$ NMR spectrum of *N*-(4-methylbenzyl)-1,1,1-triphenylsilanamine (9) (101 MHz, $CDCl_3$)

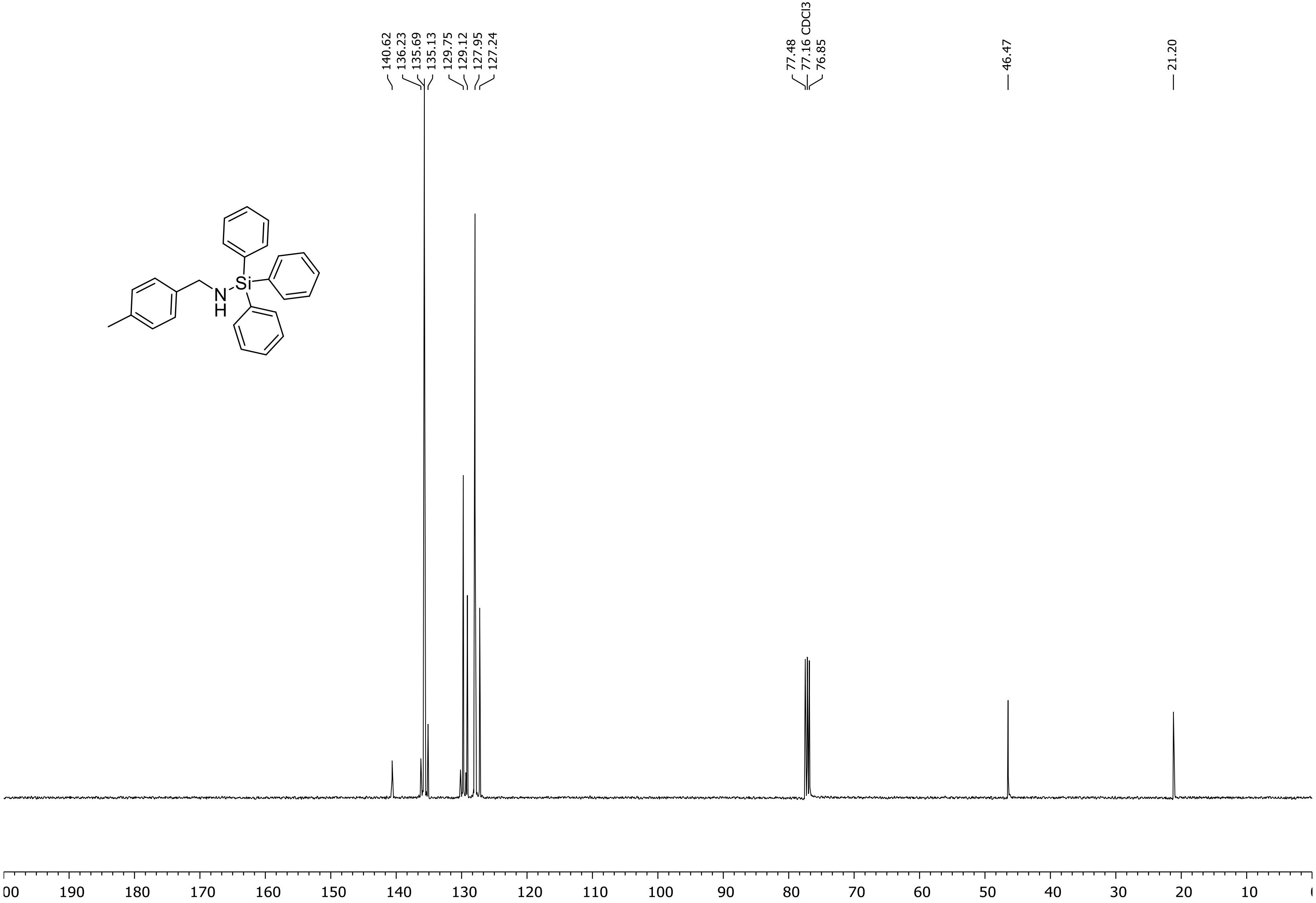

^{1}H – ^{29}Si HMBC NMR spectrum of *N*-(4-methylbenzyl)-1,1,1-triphenylsilanamine (9) (400-80 MHz, $CDCl_3$)

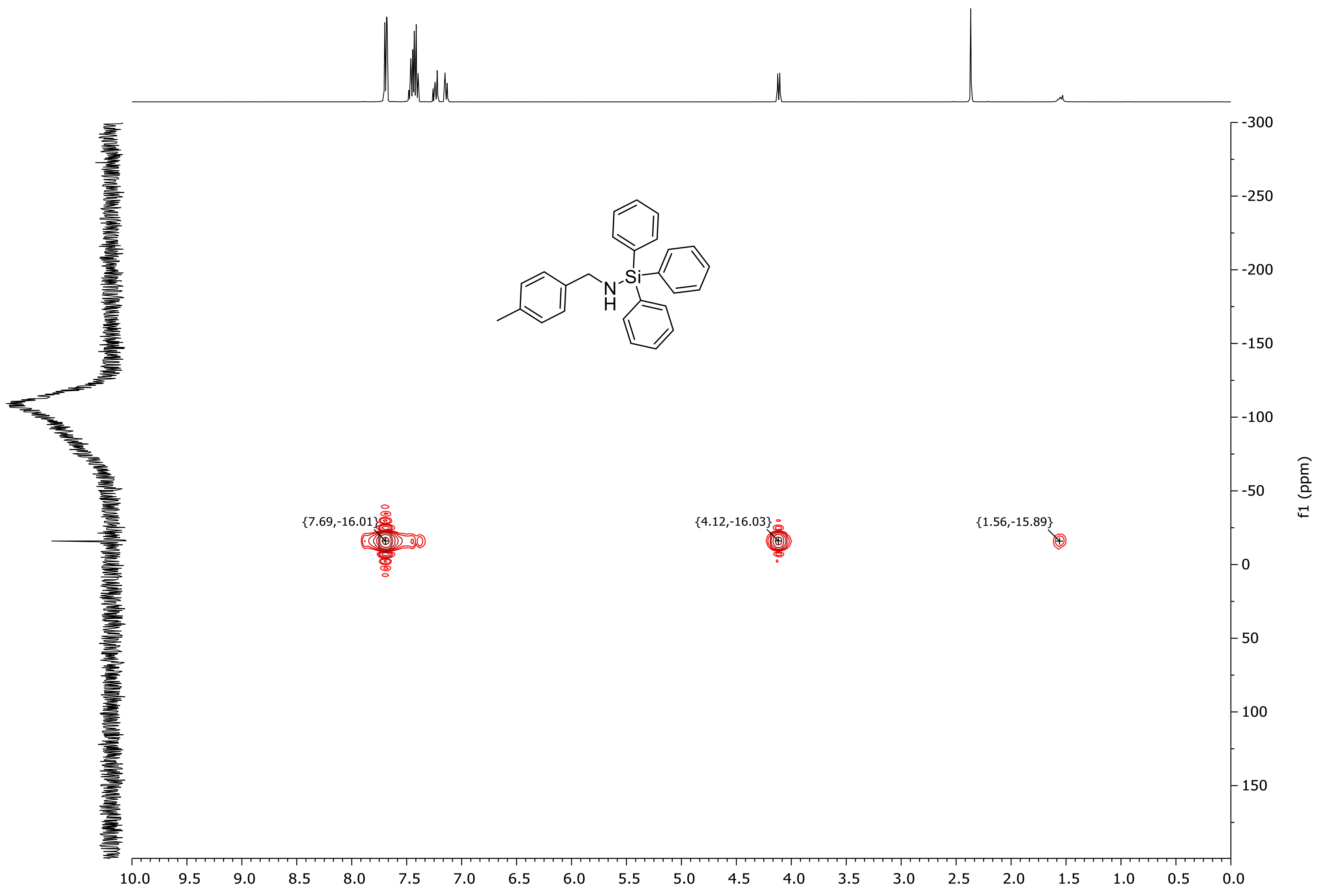

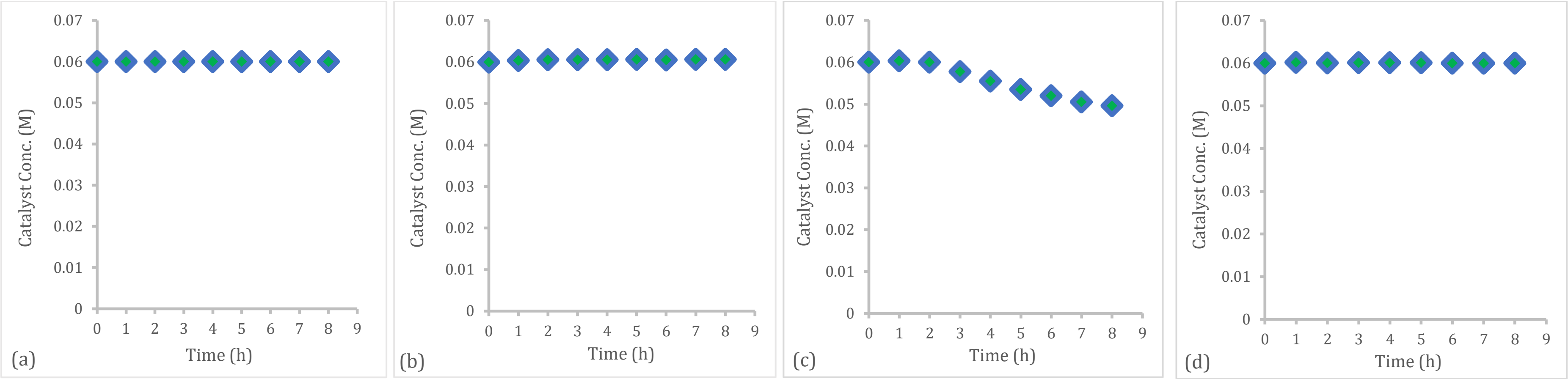

Figure S1. Catalyst integrity plots of [**6h**] vs time in refluxing toluene. Left-to-right: (a) catalyst alone [**6h**]$_0$ = 0.06 M; (b) with added acid **1a** [0.2 M]; (c) with added amine **2a** [0.2 M]; (d) with added amide **3a** [0.2 M].

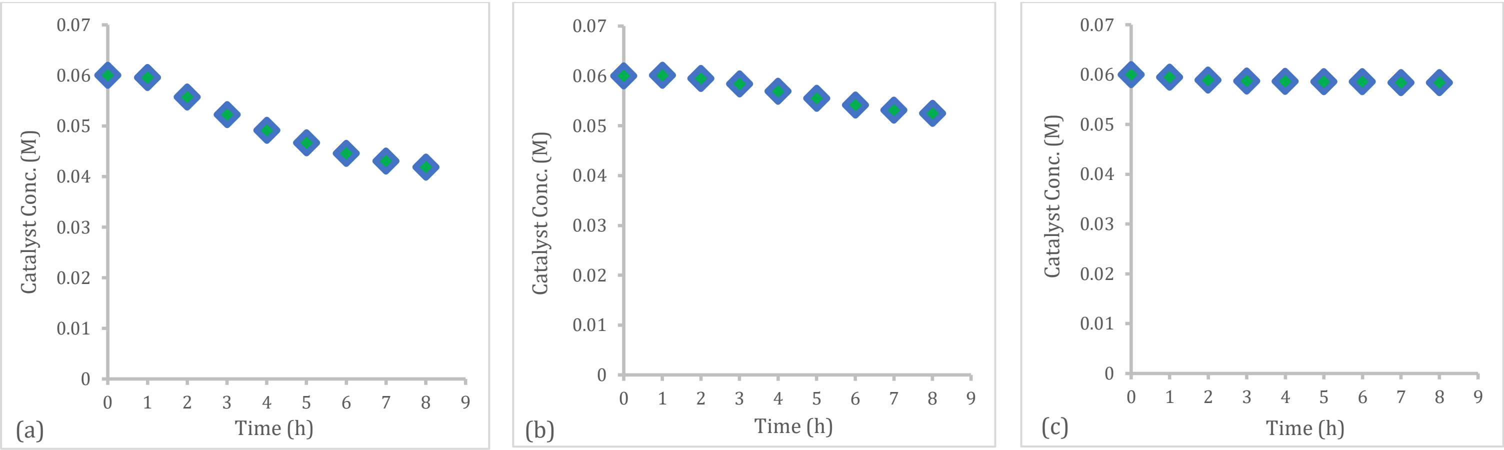

Figure S2. Catalyst integrity plots of [**6h**] vs time in refluxing toluene. Left-to-right: [**6h**]$_0$ = 0.06 M (a) with added *N*-methylbenzylamine [0.2 M]; (b) with added morpholine [0.2 M]; (c) with added aniline [0.2 M].

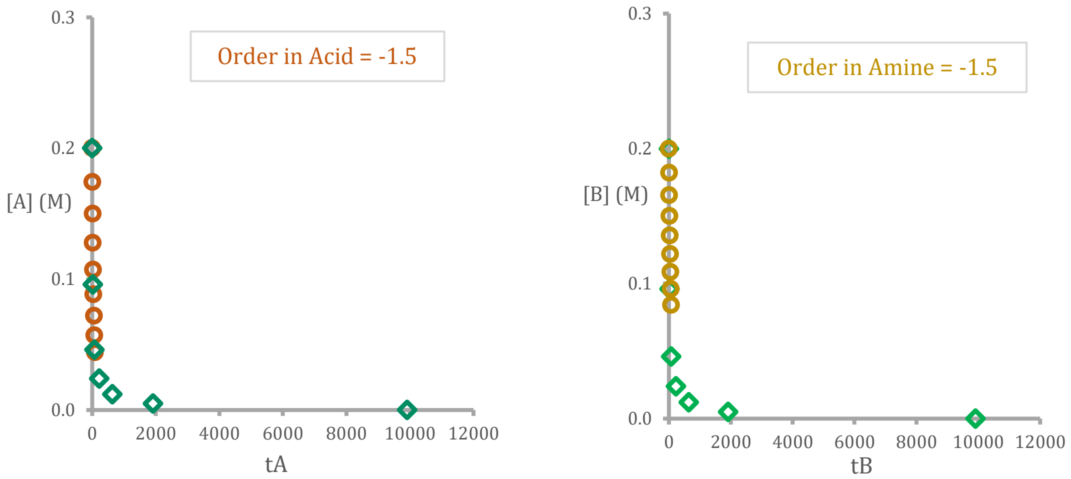

Figure S3. VTNA plots of concentration vs normalised timescale from different excess experiments using acid **1a**, amine **2a** with catalyst **6h** to determine order (left) in acid **1a** and (right) in amine **2a**.

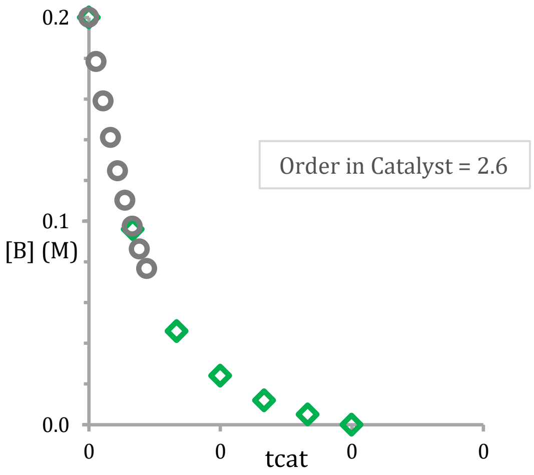

Figure S4. VTNA plot of concentration vs normalised timescale using acid **1a**, amine **2a** with catalyst **6h** to determine order in catalyst **6h**.

Table S1. Crystal Data, Data Collection and Refinement Parameters for the structures of **5e, 5f, 6d, 6e, 6e-p2, 6e-p3, 6f, 6h, 6k, 7, 9, 11, 12** and disiloxanes of **6e, 6h** & **6i**.

Data	5e	5f	6d	6e
**Formula**	$C_{18}H_{13}F_3Si$	$C_{18}H_{13}Cl_3Si$	$C_{30}H_{40}OSi$	$C_{18}H_{13}F_3OSi$
**Solvent**	—	—	—	—
**Formula weight**	314.37	363.72	444.71	330.37
**Colour, habit**	colorless tablets	colorless blocks	colourless blocks	colorless blocks
**Temperature / K**	173	173	173	173
**Crystal system**	orthorhombic	orthorhombic	triclinic	monoclinic
**Space group**	*Pbca* (no. 61)	$P2_12_12_1$ (no. 19)	*P*-1 (no. 2)	*C*2/*c* (no. 15)
**a / Å**	10.7908(4)	8.8393(2)	11.7733(6)	20.0412(7)
***b* / Å**	8.6862(3)	11.0368(3)	11.7787(6)	16.0311(5)
***c* / Å**	33.2183(15)	18.0391(5)	11.8716(6)	21.3364(7)
**α / deg**	90	90	102.265(5)	90
**β / deg**	90	90	112.078(5)	108.264(4)
**γ / deg**	90	90	111.731(5)	90
**V / Å^3**	3113.6(2)	1759.84(8)	1293.55(13)	6509.7(4)
Z	8	4	2	16 [c]
**D_c / gcm^{-3}**	1.341	1.373	1.142	1.348
**radiation used**	Mo-Kα	Cu-Kα	Cu-Kα	Mo-Kα
**μ / mm^{-1}**	0.175	5.300	0.927	0.175
**No. of unique reflns**				57
**measured (R_{int})**	3696 (0.0389)	3485 (0.0466)	9354 (0.0270)	6510 (0.0144)
**obs, $\|F_o\| > 4\sigma(\|F_o\|)$**	2946	2944	5065	5124
**completeness (%) [a]**	99.9	100.0	99.3	98.7
**No. of variables**	203	204	306	437
**R_1(obs), wR_2(all) [b]**	0.0533, 0.1122	0.0373, 0.0899	0.0429, 0.1187	0.0462, 0.1254
**CCDC code**	2247808	2247809	2247810	2247811

[a] Completeness to 0.84 Å resolution. [b] $R_1 = \Sigma||F_o| - |F_c||/\Sigma|F_o|$; $wR_2 = \{\Sigma[w(F_o^2 - F_c^2)^2] / \Sigma[w(F_o^2)^2]\}^{1/2}$; $w^{-1} = \sigma^2(F_o^2) + (aP)^2 + bP$. [c] There are two crystallographically independent molecules. [d] There are six

crystallographically independent molecules, five in general positions and one adjacent to a C_3 axis. [e] There are two crystallographically independent molecules, one in a general position and one adjacent to a C_3 axis. [f] There are four crystallographically independent molecules.

Table S1. part 2

Data	6e-p2	6e-p3	6f	6h
**Formula**	$C_{18}H_{13}F_3OSi$	$C_{18}H_{13}F_3OSi$	$C_{18}H_{13}Cl_3OSi$	$C_{18}H_{13}Br_3OSi$
**Solvent**	—	—	—	—
**Formula weight**	330.37	330.37	379.72	513.10
**Colour, habit**	colourless blocky needles	colourless blocky needles	colorless blocks	colorless blocks
**Temperature / K**	173	173	173	173
**Crystal system**	trigonal	trigonal	trigonal	triclinic
**Space group**	*R*3 (no. 146)	*R*3 (no. 146)	*R*3 (no. 146)	*P*1 (no. 1)
***a* / Å**	44.7390(16)	22.3664(13)	47.0387(10)	11.4531(9)
***b* / Å**	44.7390(16)	22.3664(13)	47.0387(10)	13.9038(9)
***c* / Å**	11.1604(4)	11.1521(7)	11.3591(3)	14.3428(8)
**α / deg**	90	90	90	111.169(6)
**β / deg**	90	90	90	107.660(6)
**γ / deg**	120	120	120	102.534(6)
**V / Å^3**	19345.7(16)	4831.4(6)	21766.2(10)	1887.4(2)
Z	48 [d]	12 [e]	48 [d]	4 [f]
**D_c / gcm^{-3}**	1.361	1.363	1.391	1.806
**radiation used**	Mo-Kα	Mo-Kα	Mo-Kα	Mo-Kα
**μ / mm^{-1}**	0.177	0.177	0.572	6.476
**No. of unique reflns**				
**measured (R_{int})**	10323 (0.0231)	4275 (0.0176)	21255 (0.0541)	14970
**obs, $\|F_o\| > 4\sigma(\|F_o\|)$**	7487	3048	13621	7199
**completeness (%)** [a]	98.4	99.9	99.9	99.8
**No. of variables**	1199	286	1199	833
**R_1(obs), wR_2(all)** [b]	0.0744, 0.2135	0.0889, 0.2490	0.1190, 0.3303	0.0699, 0.1421
**CCDC code**	2247812	2247813	2247756	2247757

Table S1. part 3

Data	6h-p2	6k	7	9
**Formula**	$C_{18}H_{13}Br_3OSi$	$C_{18}H_7F_9OSi$	$C_{26}H_{22}O_2Si$	$C_{26}H_{25}NSi$
**Solvent**	—	—	—	—
**Formula weight**	513.10	438.33	394.52	379.56
**Colour, habit**	colorless blocky needles	colourless blocks	colorless blocks	colourless blocks
**Temperature / K**	173	173	173	173
**Crystal system**	trigonal	monoclinic	monoclinic	triclinic
**Space group**	*R*3 (no. 146)	*C*2/*c* (no. 15)	$P2_1/n$ (no. 14)	*P*-1 (no. 2)
a **/ Å**	47.960(3)	14.6631(2)	9.7227(3)	9.8329(7)
b **/ Å**	47.960(3)	24.8584(2)	18.8868(7)	10.7135(7)
c **/ Å**	11.4530(6)	19.5410(2)	11.3976(4)	11.8408(8)
**α / deg**	90	90	90	72.719(6)
**β / deg**	90	101.305(1)	94.596(3)	71.113(6)
**γ / deg**	120	90	90	65.366(7)
V **/ Å**^{3}	22815(3)	6984.52(13)	2086.22(13)	1053.56(14)
Z	48 [d]	16 [c]	4	2
D_c **/ gcm**$^{-3}$	1.793	1.667	1.256	1.196
**radiation used**	Cu-Kα	Cu-Kα	Mo-Kα	Mo-Kα
**μ / mm**$^{-1}$	8.479	2.148	0.132	0.122
**No. of unique reflns**				
**measured (**R_{int}**)**	10561 (0.0540)	10826 (0.0185)	4182 (0.0190)	6073 (0.0206)
**obs,** $\|F_o\| > 4\sigma(\|F_o\|)$	7386	6714	3364	4142
**completeness (%)** [a]	97.8	98.4	98.9	98.2
**No. of variables**	1199	531	263	254
R_1**(obs),** wR_2**(all)** [b]	0.0996, 0.2983	0.0480, 0.1358	0.0402, 0.1030	0.0494, 0.115
**CCDC code**	2247758	2247759	2247760	2247761

Table S1. part 4

Data	11	12	Disiloxane of **6e**	Disiloxane of **6h**
**Formula**	$C_8H_7O_2{\cdot}C_8H_{12}N$	$C_{29}H_{30}O_2Si$	$C_{36}H_{24}F_6OSi_2$	$C_{36}H_{24}Br_6OSi_2$
**Solvent**	—	—	—	—
**Formula weight**	257.32	438.62	642.73	1008.19
**Colour, habit**	colorless blocky needles	colourless blocks	colourless blocks	colourless blocks
**Temperature / K**	173	173	173	173
**Crystal system**	monoclinic	triclinic	monoclinic	orthorhombic
**Space group**	$P2_1$ (no. 4)	P-1 (no. 2)	$P2_1/c$ (no. 14)	$P2_12_12_1$ (no. 19)
a **/ Å**	4.7680(3)	10.2852(5)	8.5265(1)	19.45536(12)
b **/ Å**	24.841(3)	10.9501(6)	19.0729(2)	19.52646(12)
c **/ Å**	5.6840(4)	11.1906(8)	10.2554(1)	19.54344(13)
**α / deg**	90	78.912(5)	90	90
**β / deg**	96.607(6)	76.733(5)	108.993(1)	90
**γ / deg**	90	71.352(5)	90	90
V **/ Å**^{3}	668.76(9)	1152.59(13)	1576.99(3)	7424.44(8)
Z	2	2	2	8 [c]
D_c **/ gcm**$^{-3}$	1.278	1.264	1.354	1.804
**radiation used**	Mo-Kα	Mo-Kα	Cu-Kα	Cu-Kα
**μ / mm**$^{-1}$	0.084	0.126	1.583	8.652
**No. of unique reflns**				
**measured (**R_{int}**)**	1755 (0.0192)	6740 (0.0175)	5182 (0.0252)	14967 (0.0499)
**obs,** $\lvert F_o\rvert > 4\sigma(\lvert F_o\rvert)$	1580	4539	3048	13524
**completeness (%)** [a]	98.5	98.6	99.6	100
**No. of variables**	185	289	242	811
R_1**(obs),** wR_2**(all)** [b]	0.0369, 0.0896	0.0458, 0.1120	0.0445, 0.1164	0.0304, 0.0736
**CCDC code**	2247762	2247763	2247764	2247765

Table S1. part 5

**Data**	Disiloxane of **6i**
**Formula**	$C_{42}H_{24}F_{18}OSi_2$
**Solvent**	—
**Formula weight**	942.79
**Colour, habit**	colourless blocks
**Temperature / K**	173
**Crystal system**	monoclinic
**Space group**	$P2_1/c$ (no. 14)
**a / Å**	9.2728(5)
***b* / Å**	21.5149(8)
***c* / Å**	9.8260(4)
**α / deg**	90
**β / deg**	102.723(4)
**γ / deg**	90
***V* / Å³**	1912.19(15)
Z	2
D_c **/ gcm⁻³**	1.637
**radiation used**	Mo-Kα
**μ / mm⁻¹**	0.217
**No. of unique reflns**	
**measured** (R_{int})	10851 (0.0322)
**obs,** $\lvert F_o \rvert > 4\sigma(\lvert F_o \rvert)$	4020
**completeness (%)** [a]	99.8
**No. of variables**	314
R_1**(obs),** wR_2**(all)** [b]	0.0510, 0.1204
**CCDC code**	2247766

Table S1 provides a summary of the crystallographic data for the structures of **5e**, **5f**, **6d**, **6e**, **6e-p2**, **6e-p3**, **6f**, **6h**, **6h-p2**, **6k**, 7, **9**, **11**, **12**, and disiloxanes of **6e**, **6h** & **6i**. Data were collected using Agilent Xcalibur 3 E (**5e**, **6e**, **6e-p2**, **6e-p3**, **6f**, **6h**, 7, **9**, **11**, **12** and disiloxane of **6i**) and Xcalibur PX

Ultra A (**5f**, **6d**, **6h-p2**, **6k** and disiloxanes of **6e** & **6h**) diffractometers, and the structures were solved and refined using the OLEX2,[X1] SHELXTL[X2] and SHELX-2013[X3] program systems. The absolute structures of **5f**, **6f**, **6h** and disiloxane of **6h** were unambiguously determined by use of the Flack parameter [*x* = –0.014(11), 0.03(2) and –0.018(10) respectively], whilst those of **6e-p2**, **6e-p3**, **6h-p2** and **11** could not be unambiguously determined [*x* = 0.04(10), 0.00(5), 0.01(5) and –1.4(10) respectively].

The X-ray crystal structure of 5e

The Si1–H hydrogen atom in the structure of **5e** was located from a Δ*F* map and refined freely.

The X-ray crystal structure of 5f

The Si1–H hydrogen atom in the structure of **5f** was located from a Δ*F* map and refined freely.

The X-ray crystal structure of 6d

Silanol **6d** exists as a hydrogen bonded dimer in the solid state, with the asymmetric unit containing one molecule of silanol. The OH proton was visible in Δ*F* maps which showed two distinct positions, one between the two oxygen atoms of the dimer and one pointing away from the other molecule in the dimer. These were modelled with a fixed occupancy of 50% each but allowed to refine freely subject to an O–H distance constraint.

The X-ray crystal structure of 6e

The first of three polymorphs of *tris*(4-fluorophenyl)silanol reported here, the structure of **6e** was found to contain two crystallographically independent molecules (**6e-A** and **6e-B**) in the asymmetric unit. Inspection of the packing of the two independent molecules shows them to form a C_2-symmetric four molecule cluster held together by a series of O–H···O hydrogen bonds. The positions of these O–H hydrogen atoms were clearly visible from Δ*F* maps which showed electron density in the appropriate positions *either side* of each unique oxygen atom, corresponding to the four hydrogen bond sequence going both possible ways around the cycle, *i.e.*, ···O–H···O–H··· and –O···H–O···H–. The O–H hydrogen atoms were refined freely subject to an O–H distance constraint of 0.90 Å, and the relative occupancy of the two sequences was estimated to be 51:49 by restraining their thermal parameters to be similar. The C7B-based *p*-fluorophenyl group was found to be disordered. Two orientations were identified of *ca.* 79 and 21% occupancy, their geometries were optimized, the thermal parameters of adjacent atoms were restrained to be similar, and only the non-hydrogen atoms

of the major occupancy orientation were refined anisotropically (those of the minor occupancy orientation were refined isotropically).

The X-ray crystal structure of 6e-p2

The second of the three polymorphs of *tris*(4-fluorophenyl)silanol reported here, the structure of **6e-p2** was found to contain six crystallographically independent molecules in the asymmetric unit, five in general positions (**6e-p2-A**, **6e-p2-B**, **6e-p2-C**, **6e-p2-D** and **6e-p2-E**) and one adjacent to a C_3 axis (**6e-p2-F**). Molecules **6e-p2-B**, **6e-p2-D**, **6e-p2-E** and **6e-p2-F** were all found to be disordered. For the three in general positions (**6e-p2-B**, **6e-p2-D** and **6e-p2-E**) two orientations of the whole molecule were identified of *ca.* 87:13, 76:24 and 70:39% occupancy respectively, the geometries of each pair of orientations were optimized, the thermal parameters of adjacent atoms were restrained to be similar, and only the non-hydrogen atoms of the major occupancy orientations were refined anisotropically (those of the minor occupancy orientations were refined isotropically). Molecule **6e-p2-F** was found to be disordered across a C_3 axis, and this was modelled by using one complete, 33% occupancy orientation, with two further orientations being generated by operation of the C_3 axis. The geometry of the unique orientation was optimized, and only the silicon and fluorine atoms were refined anisotropically (the rest were refined isotropically). None of the O–H hydrogen atoms could be reliably located from ΔF maps, and so they were added in calculated positions with a O–H distance of 0.90 Å.

The X-ray crystal structure of 6e-p3

The third of the three polymorphs of *tris*(4-fluorophenyl)silanol reported here, **6e-p3** crystallizes in a rhombohedral unit cell very similar to that seen for the second polymorph **6e-p2**, but with the *a* and *b* axis lengths very close to half of those seen in **6e-p2** ($a = b = 22.3664(13)$ Å here, *cf.* $a = b = 44.7390(16)$ Å for **6e-p2**). Attempts to re-index the data for **6e-p2** using this smaller unit cell were not successful with less than 30% of the observed peaks fitting to this cell. The structure of **6e-p3** was found to contain two crystallographically independent molecules in the asymmetric unit, one in a general position (**6e-p3-A**) and one adjacent to a C_3 axis (**6e-p2-B**). Both molecules were found to be disordered. For molecule **6e-p3-A** in a general position two orientations of the whole molecule were identified of *ca.* 65 and 35% occupancy, their geometries were optimized, the thermal parameters of adjacent atoms were restrained to be similar, and only the non-hydrogen atoms of the major occupancy orientation were refined anisotropically (those of the minor occupancy orientation were

refined isotropically). Molecule **6e-p3-B** was found to be disordered across a C_3 axis, and this was modelled by using one complete, 33% occupancy orientation, with two further orientations being generated by operation of the C_3 axis. The geometry of the unique orientation was optimized, and all of the atoms were refined isotropically. None of the O–H hydrogen atoms could be reliably located from ΔF maps, and so they were added in calculated positions with a O–H distance of 0.90 Å.

The X-ray crystal structure of 6f

Isomorphous with its fluorine analogue **6e-p2**, the structure of the chlorine species **6f** was found to contain six crystallographically independent molecules in the asymmetric unit, five in general positions (**6f-A**, **6f-B**, **6f-C**, **6f-D** and **6f-E**) and one adjacent to a C_3 axis (**6f-F**). In common with the fluorine analogue, molecules **6f-B**, **6f-D**, **6f-E** and **6f-F** were all found to be disordered. For the three in general positions (**6f-B**, **6f-D** and **6f-E**) two orientations of the whole molecule were identified of *ca.* 90:10, 86:14 and 66:34% occupancy respectively, the geometries of each pair of orientations were optimized, the thermal parameters of adjacent atoms were restrained to be similar, and only the non-hydrogen atoms of the major occupancy orientations were refined anisotropically (those of the minor occupancy orientations were refined isotropically). Molecule **6f-F** was found to be disordered across a C_3 axis, and this was modelled by using one complete, 33% occupancy orientation, with two further orientations being generated by operation of the C_3 axis. The geometry of the unique orientation was optimized, and only the silicon and chlorine atoms were refined anisotropically (the rest were refined isotropically). None of the O–H hydrogen atoms could be reliably located from ΔF maps, and so they were added in calculated positions with a O–H distance of 0.90 Å.

The X-ray crystal structure of 6h

The structure of the bromine species **6h** was found to contain four crystallographically independent molecules in the asymmetric unit (**6h-A**, **6h-B**, **6h-C** and **6h-D**). None of the O–H hydrogen atoms could be reliably located from ΔF maps, and so they were added in calculated positions with a O–H distance of 0.90 Å.

The X-ray crystal structure of 6h-p2

Isomorphous with both the fluorine (**6e-p2**) and chlorine (**6f**) analogues, the structure of the bromine species **6h-p2** was found to contain six crystallographically independent molecules in the asymmetric unit, five in general positions (**6h-p2-A**, **6h-p2-B**, **6h-p2-C**, **6h-p2-D** and **6h-p2-E**) and

one adjacent to a C_3 axis (**6h-p2-F**). As was seen for the fluorine and chlorine counterparts, molecules **6h-p2-B**, **6h-p2-D**, **6h-p2-E** and **6h-p2-F** were all found to be disordered. For the three in general positions (**6h-p2-B**, **6h-p2-D** and **6h-p2-E**) two orientations of the whole molecule were identified of *ca.* 84:16, 89:11 and 65:35% occupancy respectively, the geometries of each pair of orientations were optimized, the thermal parameters of adjacent atoms were restrained to be similar, and only the non-hydrogen atoms of the major occupancy orientations were refined anisotropically (those of the minor occupancy orientations were refined isotropically). Molecule **6h-p2-F** was found to be disordered across a C_3 axis, and this was modelled by using one complete, 33% occupancy orientation, with two further orientations being generated by operation of the C_3 axis. The geometry of the unique orientation was optimized, and only the silicon and bromine atoms were refined anisotropically (the rest were refined isotropically). None of the O–H hydrogen atoms could be reliably located from ΔF maps, and so they were added in calculated positions with a O–H distance of 0.90 Å.

The X-ray crystal structure of 6k

Silanol **6k** exists as a hydrogen bonded tetramer in the solid state, with the asymmetric unit containing two symmetry-independent molecules of silanol. Two positions for each of the OH protons were visible in ΔF maps, either side of each unique oxygen atom. These were modelled with a fixed occupancy of 50% each but allowed to refine freely subject to an O–H distance constraint.

The X-ray crystal structure of 9

The N–H hydrogen atom in the structure of **9** was located from a ΔF map but added as an idealized proton using the AFIX 3 instruction. We note that the X-ray crystal structure for $PhCH_2NHSiPh_3$ has previously been reported.[X4]

The X-ray crystal structure of 11

The three N11–H hydrogen atoms in the structure of **11** were all located from ΔF maps and refined freely subject to N–H distance constraints of 0.90 Å. We note that the X-ray crystal structure for $PhCH_2CO_2^{-}\ {}^{+}NH_3CH_2Ph$ has previously been reported.[X5]

The X-ray crystal structure of disiloxane of 6e

One of the phenyl rings in the structure of disiloxane of **6e** exhibited positional disorder across two positions in a ~3:2 ratio. The occupancy was left to refine freely but some DFIX restraints were needed to retain sensible C–Cl bond lengths on the disordered ring.

The X-ray crystal structure of disiloxane of 6h

The structure of the disiloxane of **6h** was found to contain two crystallographically independent molecules in the asymmetric unit (disiloxane of **6h-A** and disiloxane of **6h-B**).

The X-ray crystal structure of disiloxane of 6i

One of the CF_3 groups in the structure of disiloxane of **6i** exhibited positional disorder in two distinct orientations (~2:1 ratio). The occupancy was left to refine freely but restraints were needed to successfully model the disorder.

References

[X1] Dolomanov, O. V.; Bourhis, L. J.; Gildea, R. J.; Howard, J. A.; Puschmann, H. OLEX2: A Complete Structure Solution, Refinement and Analysis Program. *J. Appl. Cryst.* **2009,** *42,* 339-341. DOI: 10.1107/S0021889808042726

[X2] Sheldrick, G. M. SHELXTL, V5. 1, Bruker AXS, Madison, Wisconsin, **1998**.

[X3] Sheldrick, G. M. Crystal Structure Refinement with SHELXL. *Acta Cryst. C,* **2015,** *71,* 3-8. DOI: 10.1107/S2053229614024218

[X4] Bellini, C.; Roisnel, T.; Carpentier, J.-F.; Tobisch, S.; Sarazin, Y. Sequential Barium-Catalysed N-H/H-Si Dehydrogenative Cross-Couplings: Cyclodisilazanes versus Linear Oligosilazanes. *Chem. Eur. J.* **2016,** *22,* 15733-15743. DOI:10.1002/chem.201603191

[X5] Hess, D.; Mayer, P. The Crystal Structures of Benzylammonium Phenylacetate and its Hydrate. *Acta Cryst.* **2019,** *E75,* 194-201. DOI: 10.1107/S2056989019000288

Figures

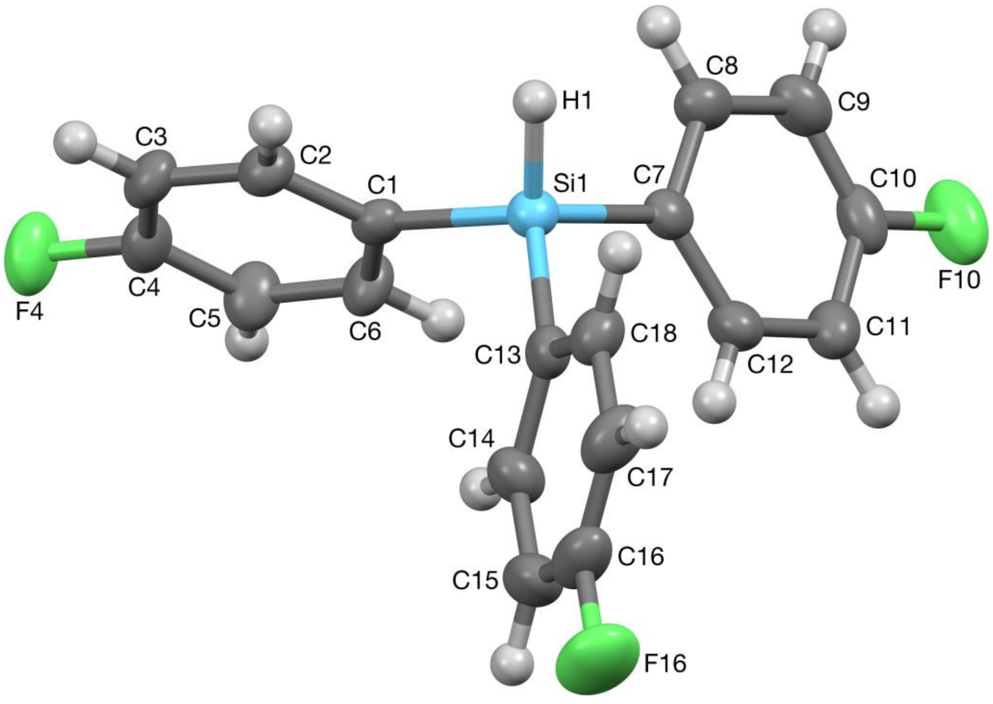

Fig. S1 The crystal structure of **5e** (50% probability ellipsoids).

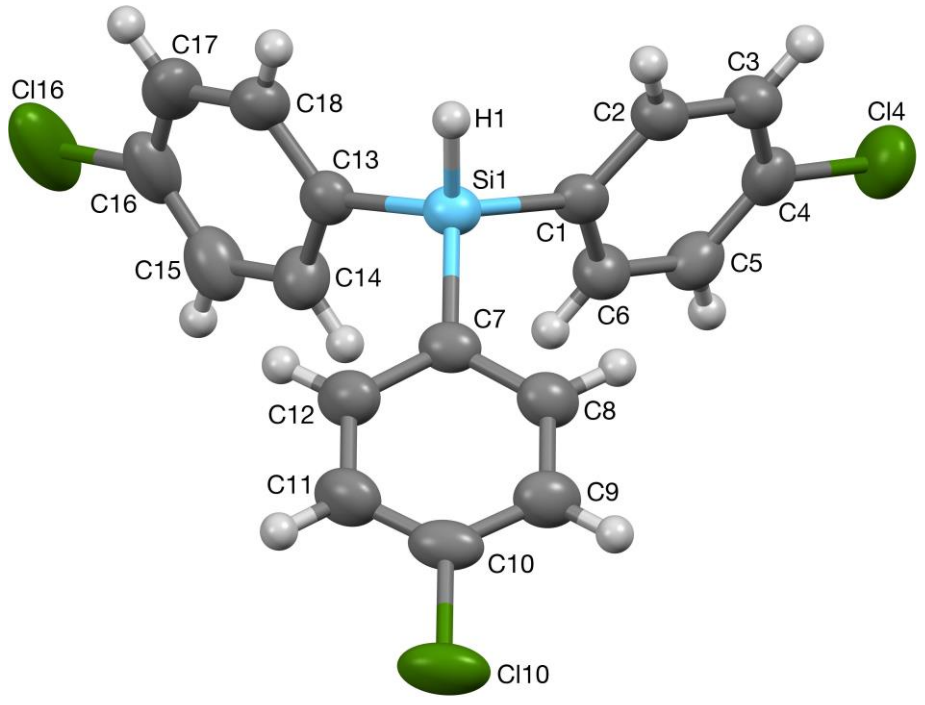

Fig. S2 The crystal structure of **5f** (50% probability ellipsoids).

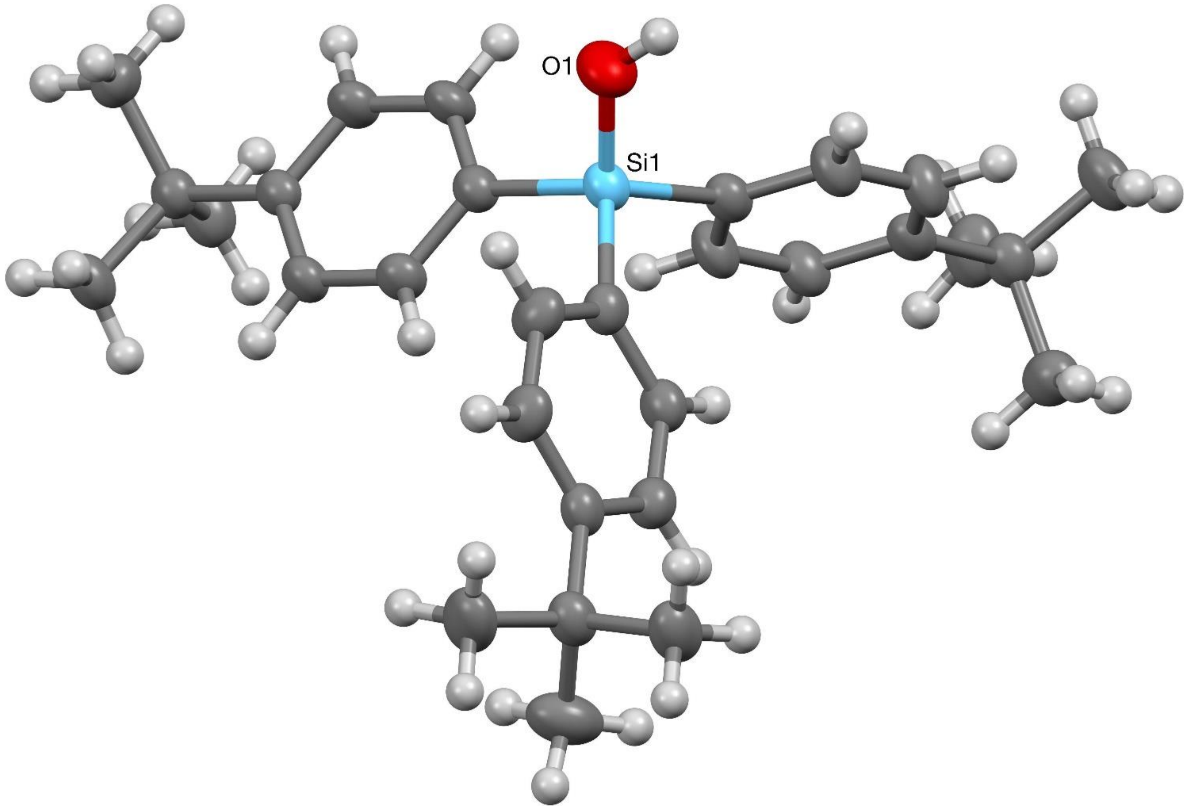

Fig. S3 The crystal structure of **6d** (50% probability ellipsoids).

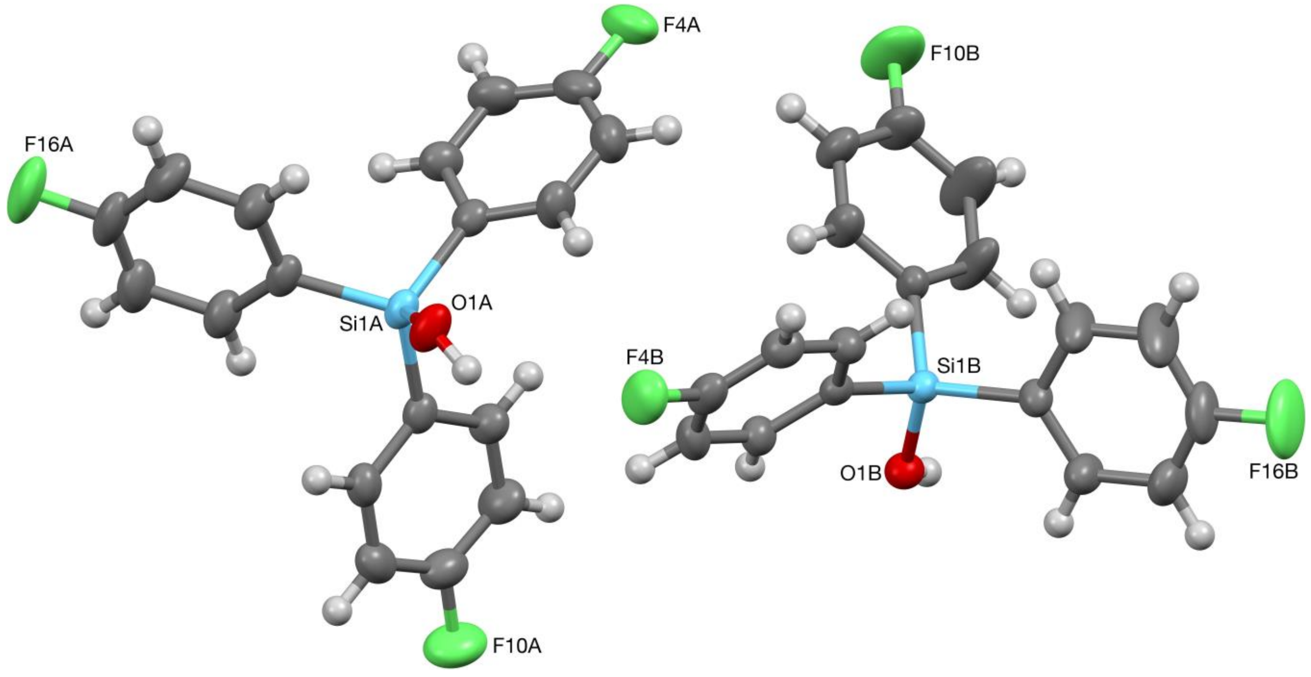

Fig. S4 The structure of **6e** showing the two independent molecules in the asymmetric unit (50% probability ellipsoids).

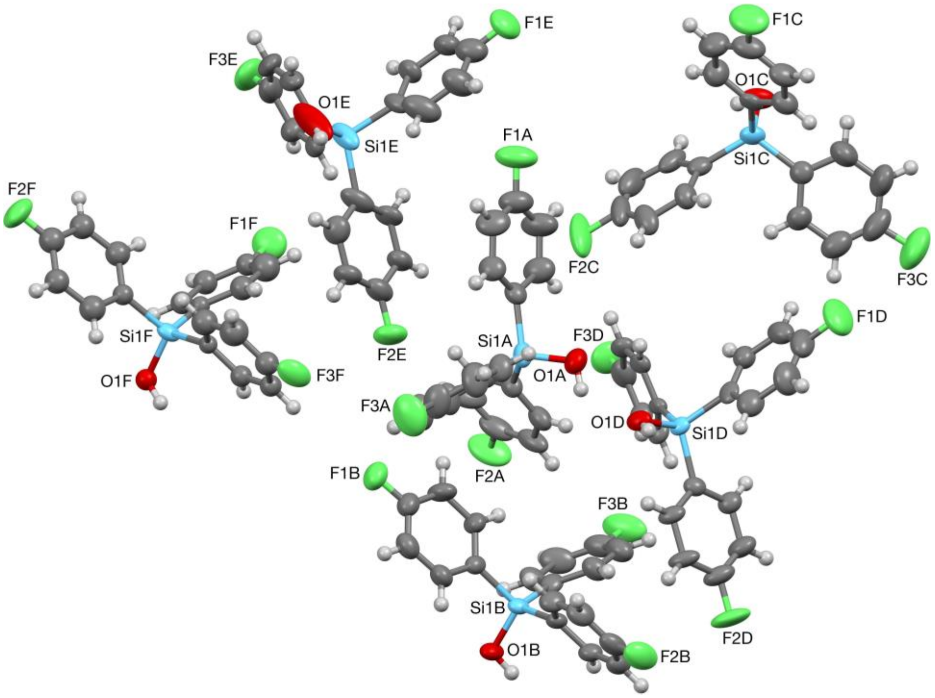

Fig. S5 The structure of **6e-p2** showing the six independent molecules in the asymmetric unit, with molecule **6e-p2-F** sitting adjacent to a C_3 axis (50% probability ellipsoids).

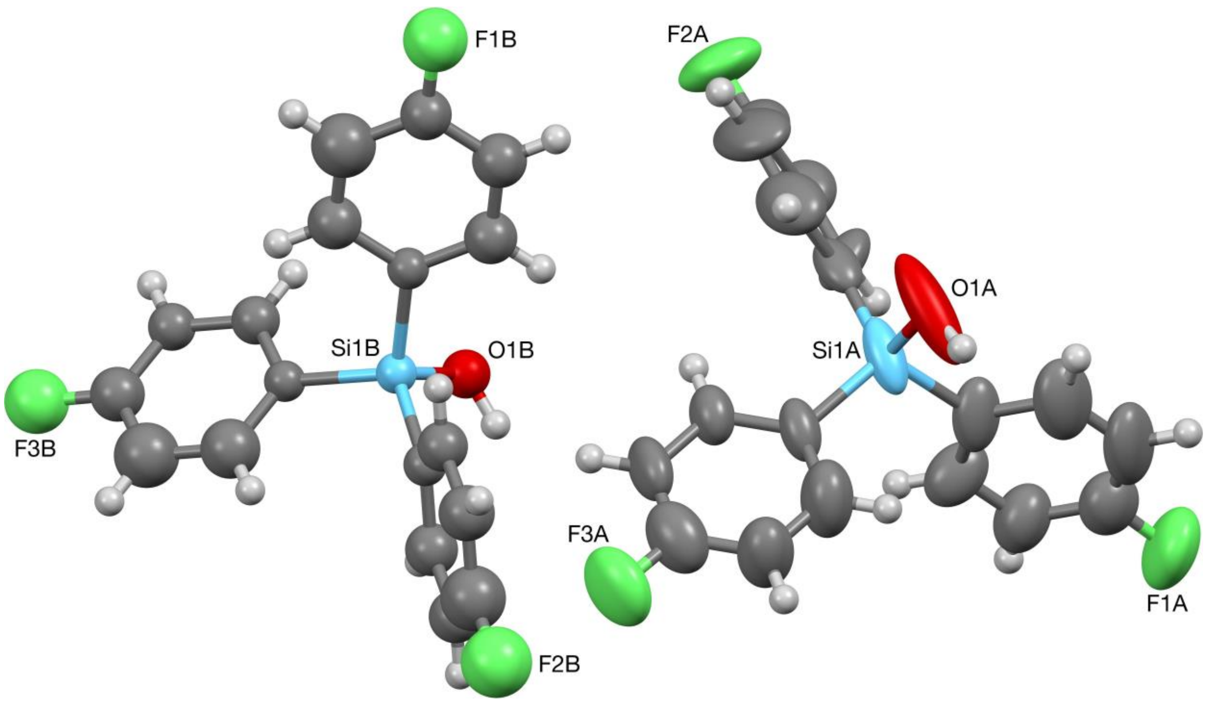

Fig. S6 The structure of **6e-p3** showing the two independent molecules in the asymmetric unit, with molecule **6e-p3-B** sitting adjacent to a C_3 axis (50% probability ellipsoids).

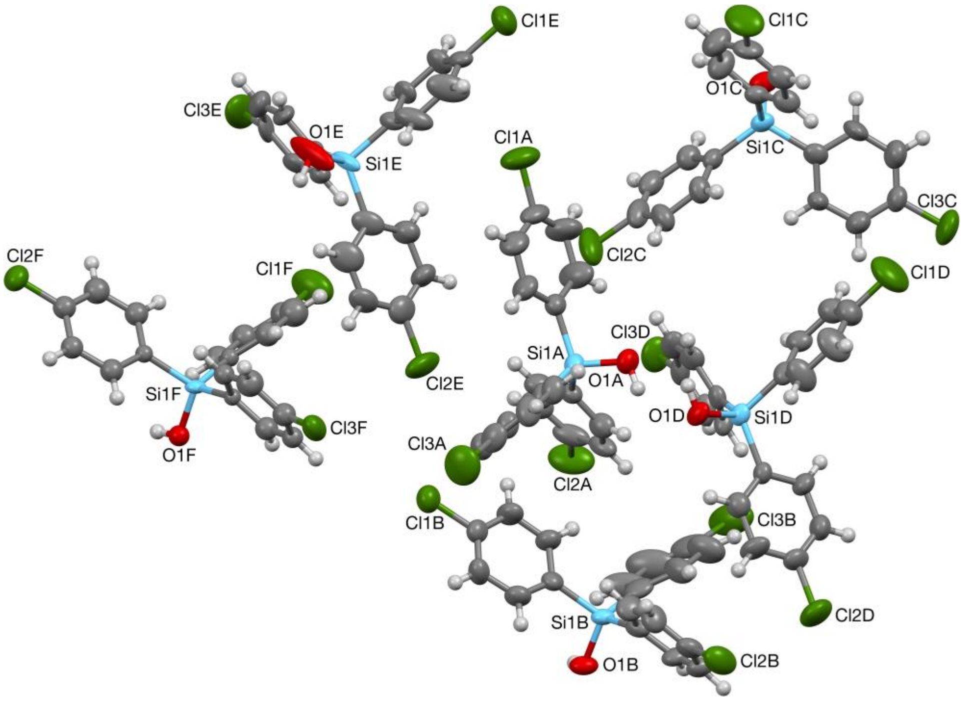

Fig. S7 The structure of **6f** showing the six independent molecules in the asymmetric unit, with molecule **6f-F** sitting adjacent to a C_3 axis (50% probability ellipsoids).

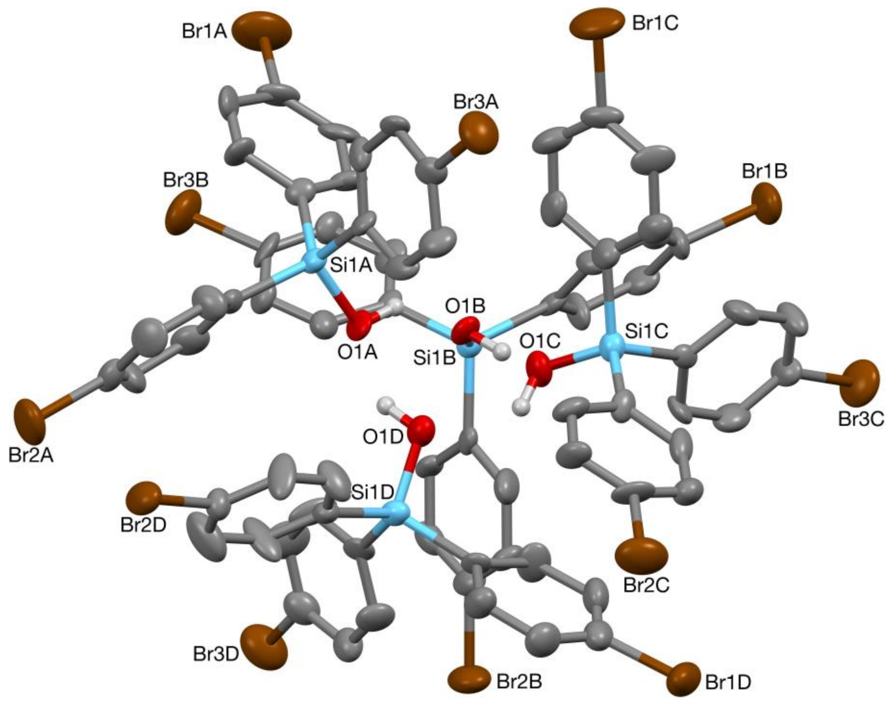

Fig. S8 The structure of **6h** showing the four independent molecules in the asymmetric unit, (50% probability ellipsoids).

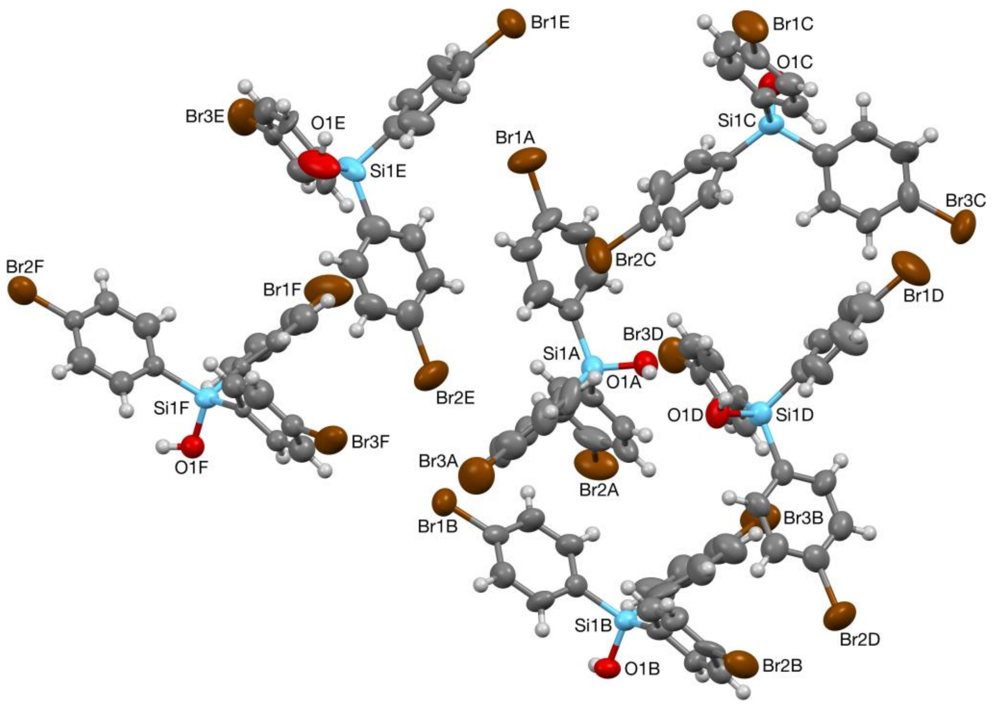

Fig. S9 The structure of **6h-p2** showing the six independent molecules in the asymmetric unit, with molecule **6h-p2-F** sitting adjacent to a C_3 axis (50% probability ellipsoids).

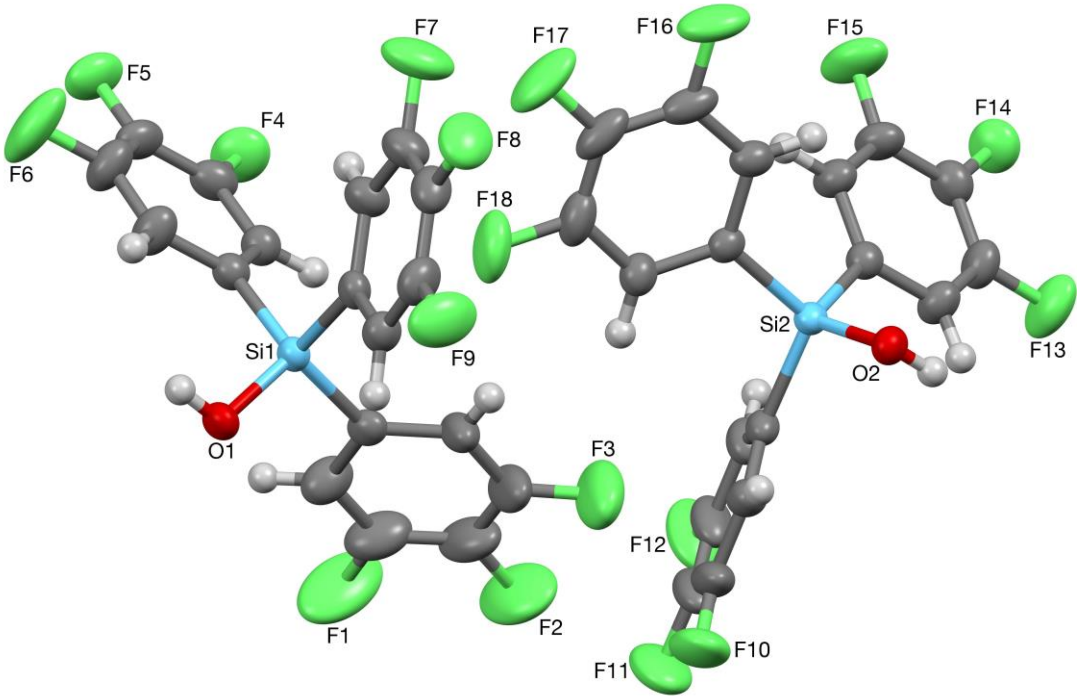

Fig. S10 The structure of **6k** showing the two independent molecules in the asymmetric unit (50% probability ellipsoids).

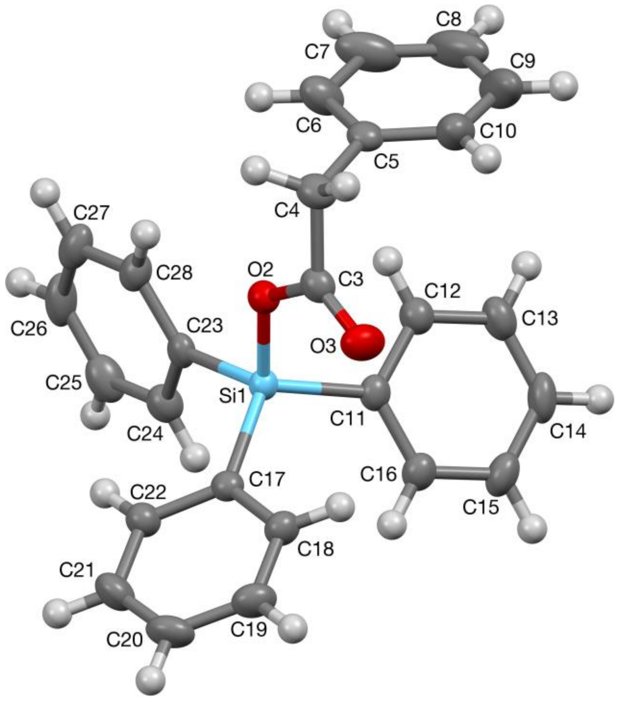

Fig. S11 The structure of **7** (50% probability ellipsoids).

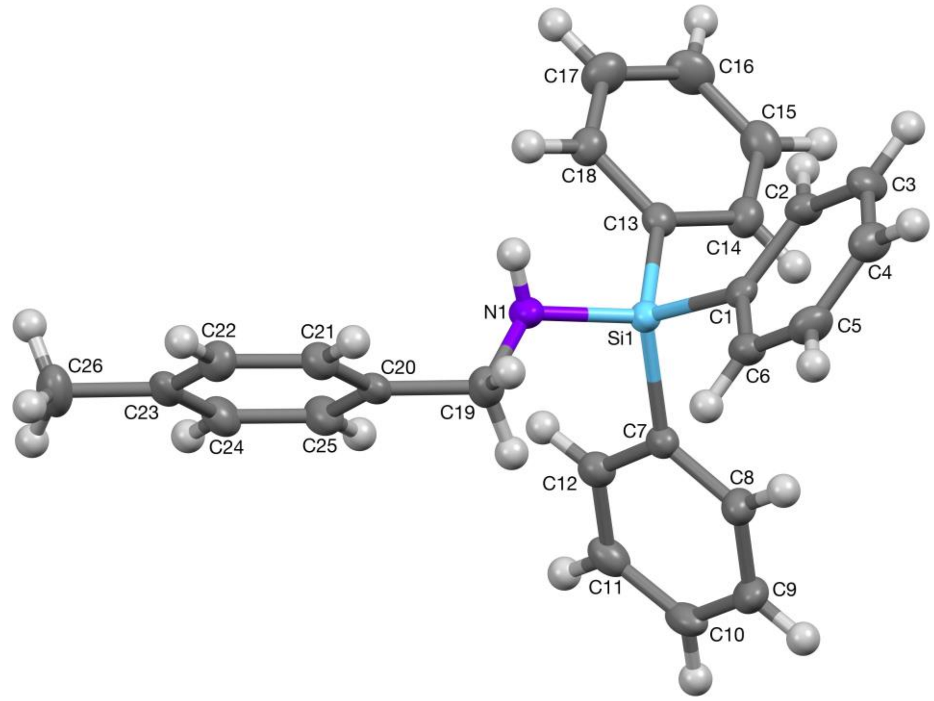

Fig. S12 The structure of **9** (50% probability ellipsoids).

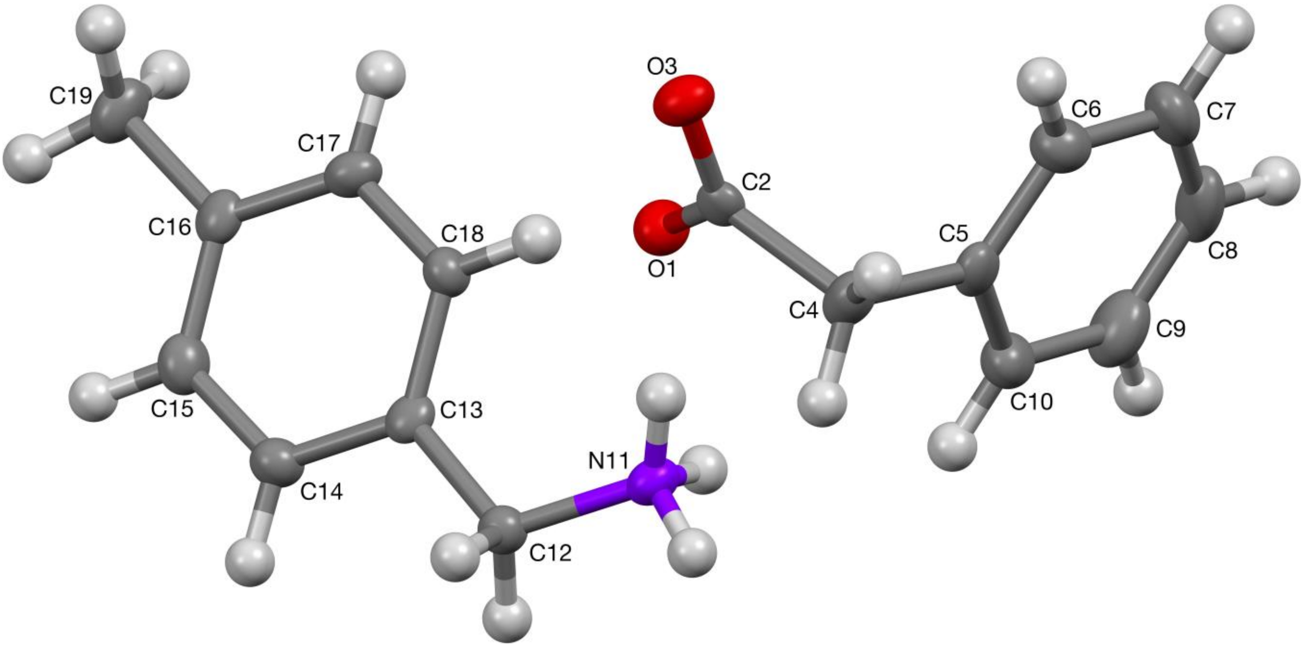

Fig. S13 The structure of **11** (50% probability ellipsoids).

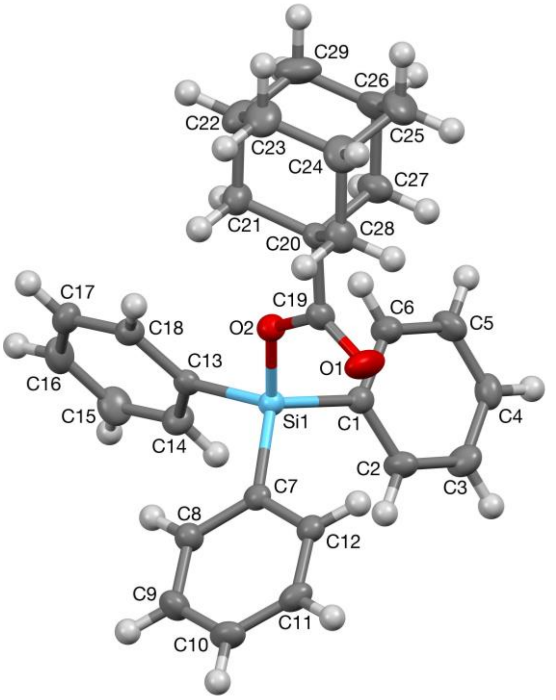

Fig. S14 The structure of **12** (50% probability ellipsoids).

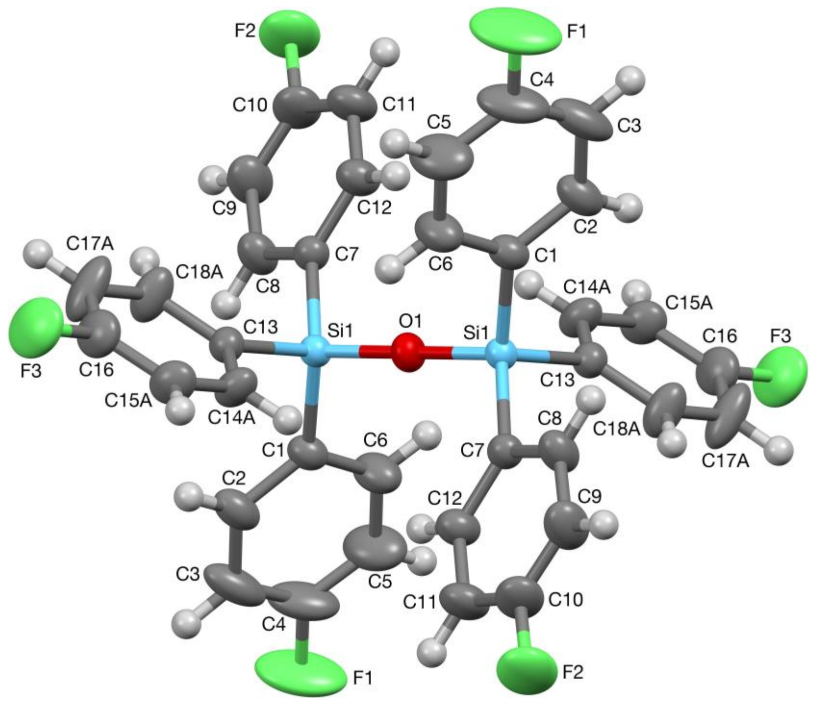

Fig. S15 The structure of siloxane of **6e** which sits across a centre of symmetry at the oxygen atom (50% probability ellipsoids).

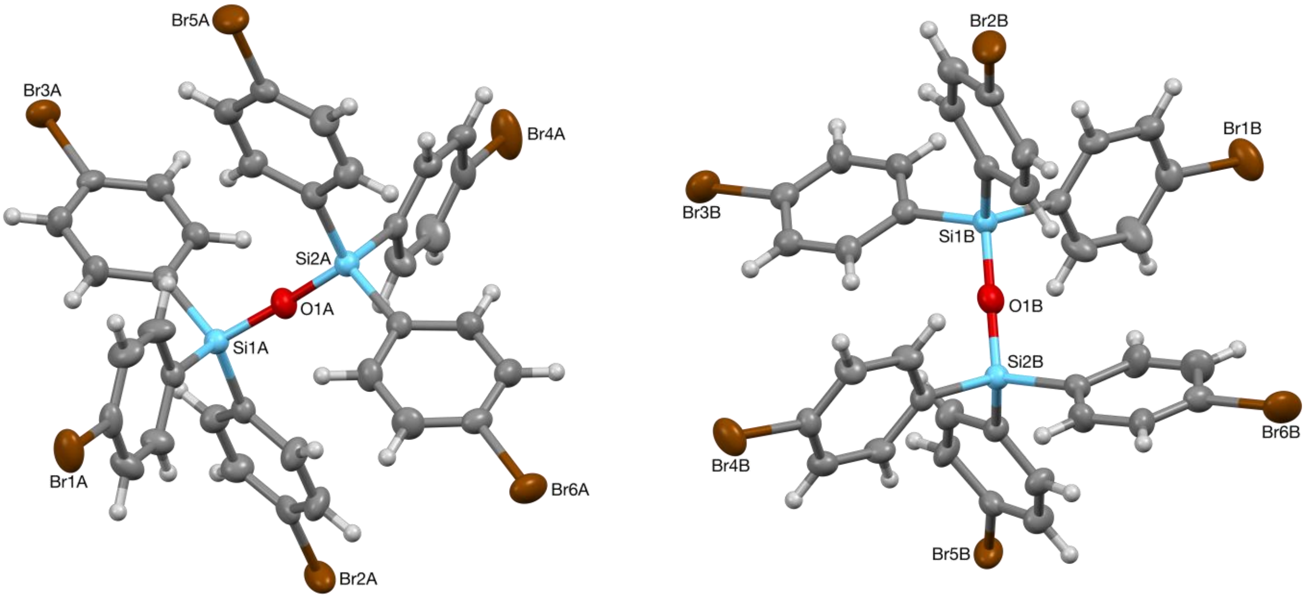

Fig. S16 The structure of siloxane of **6h** showing the two independent molecules in the asymmetric unit (50% probability ellipsoids).

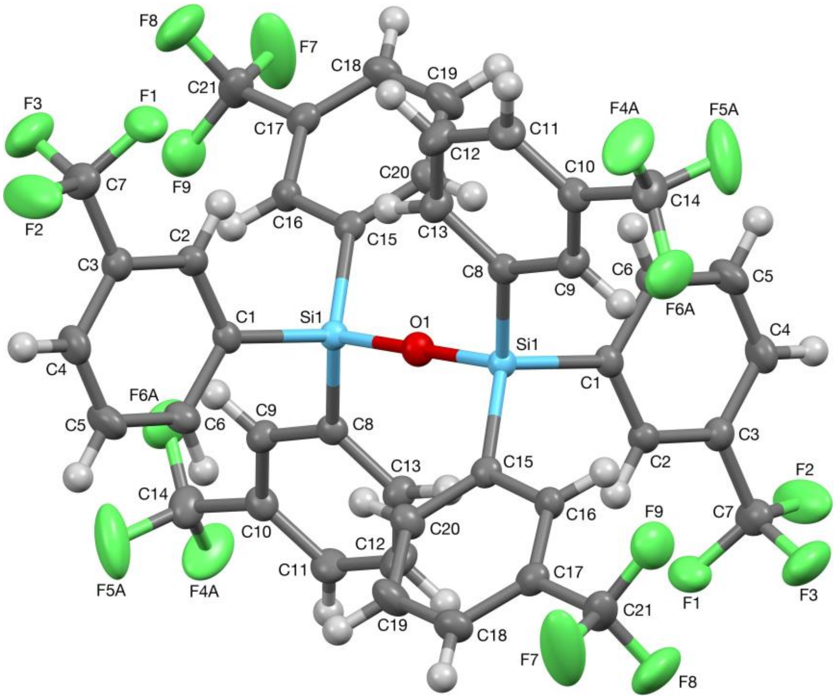

Fig. S17 The crystal structure of siloxane of **6i** which sits across a centre of symmetry at the oxygen atom (50% probability ellipsoids).
